# Characterizing the heterogeneity of tumor tissues from spatially resolved molecular measures

**DOI:** 10.1371/journal.pone.0188878

**Published:** 2017-11-30

**Authors:** John F. Graf, Maria I. Zavodszky

**Affiliations:** GE Global Research, Niskayuna, New York, United States of America; University of Kentucky College of Medicine, UNITED STATES

## Abstract

**Background:**

Tumor heterogeneity can manifest itself by sub-populations of cells having distinct phenotypic profiles expressed as diverse molecular, morphological and spatial distributions. This inherent heterogeneity poses challenges in terms of diagnosis, prognosis and efficient treatment. Consequently, tools and techniques are being developed to properly characterize and quantify tumor heterogeneity. Multiplexed immunofluorescence (MxIF) is one such technology that offers molecular insight into both inter-individual and intratumor heterogeneity. It enables the quantification of both the concentration and spatial distribution of 60+ proteins across a tissue section. Upon bioimage processing, protein expression data can be generated for each cell from a tissue field of view.

**Results:**

The Multi-Omics Heterogeneity Analysis (MOHA) tool was developed to compute tissue heterogeneity metrics from MxIF spatially resolved tissue imaging data. This technique computes the molecular state of each cell in a sample based on a pathway or gene set. Spatial states are then computed based on the spatial arrangements of the cells as distinguished by their respective molecular states. MOHA computes tissue heterogeneity metrics from the distributions of these molecular and spatially defined states. A colorectal cancer cohort of approximately 700 subjects with MxIF data is presented to demonstrate the MOHA methodology. Within this dataset, statistically significant correlations were found between the intratumor AKT pathway state diversity and cancer stage and histological tumor grade. Furthermore, intratumor spatial diversity metrics were found to correlate with cancer recurrence.

**Conclusions:**

MOHA provides a simple and robust approach to characterize molecular and spatial heterogeneity of tissues. Research projects that generate spatially resolved tissue imaging data can take full advantage of this useful technique. The MOHA algorithm is implemented as a freely available R script (see supplementary information).

## Introduction

Tumor heterogeneity manifests itself in multiple ways in terms of observable features including tissue physiology, morphology, and histology, genotypes, gene expression, and protein expression [1,2,3,4,5]. The heterogeneity of these features can be studied at the inter-individual level [6,7] and at the intratumor level [8,9]. The inter-individual studies have typically relied on cell averaged, bulk tumor tissue measures. However, a full system-level characterization of tumor tissue heterogeneity is challenging and requires measures at the single cell level of a tissue.

Approaches to measure intratumor heterogeneity at the genomic level include computing allele fractions of the detected mutations from bulk tissue samples [10,11,12,13] or sequencing single cells [14,15]. A compromise between bulk tumor and single cell analysis is the isolation of smaller cell subpopulations by collecting samples from multiple tumor tissue regions or separating different types of cells into discrete tumor subsets by fluorescence-activated cell sorting [16,17]. The shortcoming of these approaches is that the in vivo cell spatial orientations, cell-cell interactions, and cell spatial heterogeneity remain unknown.

Digital pathology offers cell level details of molecular characteristics together with their spatial distribution. Multiplexed immunofluorescence (MxIF) tissue imaging can now measure the spatial concentration distribution of 60+ proteins on the same tissue [18,19,20,21]. The idea that both the cell types and their spatial distributions are biologically relevant is not contested, yet the methods to jointly characterize the heterogeneity of these in tissues are limited and still being established [22,23,24,25]. Efforts have been made toward spatially mapping the tumor microenvironment and the location of the immune cells relative to the tumor [26,27,28,29]. A proliferative heterogeneity analysis involved hexagonal tiling on whole-slide digital images of breast tumor tissues was conducted to characterize the spatial distribution of Ki67 expression [30,31]. The computed entropy metric was found to be an independent prognostic indicator of overall survival in breast cancer patients. In another study, heterogeneity was assessed in a tissue microarray constructed by sampling multiple foci of breast carcinomas. The heterogeneity of the immunomarker expression was computed by comparing within subject variances to the overall variance for the biomarkers. Intratumor heterogeneity was confirmed for five of the seven markers, while the authors raise the issue of the problematic extrapolation of these findings from small biopsy specimens to the entire tumor [32]. Zhong and colleagues designed a high-throughput image-based computational workflow to quantitate and visualize FISH-based copy number alterations in spatial context [33]. Although it provides an intuitive visual map of spatial heterogeneity of genomic level alterations, this approach is restricted to evaluating one or two genes at a time and does not consider tissue morphology.

**Fig 1:**
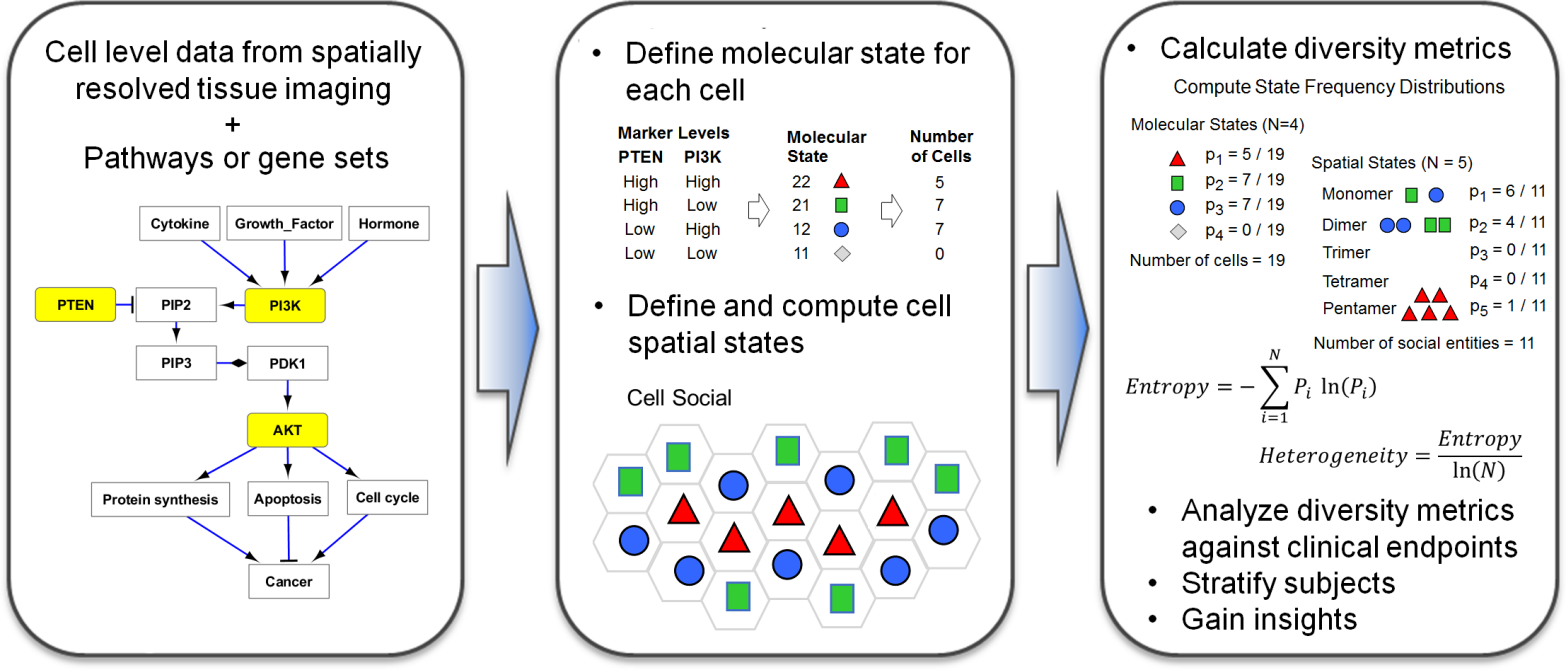
Conceptual overview of the MOHA method.

To advance the field of tumor heterogeneity characterization, we have developed the MOHA tool and method. The flow of information in the MOHA method is illustrated in Fig 1. The method combines single cell molecular measures from a tissue with pre-existing knowledge of biological pathways to assign states to cells in the tissue. It then incorporates positional measures of the cells to compute spatial state distributions. Tissue heterogeneity and diversity metrics are then computed from the observed distributions of these molecular and spatially defined states. Finally, these diversity metrics of the tissue are analyzed to gain biological insights. To demonstrate our MOHA method, we use MxIF imaging of tumor tissues from a colorectal cancer cohort. We show how the computed MOHA heterogeneity metrics correlate with cancer stage, histological tumor grade, and cancer recurrence.

## Materials and methods

### Colorectal cancer cohort dataset

Tissue samples from colorectal cancer (CRC) patients were collected at the Clearview Cancer Institute of Huntsville, Alabama, and provided to GE Global Research by Clarient Inc. The de-identified samples were acquired per institutional guidelines. This tissue microarray imaging cohort consisted of 747 paraffin-embedded patient tumor core samples distributed across three slides. These samples underwent multiplexed immunofluorescence microscopy and the results and experimental details have been reported previously [21]. Upon processing the tissue imaging data, quality filtering steps reduced the number of CRC cohort subjects (i.e. tumor core samples) from 747 down to 692. Clinical information was provided for each subject, including the histological tumor grade, cancer stage, gender, age, chemotherapy treatment (yes/no), and follow-up monitoring of 10 years (medium follow-up of 4.1 years across patients). Tables with breakdown of samples by histological tumor grade, cancer stage, and cancer reoccurrence events during follow-up can be found in section 1 of S1 File.

### MxIF tissue imaging data workflow

A detailed description of the multiplexed microscopy technique as well as the single-cell analysis and visualization methodology of biological features can be found elsewhere [21]. The minimum input data required by the MOHA algorithm is a plain tab-separated text file with one line per cell, specifying the following parameters: spatial (x, y) coordinates of the cell centroid, cell area, and the cell’s biomarker ordinal values. The workflow steps required to obtain the MOHA input data from MxIF tissue imaging data are as follows.

#### Step 1) Segment cell objects and generate biomarker measures from tissue images

MxIF imaging data typically comprises multiple tissue samples that have been imaged at 20x magnification. One image captures an entire tumor core sample from a tissue microarray. Each image undergoes quality filtering followed by cell segmentation to generate biomarker measures (mean and median value) for each cell and sub-cellular location (cytosol, nuclear, membrane). DAPI staining is used to define the nuclear area. The plasma membrane is segmented using a combination of staining patterns corresponding to the membrane proteins Na+/K+-ATPase and pan-cadherin. Cells are assigned x and y coordinates of centroid location. A cell type label is computed for each cell to be within or outside a computed epithelial region mask. The epithelial region mask is generated from the staining pattern produced by pan-cytokeratin and/or E-cadherin antibodies. Only cells located within the epithelial region mask were used for this study.

#### Step 2) Filter segmented cell objects that do not meet morphological quality criteria

Segmenting a million cells from images can produce some artifacts. To prevent these artifact objects from being included in the analysis, morphological quality filters are applied. The quality filters that were applied in the CRC study required cell objects to have one or two nuclei, a minimum (1.4 um^2^) and maximum (140 um^2^) area for both the cell nuclear and cytosol area. Cell objects that were on the edge of each image (~2 microns) were removed from the analysis. Images of tumor samples with less than 100 cells that fulfilled all filtering criteria were removed from further analysis.

#### Step 3) Filter biomarker measures that do not meet quality staining round metrics

A biomarker measure for a cell was removed if the cell’s quality round metric was below 0.8. The tissues underwent multiple rounds of staining, bleaching, and imaging which can lead to the deterioration of the tissue or other imaging artifacts. The quality round metric ranges from unity (perfect quality) to zero (total loss), and it is derived by computing the correlation of the DAPI stain intensity for the segmented cell portion of the image at a given round of staining to the baseline DAPI staining.

#### Step 4) Convert biomarker measures into ordinal values based on a n-state threshold model

The immunofluorescent intensity values for each biomarker (i.e. channel or staining round) integrated within each segmented cell and sub-cellular location (e.g. whole cell, cytosol, nuclear, membrane) were converted into ordinal values. For the CRC dataset, we selected a three-state threshold model, consisting of two threshold values established to bin the biomarker intensities into high, medium, and low states. The two threshold values were defined as the 33rd and 67th percentile of the sorted immunofluorescent intensities across the entire study. More biologically relevant threshold values could be established with control samples included in the multiplexed datasets to define normal and pathologically low or high values. In the absence of such controls, we split the data into comparable size bins. Threshold values were defined for each biomarker and for each sub-cellular location.

### Molecular and spatial states of cells

We selected the cells of the tissue as the atomic unit to compute diversity metrics on. Multiple diversity metrics were computed, as detailed in the next two sections. If the metric only contains information on the proportions of cells in different molecular states, then it is designated as molecular entropy or molecular heterogeneity. If additional information is incorporated about the spatial distribution of the cell states relative to each other, the metric is then designated as spatial entropy or spatial heterogeneity. The spatial metrics can again be of multiple types, depending on how the spatial state information is defined.

**Fig 2:**
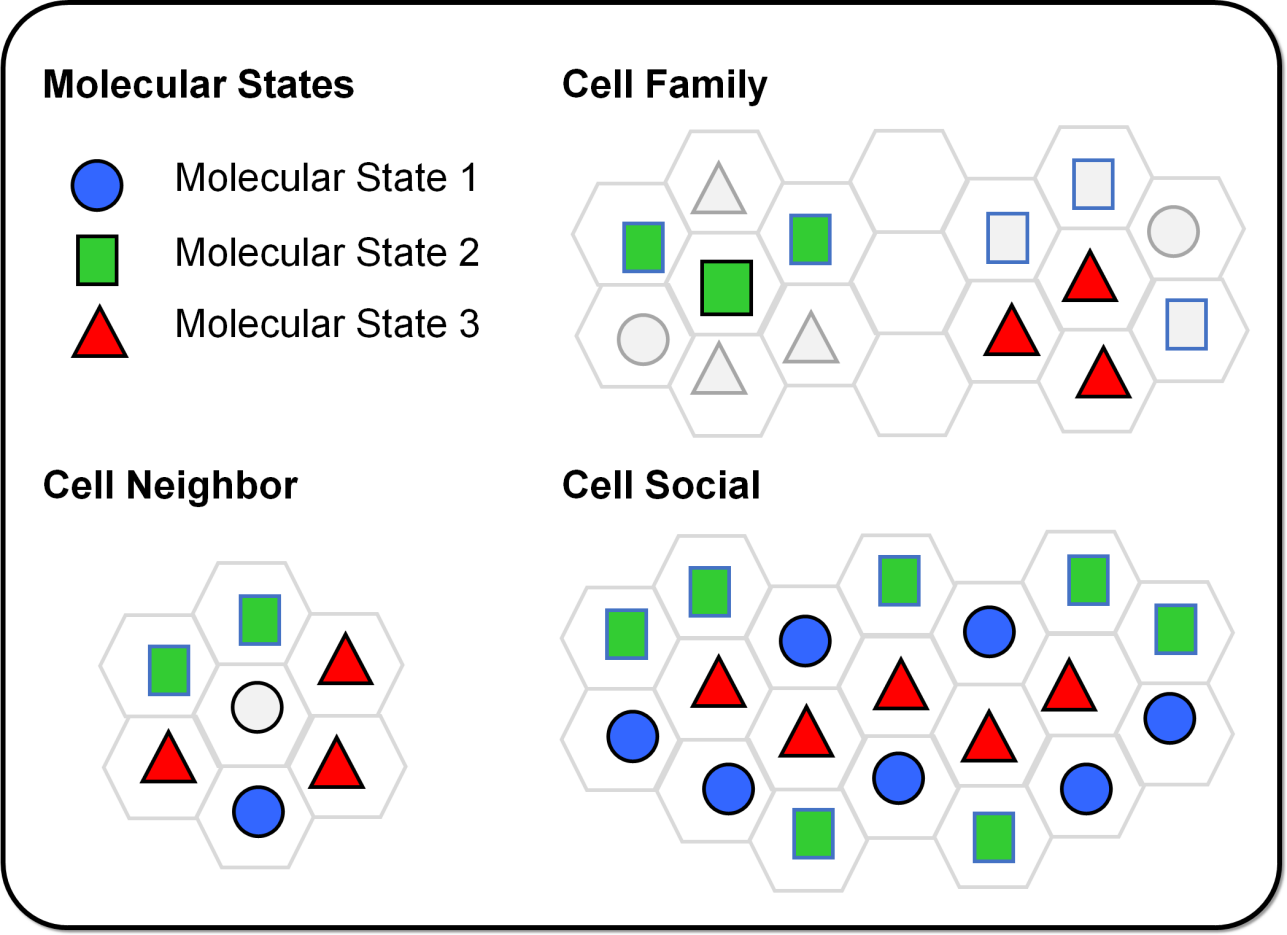
Conceptual examples of molecular and spatial states. In these examples, there are three unique molecular states represented by a circle, a square, and a triangle. The cell family metric is defined by the number of surrounding cells expressing the same molecular state as the central cell examined. The cell neighbor metric captures the clusters of neighboring cells in different molecular states. The cell social metric characterizes the diversity in the sizes of cell social clusters formed by a group of touching cells of the same state.

Cartoon examples of molecular and spatial states (i.e. species) are presented in Fig 2. In this conceptional view, cells can express three unique molecular states. The cell family metric is defined by the number of surrounding cells expressing the same molecular state as the one examined (thick black border). Both cells evaluated under Cell Family have a group size or spatial state of two (i.e. 2 dimers). The cell neighbor metric characterizes the diversity in the number of neighbors of different molecular states that surround the cell examined. The central cell illustrated for the Cell Neighbor in Fig 2 has three associated cell neighbor states: a monomer, a dimer, and a trimer. The cell social metric captures the diversity in the sizes of cell social groups. The example of 19 cells presents three unique cell social spatial states: 6 monomers, 4 dimers, and 1 pentamer.

### Molecular state diversity metrics

#### Selecting a pathway or gene set to define a cell’s molecular state

The selection of pathways or gene sets is limited by the number and type of immunofluorescence measurements available in the study. Using the nomenclature that pathways are networks represented by nodes and edges, the number of “measurable nodes” in a pathway reflect how well the available biomarker data will represent it. Well-designed imaging studies typically select biomarkers representing key driver genes (i.e. pathway nodes) of the biological process or disease under study. The AKT signaling pathway map centered on Protein Kinase B (also known as AKT) with links to cell apoptosis, cell cycle, protein synthesis, and cancer processes was selected to demonstrate the MOHA methodology. This pathway is known to be relevant for cancer and many of its nodes were quantified in the dataset presented here. Any other relevant pathway or gene set with multiple measured nodes could be used. Gene sets representing the hallmarks of cancer [34,35] were also selected. The AKT pathway and cancer hallmark gene sets used in this study are described in section 1 in S1 File (Figure A, Tables A and B).

#### Computing a molecular state for each cell using a pathway or gene set

The state value of an entire pathway or gene set was defined as a concatenation of the state values for each individual measurable node in the pathway assembled in a specific order. Connectivity information provided in pathway maps are not directly used for computing the diversity metrics, a current limitation of the MOHA tool. Therefore, the specific order of the genes in the pathway state concatenation sequence is arbitrary. However, once a sequence order has been chosen, it must be maintained consistently throughout the study. For example, the version of the AKT pathway we used for the colon cancer dataset had 16 measurable nodes (Figure A in S1 File). Some of these measurable nodes represented a phosphorylated state of the protein (e.g. SER-9 of GSK3B) and required specific antibodies to detect and quantify them. Other measurable nodes were proteins restricted to specific subcellular compartments. For example, there were two nodes in the AKT pathway for the protein CTNNB1, restricted to either the cytosol or nuclear subcellular compartment. These two nodes are represented using the immunofluorescence measurements that were integrated within their respective subcellular regions of the cell. For a three-state threshold model, each measurable node can have a high, medium, or low state encoded with a 0, 1, or 2 ordinal value. Therefore, a possible state for the AKT pathway is 2122202222211222 and would be assigned to those cells with biomarker measures (i.e. ordinal state values) representing this 16-measurable node concatenated sequence.

#### Molecular entropy and heterogeneity diversity metrics

The Shannon diversity index, also called Shannon entropy, was used to characterize the diversity of the various molecular and spatial state distributions [36,37]. There are alternative mathematical formulations of diversity with some modifying the sensitivity of the computed diversity value for rare or abundant states. Without any rational reason or biological observation to select one vs. another, we decided to use the original Shannon index. In the context of molecular diversity, the Shannon diversity index is a measure of how evenly the cells of the tissues are distributed among the possible molecular states that those cells exhibit. The entropy is maximized when all possible states are observed with the same frequency and is minimized when all cells are in the same molecular state. The molecular entropy is calculated as:

$$Molecular\;Entropy=-\sum_{i=1}^{Nm}{Pm}_{i}\;\ln\left({Pm}_{i}\right)$$

The Pmi is the fraction of cells in molecular state i, and Nm is the number of possible molecular states in the system. The maximum number of molecular states is defined by the maximum number of pathway states. This was computed based on the number of measurable nodes in the pathway and the number of node levels defined by the n-state threshold model. For our version of the AKT pathway with 16 measurable nodes, each having three levels, the maximum number of possible pathway states was 3^16, a little over 43 million. When the number of cells in the sample examined is smaller than the number of possible pathway states, the former is used as the maximum number of possible molecular pathway states.

There is no theoretical upper bound for the entropy value. For the sake of comparability of samples, it is sometimes convenient to use the normalized metric of heterogeneity, defined as the entropy divided by the natural log of the number of possible states. Heterogeneity values range from zero to unity.

$$Molecular\;Heterogeneity=\frac{Molecular\;Entropy}{ln(Nm)}$$

#### Molecular disparity metric

The molecular entropy and heterogeneity metrics describe the molecular complexity of the system. Each molecular state is treated as a distinct specie. When defining a molecular pathway state based on the individual states of the measurable nodes of the pathway, there are some pathway states that are more similar than others. If two pathway states differ only in a single measureable node level, the molecular distance between the two pathway states is small. However, if every node has a different value, the distance between the two pathway states will be larger. Borrowing from the concepts of complexity and disparity in multi agent systems [38,39], we define a molecular disparity metric for a sample with the maximum number of molecular states Nm as:

$$Molecular\;Disparity=\sum_{i=1}^{Nm-1}\sum_{j=i+1}^{Nm}{Pm}_{i}{Pm}_{j}\;{d(i,j)}^{2}$$

Pmi and Pmj are the fractions of cells in molecular states i and j, and d(i,j) is the molecular distance between states i and j. This distance is computed as the sum of differences across the measurable nodes:

$${d(i,j)}^{2}=\sum_{n=1}^{Npn}{\left(M_{n,i}{-M}_{n,j}\right)}^{2}$$

where Mn,i and Mn,j are the values assigned to pathway node n in pathway states i and j, and Npn is the number of measurable pathway nodes.

### Spatial state diversity metrics

#### Defining cell neighbors for spatial metrics

Identifying neighboring cells is necessary for computing the spatial diversity metrics. We have used two different approaches to achieve this: an exact pixel-based method and a faster approximate method. Using the segmented tissue images, it is possible to represent the edge of each cell by a set of pixel points. When deciding if two cells are spatially first neighbors (i.e. touching cells), the edge pixel points from the two cells can be compared seeking for the condition in which the distance between an edge pixel point from one cell is within one-pixel distance of an edge pixel point from another cell. This comparison of pixel points is considered the exact method. Alternatively, an approximate method was implemented to be computationally twice as fast and not require repeated image processing. The cells were approximated by circles, and the distance between their centers had to be smaller than a critical parameter multiplied by the sum of their radii. This was defined as:

$$\sqrt{\frac{{\left(x_{i}-x_{j}\right)}^{2}+{\left(y_{i}-y_{j}\right)}^{2}}{{\left(r_{i}+r_{j}\right)}^{2}}}\;\leq d_{critical}\;where\;r_{i}=\sqrt{\frac{A_{i}}{\pi }}$$

where the Euclidean distance between the centers of two cells (xi, yi) and (xj, yj) is computed and normalized by the sum of the approximate radii of the two cells (ri and rj). The cell radii were computed from the segmented area of the cells (Ai, Aj), approximating the cells on the 2D images as circles. If this normalized Euclidean distance is equal to or less than the dimensionless critical parameter, d_critical_, the cells i and j are the classified as touching neighbors.

To establish the value of the dimensionless critical parameter, d_critical_, the approximate method was compared to the exact method for over 752 million unique cell pairs from the colorectal cancer data set. The change in the number of correctly and falsely identified touching cell neighbors as a function of the critical parameter was computed. A critical parameter of 1.31 minimized the number of false predictions, resulting in the best agreement between the approximate and exact methods. This resulted in the approximate method having a positive predictive value of 0.884 and the negative predictive value of 0.997. This means that when the approximate method indicated that two cells are touching, there was an 88.4% probability that the two cells in the image were touching each other. The spatial diversity metrics for the AKT pathway were calculated using both the exact and the approximate method. The diversity metrics computed by the approximate method correlated with those computed by the exact method with correlation coefficients that ranged from 0.98 to 0.998. Plots of these correlations are presented in section 2 in S1 File.

#### Cell coordination number diversity metric

The coordination number of a cell represents the number of cells surrounding and touching it (i.e. neighbors), as defined in the previous section. In a regular two-dimensional grid arrangement (i.e. lattice) of cells, the coordination number for each cell is the same, except for those at the edge of the lattice. This is not the case for a biological tissue where the coordination numbers differ from one cell to the other. A tissue will have a characteristic frequency distribution of cell coordination numbers. An entropy metric for the cell coordination number distribution can be computed using the Shannon diversity index. The cell coordination number entropy metric did not include any molecular state information, and can therefore be considered a pure spatial diversity metric. Alternatively, the molecular states of the cells and their immediate neighbors can be used to define various diversity metrics that include molecular information in addition to spatial context. Three spatial diversity metrics are presented below: Cell Family, Cell Neighbor, and Cell Social. The entropy values for these three-spatial metrics were computed using the Shannon diversity index. The difference between them comes from the definition of the individual spatial states and the maximum number of possible states.

#### Cell family diversity metric

The cell family state metric was computed by surveying the neighbors of each cell and counting only the number of neighbors in the same molecular state. This number of neighbors represents the cell family state. Having no neighbors in the same molecular state is a valid cell family state. Therefore, the cell family state can range from zero to the maximum number of neighbors a cell has. After going through every cell and their touching neighbors, a frequency distribution was established for these cell family states. The cell family entropy was then computed as:

$$Cell\;Family\;Entropy=-\sum_{k=0}^{Z_{max}}{Ps}_{k\;}ln({Ps}_{k})$$

where, Psk is the frequency of state k, and Zmax is the maximum number of cell family states. For this diversity metric, Zmax equals the maximum number of neighbors a cell might have in the tissue image, which is the same as the maximum coordination number. The cell family heterogeneity was computed by dividing its entropy by the natural log of Zmax + 1.

#### Cell neighbor diversity metric

The cell neighbor spatial metric characterizes the diversity in the molecular states of a cell’s neighborhood. Whereas the cell family metric gives rise to a single state for each cell (number of same molecular state neighbors), the cell neighbor metric defines as many states around each cell as the number of different molecular states that are present in its neighborhood. For example, in Fig 2, Cell Neighbor has a central cell surrounded by cells in three different states, resulting in three cell neighbor states of 1, 2 and 3 shown as one circle, two squares and three triangles.

#### Cell social diversity metric

The cell social spatial metric characterizes the diversity in the sizes of cell social groups. Each grou;p is composed of cells that express the same molecular state and are spatially linked. The social group size is the number of cells in the group. Each cell in the group must touch at least one other cell in the group. The group of cells may be spread out or clumped together (Fig 2). After assigning each cell to a social group by the molecular and spatial constraints just described, the cell social frequency distribution can be computed. As before, the Shannon index was used to compute the entropy from the frequency distribution. The cell social heterogeneity was obtained by dividing the cell social entropy by the natural log of the maximum number of states.

The maximum number of cell social states, Ns, that is theoretically possible is dependent upon the total number of cells, Nc, present in the system. Each cell social state is a group of cells of a unique number. Summing over all possible cell social states will compute the minimum number of cells required to observe all those states. This is mathematically represented as:

$$\sum_{i}^{N_{S}}i=\frac{N_{S}(N_{S}+1)}{2}\;\leq \;N_{C}$$

Solving the inequality leads to the formula for computing the maximum number of possible cell social spatial states, Ns, for a system with Nc cells:

$$N_{S}=Int\left(\frac{\sqrt{8N_{C}+1}-1}{2}\right)$$

#### Random sampling method to decouple cell molecular states from cell locations

Knowing that cells communicate with each-other, it is reasonable to expect the spatial distribution of the molecular states among the cells of a tissue to be non-random. The spatial diversity metrics reflect this deviation from randomness. We applied two methods to assess the interaction between the cell molecular states and their relative spatial orientations. The first method was a random sampling method and the second was a probability based method. The random sampling method required generating randomized arrangements of the cell molecular states among the cell locations, followed by computing the spatial diversity metrics. This process was repeated 120 times for each sample to generate a distribution for each spatial metric.

#### Probability based method to compute cell family diversity metric

An alternative, probability based method was employed that computed an estimate of the mean of the spatial diversity metric upon randomizing the arrangements of the cell molecular states among the cell locations. The method computed all possible configurations based on the molecular distribution (Pm) for each cell and its respective cell coordination number. For a cell family group size of k, the number of configurations Ns_k_ is computed as:

$${Ns}_{k}^{\;}=\sum_{j=1}^{Nc}\sum_{i=1}^{Nm}{\left(1-{Pm}_{i}\right)}^{Z_{j}-k}\;{\left({Pm}_{i}\right)}^{k+1}\frac{Z_{j}!}{(Z_{j}-k)!k!}$$

Pm_i_ is the fractions of cells in molecular states i and Zj is the coordination number of cell j that is being evaluated. The frequency of occurrence, Ps_k_, for cell family state k, is obtained after normalization:

$${Ps}_{k}=\frac{{Ns}_{k}^{\;}}{\sum_{k=0}^{Z_{max}}{Ns}_{k}^{\;}}$$

With the cell family state frequency distribution, Ps_k_, defined, the cell family diversity is then simply computed from cell family entropy equation shown above.

The key parameters for computing the molecular and spatial diversity metrics is summarized in Table 1.

**Table 1:** Summary of key parameters used in computing the diversity metrics.

d(i,j)	molecular distance between states i and j
Mni, Mnj	values assigned to pathway node n in pathway states i and j
Nc	number of cells in the system
Nm	number of possible molecular states in the system
Npn	number of measurable nodes in a pathway or gene set
Ns	number of possible spatial states in the system
Nsk	number of spatial configurations for the group size k
Pmi, Pmj	fractions of cells in molecular states i and j
Psk	frequency of the spatial state of group size k
Zj	coordination number of cell j
Zmax	maximum coordination number in the system

## Results

### MOHA diversity metrics capture tissue cell molecular states and their spatial arrangements

**Fig 3:**
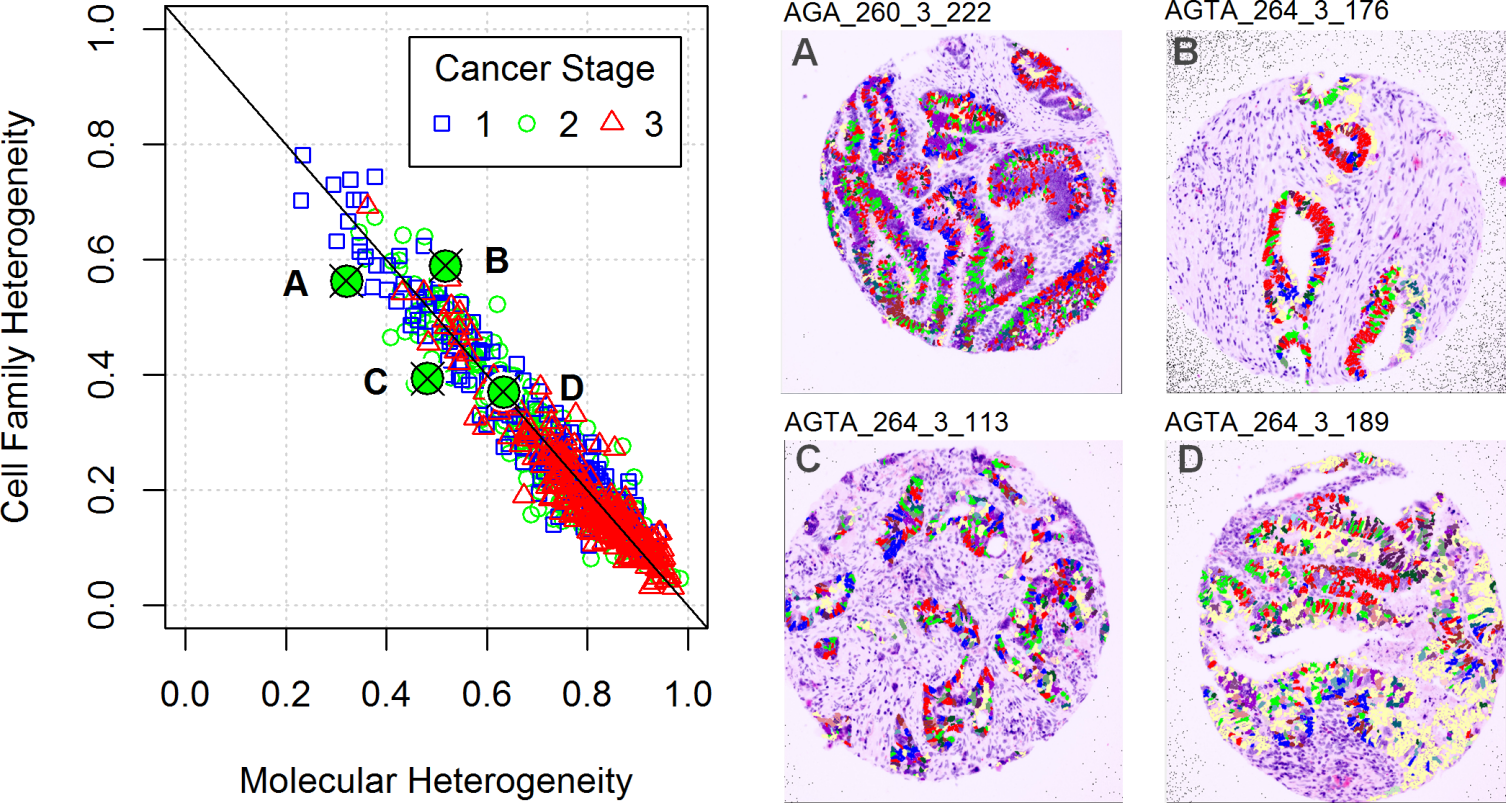
Molecular and spatial diversity paired with virtual H&E images of four samples. The scatter plot presents the heterogeneity values for 691 CRC subjects. The values corresponding to four CRC stage 2 subjects with histological grade 2 tumors are highlighted. Corresponding tissue images of these four examples are shown in panels A-D. These virtual H&E stained images are overlaid with segmented cells that are colored based on the molecular state that the cells express. These tissue images are illustrative examples of cases when molecular heterogeneity values are the same, but the family metrics of spatial heterogeneity are different (B and C) pointing to differences in spatial configuration of the two tissue samples. Similarly, there are cases where the family heterogeneity metrics are similar, but the molecular metrics differ (A versus B or C versus D). Despite the significant correlation between the molecular and spatial metrics, they have the potential to capture and reflect different properties of the tumor tissues.

We first computed the diversity metrics for each tumor core sample from the CRC dataset and then compared these computed metrics with the tissue images. Four tissue image examples are presented in Fig 3 (labeled A-D) along with a plot of their molecular and cell family heterogeneity metric values in context of the entire CRC cohort. Although the molecular and spatial diversity values showed a significant correlation, there were samples, such as B and C, that displayed rather different spatial diversity despite their almost identical molecular heterogeneity and vice-versa (A and B, or C and D). There was a general trend of increasing molecular heterogeneity and decreasing cell family heterogeneity with higher cancer stage.

**Fig 4:**
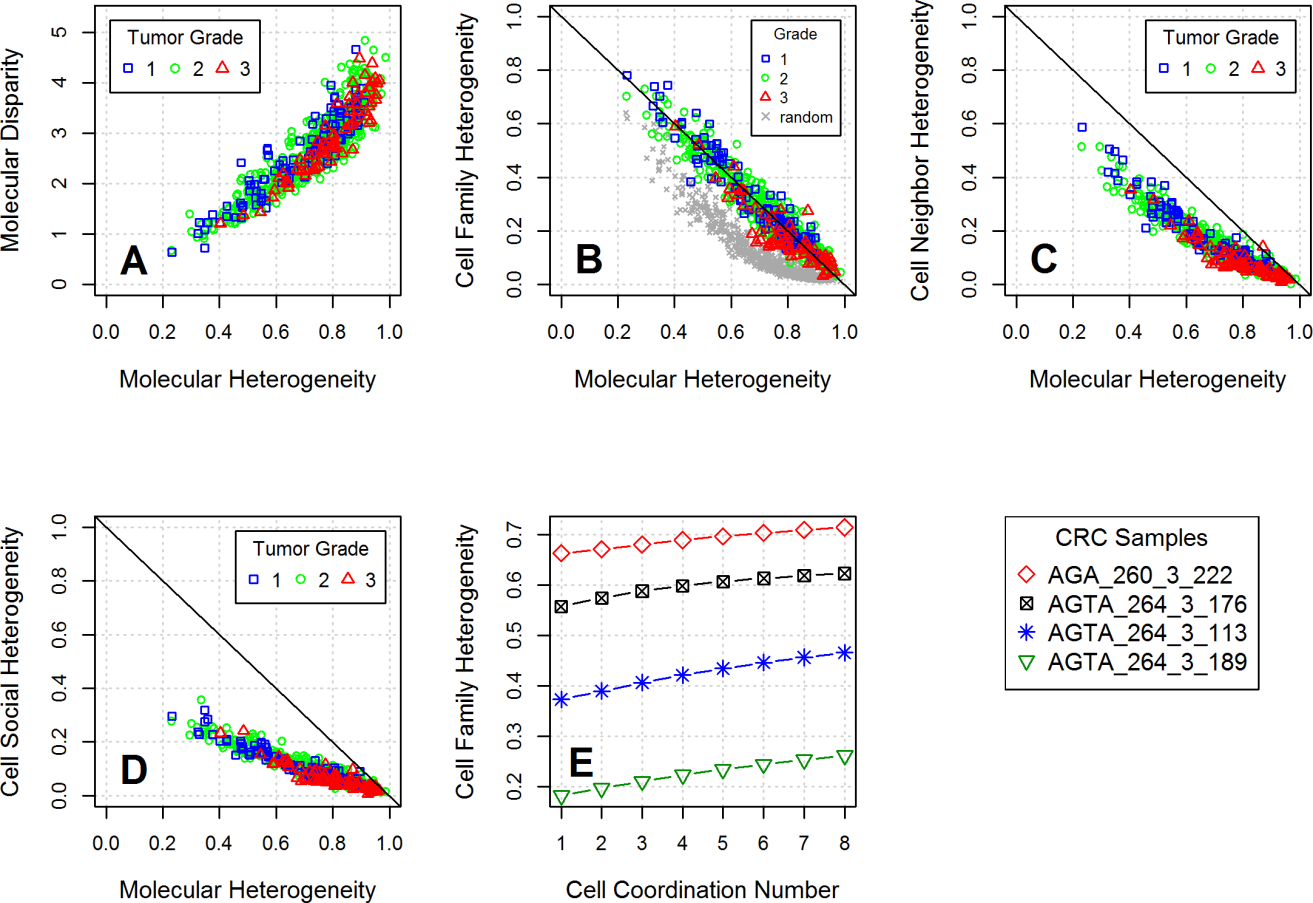
Molecular and spatial diversity metrics calculated for the CRC dataset based on the AKT pathway. (A) The molecular disparity is directly correlated with the molecular heterogeneity, but it tends to decrease with increasing tumor grade (1–3) at a given level of molecular heterogeneity or complexity. (B-D) The spatial metrics are inversely correlated with the molecular metrics. The molecular heterogeneity tends to increase with tumor stage and grade, while the spatial metrics tends to show the opposite trend. (E) Both molecular state distribution and spatial arrangement (reflected by the cell coordination number) contribute to the cell family heterogeneity, with the molecular state distribution having a larger effect.

Similar trends were observed between the molecular and the cell neighbor and cell social spatial diversity metrics (Fig 4C and 4D). Both the cell neighbor and the cell social heterogeneity inversely correlated with the molecular heterogeneity, but their values covered a smaller range than the cell family metric. The molecular disparity correlated highly with the molecular heterogeneity (Fig 4A), indicating that the distances between the pathway states of the cells had a similar distribution across the samples examined, with increasing disparity and complexity as tumor grade increases.

The high correlation between the molecular and spatial diversity metrics indicates that the molecular states of the cells are the major source of diversity. To probe how much additional information the topology of the tissue can add to the spatial diversity metric, two decoupling approaches were used. First, the spatial arrangement of the cells was randomized, while keeping the molecular state profiles the same. The average cell family versus the molecular heterogeneity values for these “decoupled” synthetic cases with random cell arrangements are shown with grey cross symbols in Fig 4B. The difference between the random and real values is an indication that the arrangement of cells in real tissues (tissue topology) relative to their molecular states is indeed not random.

For the second approach, we took the molecular pathways state distributions of the four samples shown in Fig 3A–3D), and computed the cell family heterogeneity metrics for model tissues with mean coordination numbers ranging from 1 to 8 using a probability based method described in detail in the Methods section. The results shown in Fig 4E reveal that the molecular state distribution taken from four different samples significantly influenced the absolute value of cell family heterogeneity metric. The cell coordination numbers, which reflect the topology of the tissue, had a smaller influence. Fig 4E illustrates that increasing coordination numbers result in higher cell family heterogeneity values.

### Diversity metrics correlate with cancer stage and tumor grade

**Fig 5:**
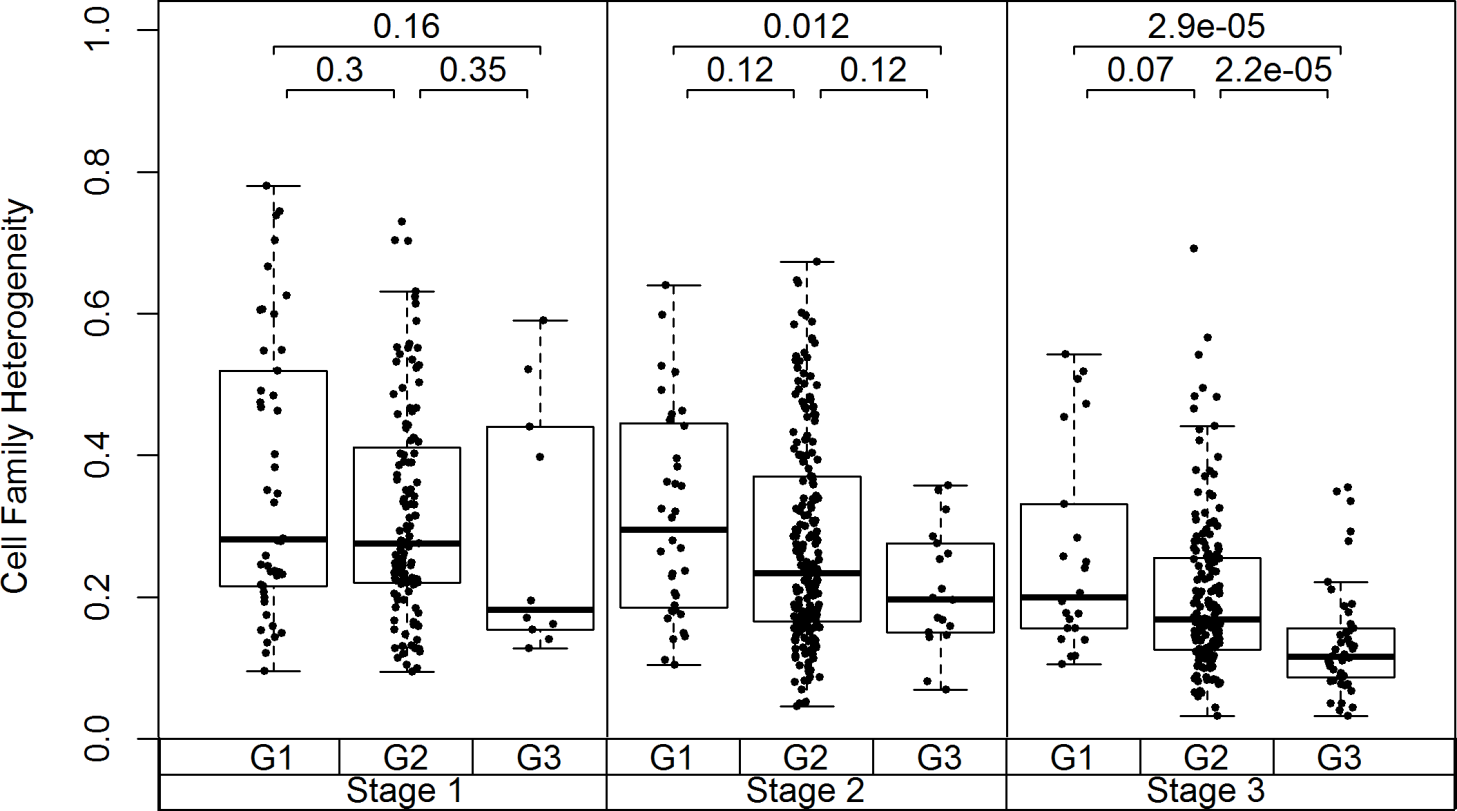
The cell family heterogeneity metrics computed based on the AKT pathway for the CRC dataset samples showed a decreasing trend with increasing cancer stage (1 to 3) and tumor grade (G1 to G3). The Mann-Whitney test p-values of the comparisons of tumor grades within each cancer stage are shown.

To assess if our diversity metrics have captured relevant biology, we performed a correlation analysis between the diversity metrics and the clinical measures of cancer stage and histological tumor grade. Utilizing the CRC cohort dataset, we computed the Spearman's rank correlation between the diversity metrics and the subject’s cancer stage or tumor grade measures. A highlight of the results is presented in Table 2 and Fig 5 for the AKT pathway diversity metrics with cancer stage and tumor grade for 670 subjects. Refer to worksheet A and B in S2 File and section 3 in S1 File for a complete set of correlation results and plots that include all the cancer hallmark gene sets. We observed strong and statistically significant correlations (p-values < 1E-5) between the molecular and spatial diversity metrics for both cancer stage and tumor grade. Overall, the correlations were stronger for cancer stage than for tumor grade. As noted before, the molecular heterogeneity was found to increase with cancer stage and tumor grade, while the spatial heterogeneity metrics showed the opposite trend (Fig 4B–4D). This was found to be the case for each of the cancer hallmark gene sets (see sections 4 and 5 in S1 File).

**Table 2:** Spearman's rank correlation of diversity metrics with cancer stage and tumor grade for the AKT pathway.

AKT Pathway	Cancer Stage		Tumor Grade	
Metric	r	p-value	r	p-value
Molecular Heterogeneity	0.34	7.5E-20	0.19	1.4E-06
Molecular Disparity	0.33	2.4E-18	0.11	6.2E-03
Cell Family Heterogeneity	-0.37	1.1E-23	-0.25	2.1E-11
Cell Neighbor Heterogeneity	-0.37	1.5E-23	-0.25	8.6E-11
Cell Social Heterogeneity	-0.39	4.0E-25	-0.25	7.4E-11
Cell Coordination Number Entropy	-0.12	2.1E-03	-0.11	4.4E-03
Cell Family Het. (Probability Based)	-0.33	1.1E-18	-0.18	1.8E-06

### Diversity metrics correlate with cancer recurrence

**Fig 6:**
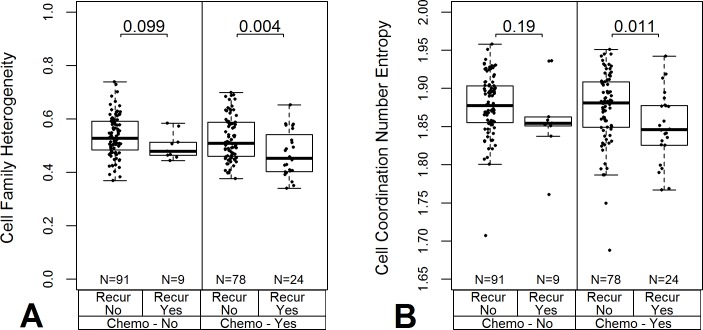
Cell family heterogeneity of Inducing Angiogenesis cancer hallmark and its variation between cancer recurrence groups of stage 2 cancer subjects with histological grade 2 tumor tissues. (A) The median heterogeneity was lower for subjects that had a cancer recurrence event during the follow-up monitoring (Recur Yes) vs. those that did not regardless of chemotherapy (Mann-Whitney test p-values 0.099 and 0.004). (B) The entropy of the cell coordination number distribution decreased for those subjects with a recurrence event.

The Spearman's rank correlation between several diversity metrics and cancer recurrence for the CRC cohort subjects who received chemotherapy are shown in Table 3. The table presents the Spearman's rank correlation of diversity metrics with a cancer recurrence event during follow-up for 338 subjects that received chemotherapy and subset of 102 cancer stage 2 subjects with histological grade 2 tumor tissues. The correlation of stage and grade with recurrence was computed using multiple linear regression. Worksheet C in S2 File provides a comprehensive overview of correlations. The cell family heterogeneity metric computed based on the cancer hallmark Inducing Angiogenesis had the highest correlation with cancer recurrence. Interestingly, this diversity metric did not correlate as strongly to cancer stage (r = -0.25) as the AKT pathway metric (r = -0.37). The mean cell family heterogeneity was found to be lower for those with a recurrence event (Fig 6A).

**Table 3:** Spearman's rank correlation of diversity metrics with cancer stage and tumor grade for the cancer hallmark gene set, Inducing Angiogenesis.

Chemotherapy Treatment Group Inducing Angiogenesis	Recurrent Event		Recurrent Event Stage 2 Grade 2	
Metric	r	p-value	r	p-value
Stage and Grade	0.18	5.2E-03	NA	NA
Molecular Heterogeneity	0.16	4.1E-03	0.15	0.144
Molecular Disparity	0.17	2.2E-03	0.16	0.103
Cell Family Heterogeneity	-0.17	2.2E-03	-0.29	3.5E-03
Cell Coordination Number Entropy	0.00	0.94	-0.25	0.010
Cell Family Het. (Probability Based)	-0.16	2.5E-03	-0.27	6.9E-03

We observed trends in subsets of subjects based on stage and/or grade that are missing when examining the entire cohort. The Spearman's rank correlations for all subjects who underwent chemotherapy and the stage 2 grade 2 subset are presented in Table 3. The molecular diversity indices show approximately the same correlations with recurrence (~0.16) for the entire chemo-treated group and those limited to stage 2 tumor grade 2 cases. In contrast, for the cell family heterogeneity, the correlation improved (-0.17 to -0.29). Although we have highlighted the cell family heterogeneity metric computed for the Inducing Angiogenesis cancer hallmark, the same trends are observed with less statistical significance for other spatial metrics and cancer hallmarks (section 6 in S1 File). The Cell Coordination Number Entropy showed the same trend (Fig 6B). This spatial metric correlation of -0.25 for the stage 2 grade 2 cases is zero for the chemo treated group that includes all cancer stages and tumor grades (Table 3). These results suggest that within the stage 2 grade 2 cases, there are spatial features differentiating the subjects in terms of cancer recurrence.

### MOHA tool implementation

The MOHA algorithm has been implemented in R. The freely available R scripts include the capability to compute the diversity metrics on the CRC dataset presented here. In addition, functions have been included to generate plots and data tables. The README document from the package contains a step-by-step guide to running the R scripts.

## Discussion

The main goal of our work was to develop an approach for the quantitative characterization of tumor heterogeneity from spatially resolved molecular measures of cells. Although we utilized a MxIF tissue imaging dataset to demonstrate our method, the MOHA algorithm can be applied to any dataset that provides spatially resolved molecular measures. We strived to maintain some level of physical and biological rationale for the diversity metrics and to enable intuitive customization of the metrics to best address the biological questions asked. Consequently, the molecular metrics focused on the cell as the atomic unit of the tissue. The molecular state of the cell was defined based on signaling pathways and biological based gene sets depending on their relevance for the specific problem (i.e. the biological question) being examined. The underlying physiological phenomenon behind the use of touching cell neighbors for computing the spatial metrics can be primarily attributed to adjacent cell communication through juxtacrine signaling. Two other mechanisms of cell communication include synaptic or paracrine signaling. The distance at which paracrine signaling typically occurs in vivo is uncertain but is much more likely to effectively occur between two cells in in close spatial proximity of each other. Our methodology is general enough to emphasis paracrine signaling over juxtacrine signaling by changing the dimensionless critical parameter in our approximate method to determine if two cells are neighbors. We tuned the critical parameter to identify cells that were in direct contact with each other based on tissue imaging data. However, this parameter could be increased to identify cells as neighbors over greater spatial distances to model paracrine signaling occurring over longer distances. The cell coordination number reflects tissue topology including epithelial cell polarization, basal luminal organization of glandular structures, and the epithelial-mesenchymal transition in cancer.

Despite their roots in intuitive biological and physical characteristics of the tissue samples, these metrics are statistical in nature. To be meaningful and representative, the metrics should be computed based on many cells. For this study, we used tissue microarrays with core diameters of approximately 0.7–0.8 μm and selected the empirical cutoff of 100 cells in a sample at minimum to pass the first quality filter. This was a realistic compromise to have a non-trivial number of cells without disqualifying too many samples.

The discriminative power of the cell family, cell neighbor, and cell social spatial metrics toward cancer stage and tumor grade can be attributed to the combined inclusion of molecular and spatial information from the tumor tissue. Being able to compute metrics based on each of these factors separately makes it possible to quantify their contributions independently. Similarly, the gene sets or pathways providing the greatest correlation to cancer stage and tumor grade can potentially indicate which biological process are driving the progression of the specific cancer type studied.

We examined the correlation of the diversity metrics with clinical characteristics for the entire cohort, and separately for stage 2 and grade 2 subjects. Especially at this intermediate stage, CRC is heterogeneous with multiple treatment options and various tumor responses that can lead to multiple outcomes. It is critically important to be able to stratify CRC patients at this intermediate stage to identify the more aggressive phenotype and enable the selection of a more aggressive therapy to improve long-term survival. Perineural invasion or the expression of certain non-coding RNA’s are promising prognostic biomarkers, but no final conclusion has been reached about their value [40,41,42]. Tumor heterogeneity metrics could provide additional prognostic factors computed from pathology tissue or biopsy samples. They could potentially provide additional discriminatory power in existing multivariant prognostic models to improve their sensitivity and specificity.

We have shown that both the molecular and the spatial diversity metrics correlate with tumor stage and grade across the entire cohort to varying degrees. The cell coordination number, a purely spatial metric, showed an interesting behavior (Figure C in S1 File). Its average value increased with stage in grade 1 tumors, then it stayed relatively unchanged for grade 2, followed by a decreasing trend with stage for grade 3 tumors. This observation led us to speculate about the ability of the cell coordination number to reflect the longitudinal changes in tumor spatial structure. Starting from the healthy gland, through a more compact structure with increasing number of cell neighbors, to a collapsed, irregular structure in which tumor cells become more isolated with fewer cell neighbors.

We have found the MOHA methodology to be generalizable to other types of cancer including prostate, breast, lung and brain. This is not to say that the tumor heterogeneity is the same across all these cancers but that the physical and biological rationale for our diversity metrics are. For example, the cell coordination number reflects the close spatial proximity of cells to each other that is critical to juxtacrine, paracrine, and synaptic signaling mechanisms. Consequentially, the MOHA spatial metrics are defined to imbed the underlying biological phenomenon (e.g. cell-cell communication) that is common across all tissues. The actual interpretation of the spatial metrics between healthy and the various stages of a disease will be tissue and disease specific (e.g. epithelial-mesenchymal transition in cancer). Our methodology is also general from the perspective of working with any gene sets or pathways to define the molecular state of cells. However, those that provide the greatest insights and discriminatory power will likely be specific to the biological processes driving the progression of the disease under study.

Beyond computing the diversity metrics across the entire sample, this methodology can be used to examine the clonal composition of the tumor. It enables the identification of subsets of cells and their relative locations within a tumor contributing to correlations with clinical metrics across cohorts. This is a direction worth exploring in the immediate future.

### Limitations of the MOHA metrics

There are no generally accepted methods available for characterizing tissue heterogeneity at the cell level, and the number of metrics one can propose could be very large. Testing all of them is neither feasible nor practical. Our metrics are based on the Shannon diversity index, a well-known statistical metric frequently used in other scientific areas. What makes these metrics tissue specific and potentially relevant is the choice of the state definitions. While we attempted to make them biologically intuitive, these definitions are exploratory in nature. We designed them in a way that makes it relatively straightforward to modify them. Further testing on multiple datasets and tissue types will enable us to judge how to select and modify them to maximize their usefulness.

Perhaps the most serious limitation of these heterogeneity metrics originates from the semi-quantitative nature of the immunofluorescence intensities used to characterize biomarker levels in the tissue. Ideally, every biomarker specific and fluorescently labeled antibody should have a set of standards allowing the user to calibrate the intensity measurements and relate them to true protein concentrations. In addition, every slide of tissue microarrays should have multiple normal controls to establish disease-relevant ranges for the biomarkers measured. Unfortunately, none of these are routinely available for existing clinical datasets. The methodology of computing the MOHA metrics will not change when such standards and controls become a reality, but their predictive power is expected to improve.

Although we selected measured nodes in the AKT pathway, The MOHA metrics include no information about the pathway connectivity or the directionality of the interactions. Ways to incorporate such information into the heterogeneity metrics is a direction worth exploring. Finally, it is worth mentioning that the spatial metrics proposed here can be somewhat tissue-dependent which limits the ability to draw general conclusions about the relationship between spatial heterogeneity and tumor progression across all cancers.

## Supporting information

S1 File Supplementary documents. This pdf file (3.3 MB) contains seven supplementary document sections of text and figures. Section 1 presents additional information on methods, the AKT pathway, gene sets and the CRC cohort. Section 2 presents the change in the number of correct and incorrect cell neighbor assignments (True Positives, False Positive, False Negatives) by comparing the assignment of the approximate method of computing cell neighbors relative to the exact method using the cell’s segmented image pixels. Assignments broken out by cancer stage and tumor grade. Figures showing the correlations between the diversity metrics as calculated with the exact method of cell neighbor identification versus the approximate method. Section 3 presents box charts with diversity metrics by cancer stage and tumor grade. Section 4 presents plots with molecular disparity, cell family, cell neighbor, and cell social heterogeneity versus molecular heterogeneity computed across 7 gene sets corresponding to cancer hallmarks and the AKT pathway. The values are colored by cancer stage. Section 5 presents plots with molecular disparity, cell family, cell neighbor, and cell social heterogeneity versus molecular heterogeneity computed across 7 gene sets corresponding to cancer hallmarks and the AKT pathway. The values are colored by cancer grade. Section 6 presents box charts with diversity metrics by chemotherapy treatment and recurrence calculated based on 7 gene sets corresponding to cancer hallmarks and the AKT pathway. Box charts of average cell coordination number, number of cells and age at diagnosis broken down by treatment and recurrence are also included. Section 7 presents the frequency distributions of cell coordination numbers and by cancer stage and tumor grade.
(PDF)

S2 File Supplementary tables. This excel file (0.2 MB) contains three worksheets. Worksheet A presents the correlation of diversity metrics with cancer stage. Worksheet B presents the correlation of diversity metrics with tumor grade. Worksheet C presents the correlation of diversity metrics with cancer recurrence.
(XLSX)

S3 File MOHA tool. This zip file (0.5 MB) contains an R script implementation of the MOHA tool and supporting data files to compute MOHA diversity metrics. The README document within this zip file contains instructions on running the R scripts.
(ZIP)
