# Supplementary material for: Characterizing the heterogeneity of tumor tissues from spatially resolved molecular measures
Source: PLoS One. 2017 Nov 30;12(11):e0188878. doi: 10.1371/journal.pone.0188878 (PMC5708750; doi:10.1371/journal.pone.0188878)
Supplement: S1 File — This pdf file (3.3 MB) contains seven supplementary document sections of text and figures. Section 1 presents additional information on methods, the AKT pathway, gene sets and the CRC cohort. Section 2 presents the change in the number of correct and incorrect cell neighbor assignments (True Positives, False Positive, False Negatives) by comparing the assignment of the approximate method of computing cell neighbors relative to the exact method using the cell’s segmented image pixels. Assignments broken out by cancer stage and tumor grade. Figures showing the correlations between the diversity metrics as calculated with the exact method of cell neighbor identification versus the approximate method. Section 3 presents box charts with diversity metrics by cancer stage and tumor grade. Section 4 presents plots with molecular disparity, cell family, cell neighbor, and cell social heterogeneity versus molecular heterogeneity computed across 7 gene sets corresponding to cancer hallmarks and the AKT pathway. The values are colored by cancer stage. Section 5 presents plots with molecular disparity, cell family, cell neighbor, and cell social heterogeneity versus molecular heterogeneity computed across 7 gene sets corresponding to cancer hallmarks and the AKT pathway. The values are colored by cancer grade. Section 6 presents box charts with diversity metrics by chemotherapy treatment and recurrence calculated based on 7 gene sets corresponding to cancer hallmarks and the AKT pathway. Box charts of average cell coordination number, number of cells and age at diagnosis broken down by treatment and recurrence are also included. Section 7 presents the frequency distributions of cell coordination numbers and by cancer stage and tumor grade. (PDF) [file pone.0188878.s001.pdf]

Index

Section 1
Additional information on methods, gene sets and patient cohort.

Section 2
Change in the number of correct and incorrect cell neighbor assignments (True Positives, False Positive, False Negatives) by comparing the assignment of the approximate method of computing cell neighbors relative to the exact method using the cell's segmented image pixels. Assignments broken out by cancer stage and tumor grade. Figures showing the correlations between the diversity metrics as calculated with the exact method of cell neighbor identification versus the approximate method.

Section 3
Box charts with diversity metrics by cancer stage and tumor grade

Section 4
Plots with molecular disparity, cell family, cell neighbor, and cell social heterogeneity versus molecular heterogeneity computed across 7 gene sets corresponding to cancer hallmarks and the AKT pathway. The values are colored by cancer stage.

Section 5
Plots with molecular disparity, cell family, cell neighbor, and cell social heterogeneity versus molecular heterogeneity computed across 7 gene sets corresponding to cancer hallmarks and the AKT pathway. The values are colored by cancer grade.

Section 6
Box charts with diversity metrics by chemotherapy treatment and recurrence calculated based on 7 gene sets corresponding to cancer hallmarks and the AKT pathway. Box charts of average cell coordination number, number of cells and age at diagnosis broken down by treatment and recurrence are also included.

Section 7
Frequency distributions of cell coordination numbers and by cancer stage and tumor grade.

Section 1

1. Pathways and gene sets

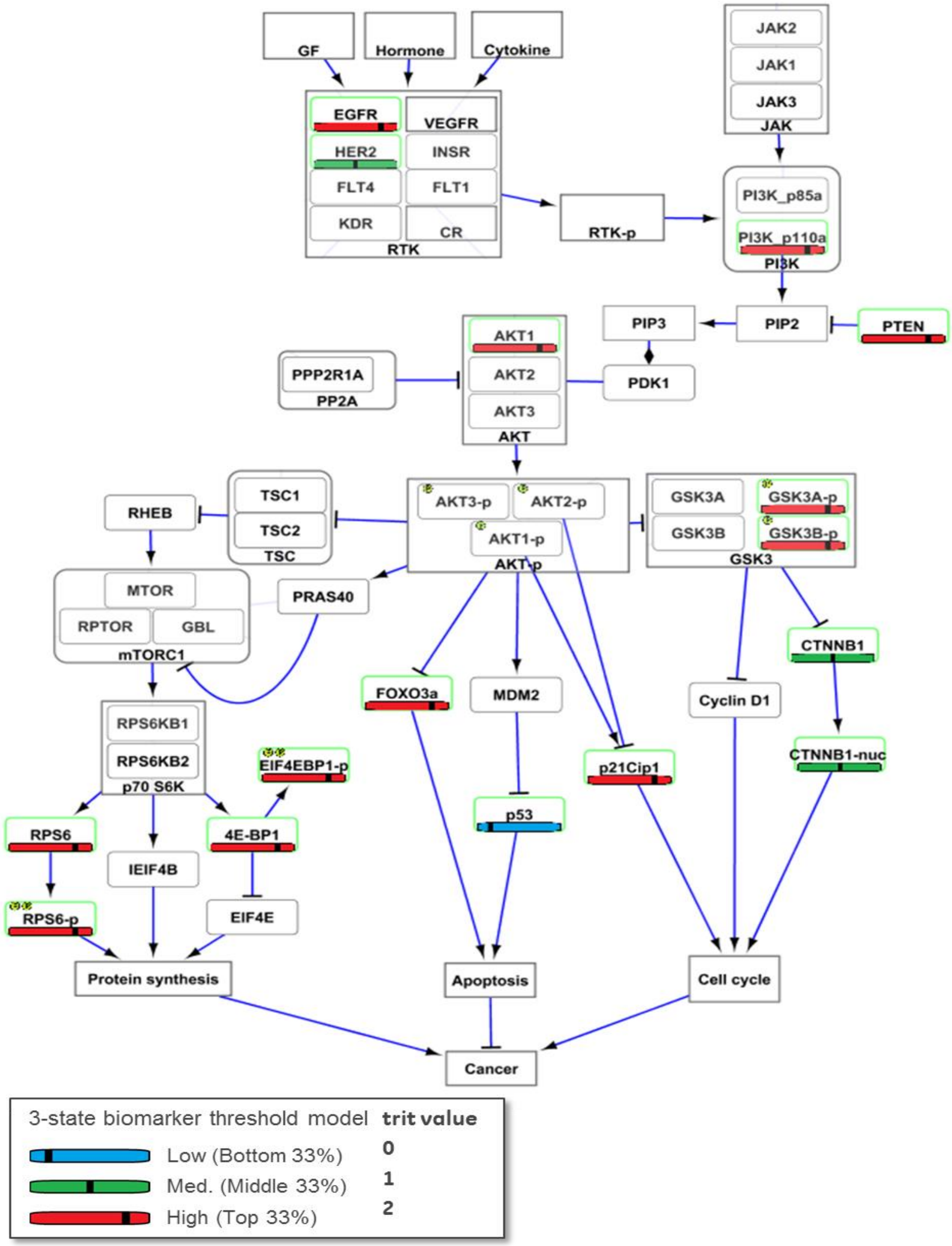

Figure A. The AKT signaling pathway with links to apoptosis, cell cycle, and protein synthesis. The pathway has 16 measurable nodes, each with a red, green, or blue horizontal colored bar representing high, medium, and low protein IF measurements respectively. For the example shown, the measurable nodes define the state of the pathway as 2122202222211222. The details on the nodes of the pathway are listed in Supplementary Table A. Cytoscape was used for visualizing the pathway (Shannon, et al., 2003).

Table A. Details on the 16 measurable nodes of the AKT pathway ordered to represent the state 2122202222211222 shown in the color-coded pathway map Figure A.

Order	Node Value	Node Label	Protein ID (UniProt)	Gene of Protein	Phosphorylation Modification	Cellular Location
1	2	EGFR	P00533	EGFR		cell
2	1	HER2	P04626	ERBB2		cell
3	2	PI3K_p110a	P42336	PIK3CA		cytosol
4	2	PTEN	P60484	PTEN		cytosol
5	2	AKT1	P31749	AKT1		cytosol
6	0	p53	P04637	TP53		nuclear
7	2	FOXO3a	O43524	FOXO3		cytosol
8	2	4E-BP1	Q13541	EIF4EBP1		cytosol
9	2	EIF4EBP1-p	Q13541	EIF4EBP1	pT37 and pT46	cytosol
10	2	GSK3A-p	P49840	GSK3A	pS21	cytosol
11	2	GSK3B-p	P49841	GSK3B	pS9	cytosol
12	1	CTNNB1	P35222	CTNNB1		cytosol
13	1	CTNNB1-nuc	P35222	CTNNB1		nuclear
14	2	p21Cip1	P38936	CDKN1A		nuclear
15	2	RPS6	P62753	RPS6		cytosol
16	2	RPS6-p	P62753	RPS6	pS235 and pS236	cytosol

Table B. The cancer hallmarks (Hanahan and Weinberg, 2000; Hanahan and Weinberg, 2011; Knijnenburg, et al., 2015) and the corresponding mapping of genes to those cancer hallmarks. The table presents the number of genes mapped to each hallmark (Knijnenburg, et al., 2015) and the subsets of genes that had corresponding antibody markers used in the CRC cancer tissue imaging study.

Gene Set Name	Set Size	Num. Measured Genes	Subset of genes with corresponding antibody markers used in the study
Activating Invasion and Metastasis	626	14	CTNNB1, FN1, ERBB2, EGFR, WNT5A, CDH1, PTEN, AKT1, CASP3, MAPK1, MAPK3, CD3E, MET, PIK3CA
Avoiding Immune Destruction	153	6	IHH, CD8A, MAPK1, MAPK3, CD3E, PIK3CA
Evading Growth Suppressors	187	7	EGFR, FOXO3, CDKN1A, FOXO1, AKT1, MET, PIK3CA
Genome instability and Mutation	127	3	CASP3, PCNA, TP53
Inducing Angiogenesis	110	6	CTNNB1, FN1, AKT1, MAPK1, MAPK3, PIK3CA
Resisting Cell Death	415	21	CTNNB1, RPS6, VIM, ERBB2, EGFR, FOXO3, CDH1, LMNA, CDKN1A, CD8A, CD79A, PTEN, FOXO1, AKT1, CASP3, MAPK1, MAPK3, CD3E, MET, PIK3CA, TP53
Sustaining Proliferative Signaling	349	12	CTNNB1, ERBB2, EGFR, CD79A, FOXO1, AKT1, CASP3, MAPK1, MAPK3, PIK3CA, CCNB1, TP53

2. Defining cell neighbors for spatial metrics

Instead of the accurate but time consuming pixel-based, an approximate approach was developed to identify touching cell neighbors. The Euclidean distance between the centers of two cells (xi, yi) and (xj, yj) was computed and normalized by the sum of the approximate radii of the two cells (ri and rj). The cell radii were computed from the segmented area of the cells (Ai, Aj), approximating the cells on the 2D images as circles. If this normalized Euclidean distance is equal to or less than an dimensionless critical parameter, dcritical , the cells i and j are then considered to be touching neighbors.

$$\sqrt{\frac{(x_i - x_j)^2 + (y_i - y_j)^2}{(r_i + r_j)^2}} \leq d_{critical} \quad where\ r_i = \sqrt{\frac{A_i}{\pi}}$$

To establish the value of the dimensionless critical parameter, dcritical, the approximate method was compared to the exact method for over 1.5 billion cell pairs from the colorectal cancer data set. The change in the number of correctly and falsely identified touching cell neighbors as a function of the critical parameter was computed. A critical parameter of 1.31 minimized the number of false predictions, resulting in the best agreement between the approximate and exact methods (Figure B).

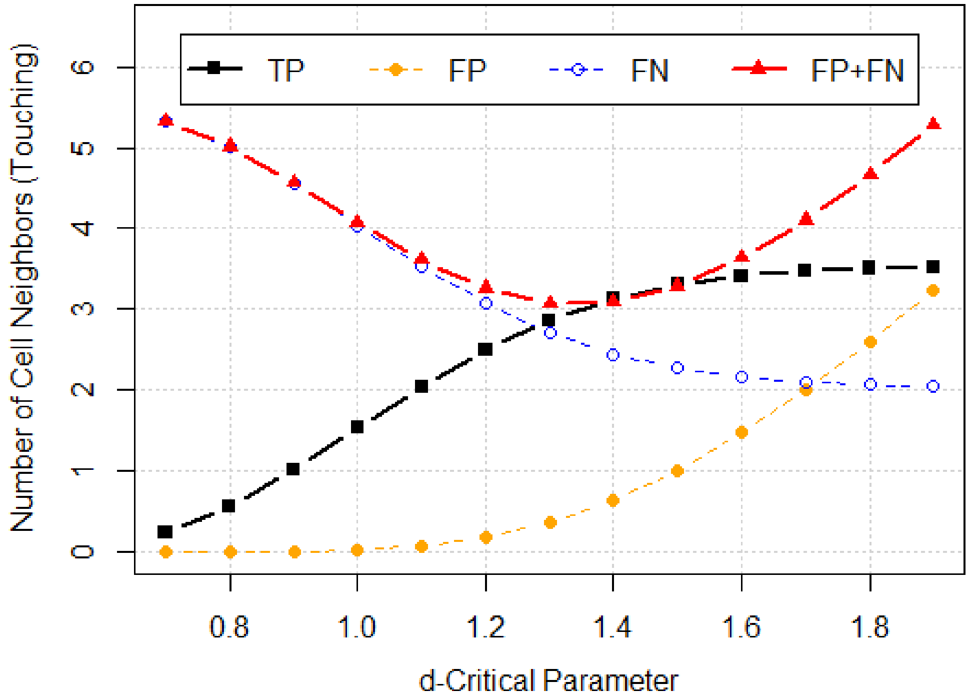

Figure B. Change in the number of correct cell neighbors (True Positives = black squares), false positive assignments (orange filled circles) and those that were incorrectly assigned as not being neighbors (False Negatives – blue open circles) to a cell being evaluated. The change in the TP, FP, and FN were computed by comparing the assignment of the approximate method of computing cell neighbors relative to the exact method using the cell's segmented image pixels. Over 1.5 billion cell pair comparisons were evaluated and used to generate the curves. The best performance of the approximate method was achieved at the dimensionless critical parameter, d-critical, value of 1.31, which minimized the total number of false assignments (FP + FN).

3. Maximum number of cell social states

The maximum number of cell social states, Ns, that is theoretically possible is dependent upon the total number of cells, Nc, present in the system. Each cell social state is a group of cells of a unique number. Summing over all possible cell social states will compute the minimum number of cells required to observe all those states. This is mathematically represented as:

$$\sum_{i}^{N_S} i = \frac{N_S(N_S+1)}{2} \leq N_C$$

Solving the inequality leads to the formula for computing the maximum number of possible cell social states, NS, for a system with Nc cells:

$$N_S = Int\left(\frac{\sqrt{8N_C+1}-1}{2}\right)$$

4. Colorectal cancer (CRC) cohort dataset

Tissue samples of colorectal cancer (CRC) patients were collected at the Clearview Cancer Institute of Huntsville Alabama and provided to GE Global Research by Clarient Inc. This tissue microarray (TMA) imaging cohort consisted of 747 paraffin-embedded patient tumor core samples distributed across three slides. A breakdown of samples by histological grade and cancer stage in shown in Table C. The number of patients with or without a reoccurrence event during follow-up, broken down by cancer stage and treatment protocol is shown in Table D.

Table C. Breakdown of CRC cohort samples by histological tumor grade and cancer stage. The histologic grade characterizes the observed degree of tumor differentiation while the tumor stage characterizes the severity of the cancer.

	Grade 1	Grade 2	Grade 3	Totals
**Stage 1**	46 (25%)	128 (69%)	11 (6%)	185
**Stage 2**	37 (14%)	208 (79%)	19 (7%)	264
**State 3**	22 (9%)	171 (70%)	50 (21%)	243
**Totals**	105 (15%)	507 (73%)	80 (12%)	692

Table D. The number of patients without or with a reoccurrence event during follow-up in the CRC cohort broken down by cancer stage and treatment protocol.

	Follow-up Reoccurrence Event [No / Yes (%)]		
	**No Chemo**	**Chemo Treated Fluorouracil (FU)**	**Totals**
**Stage 1**	140 / 8 (5.4%)	31 / 6 (16.2%)	171 / 14 (7.6%)
**Stage 2**	122 / 16 (11.6%)	97 / 29 (23%)	219 / 45 (17%)
**State 3**	42 / 20 (32.3%)	112 / 69 (38.1%)	154 / 89 (36.6%)
**Totals**	304 / 44 (12.6%)	240 / 104 (30.2%)	544 / 148 (21.4%)

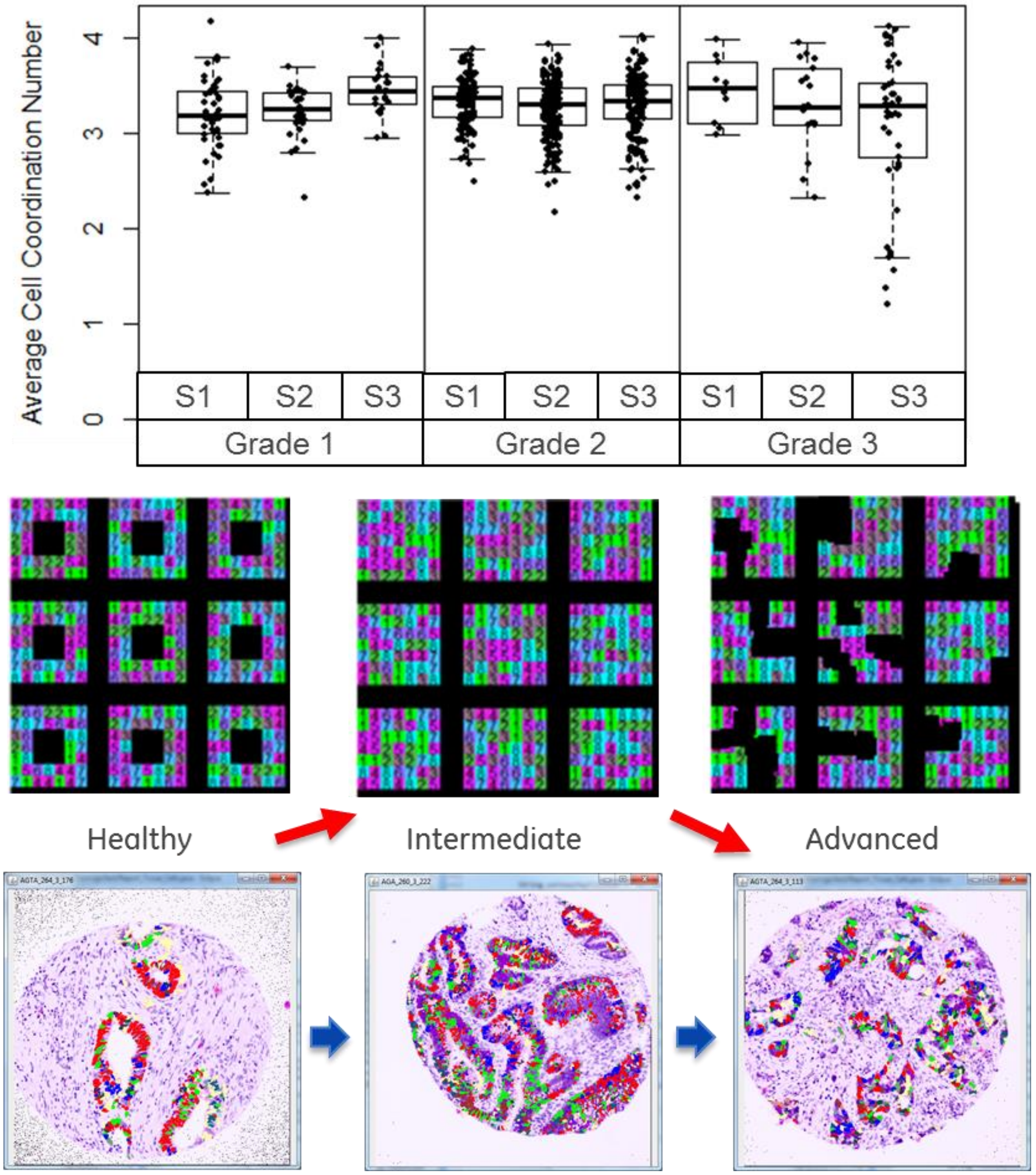

Figure C. The cell coordination number, a purely spatial metric, show an interesting behavior: increases with stage in grade 1 tumors, stays relatively unchanged for grade 2, followed by a decreasing trend with stage for grade 3 tumors. We speculate that the metric reflects the longitudinal changes in tumor spatial structure from the healthy or nearly healthy gland, through a more compact structure with increasing number of cell neighbors, to a collapsed, irregular structure in which epithelial cells become more isolated again. The red arrows indicate the trend in the average coordination number.

References

Hanahan, D. and Weinberg, R.A. (2000) The hallmarks of cancer, *Cell*, **100**, 57-70.
Hanahan, D. and Weinberg, R.A. (2011) Hallmarks of cancer: the next generation, *Cell*, **144**, 646-674.
Knijnenburg, T.A.*, et al.* (2015) A multilevel pan-cancer map links gene mutations to cancer hallmarks, *Chin J Cancer*, **34**, 439-449.
Shannon, P.*, et al.* (2003) Cytoscape: a software environment for integrated models of biomolecular interaction networks, *Genome Res*, **13**, 2498-2504.

Section 2

Change in the number of correct and incorrect cell neighbor assignments (True Positives, False Positive, False Negatives) by comparing the assignment of the approximate method of computing cell neighbors relative to the exact method using the cell's segmented image pixels. Assignments broken out by cancer stage and tumor grade. Figures showing the correlations between the diversity metrics as calculated with the exact method of cell neighbor identification versus the approximate method.

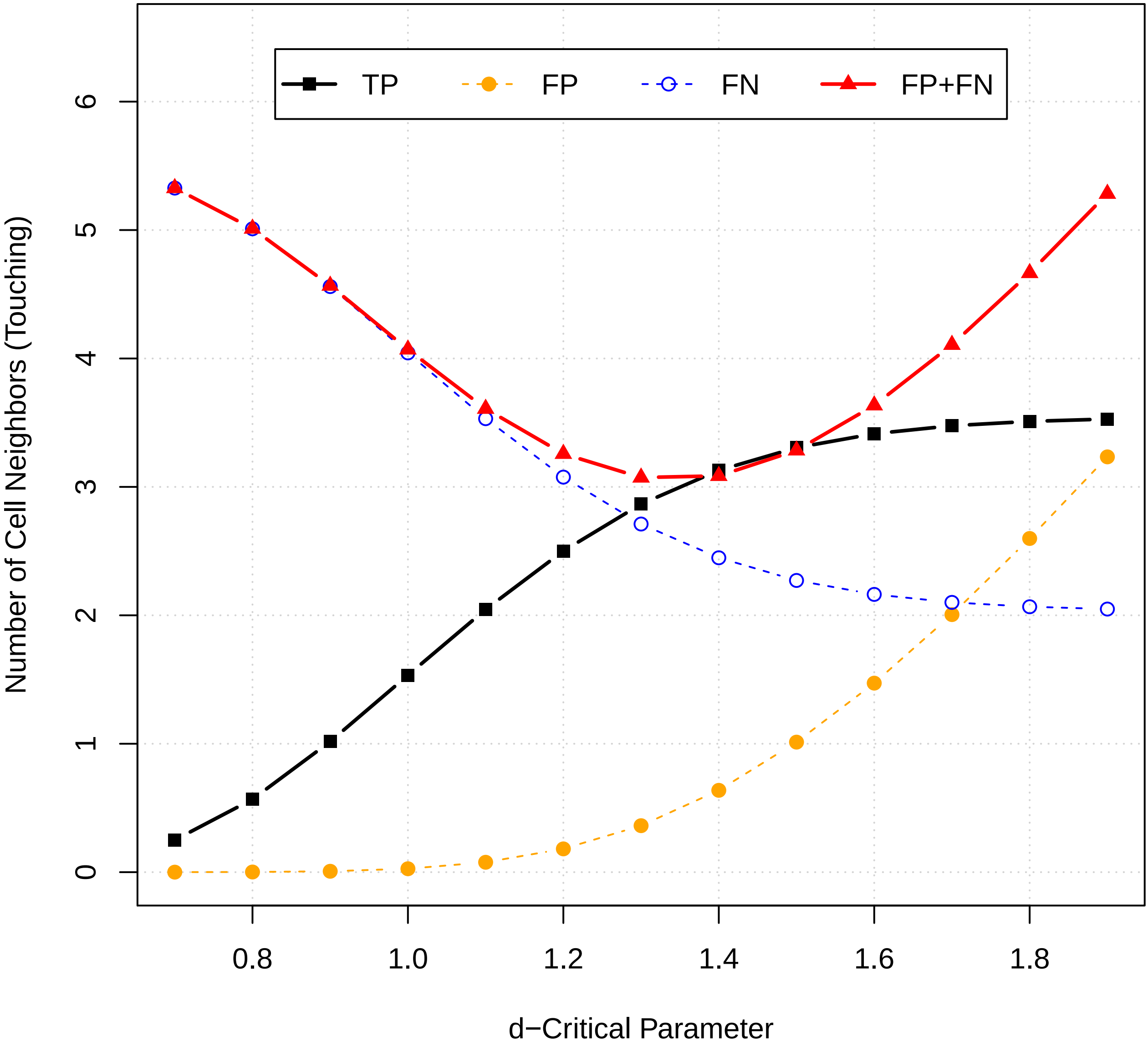
TP
FP
FN
FP+FN
Number of Cell Neighbors (Touching)
0
1
2
3
4
5
6
0.8
1.0
1.2
1.4
1.6
1.8
d−Critical Parameter

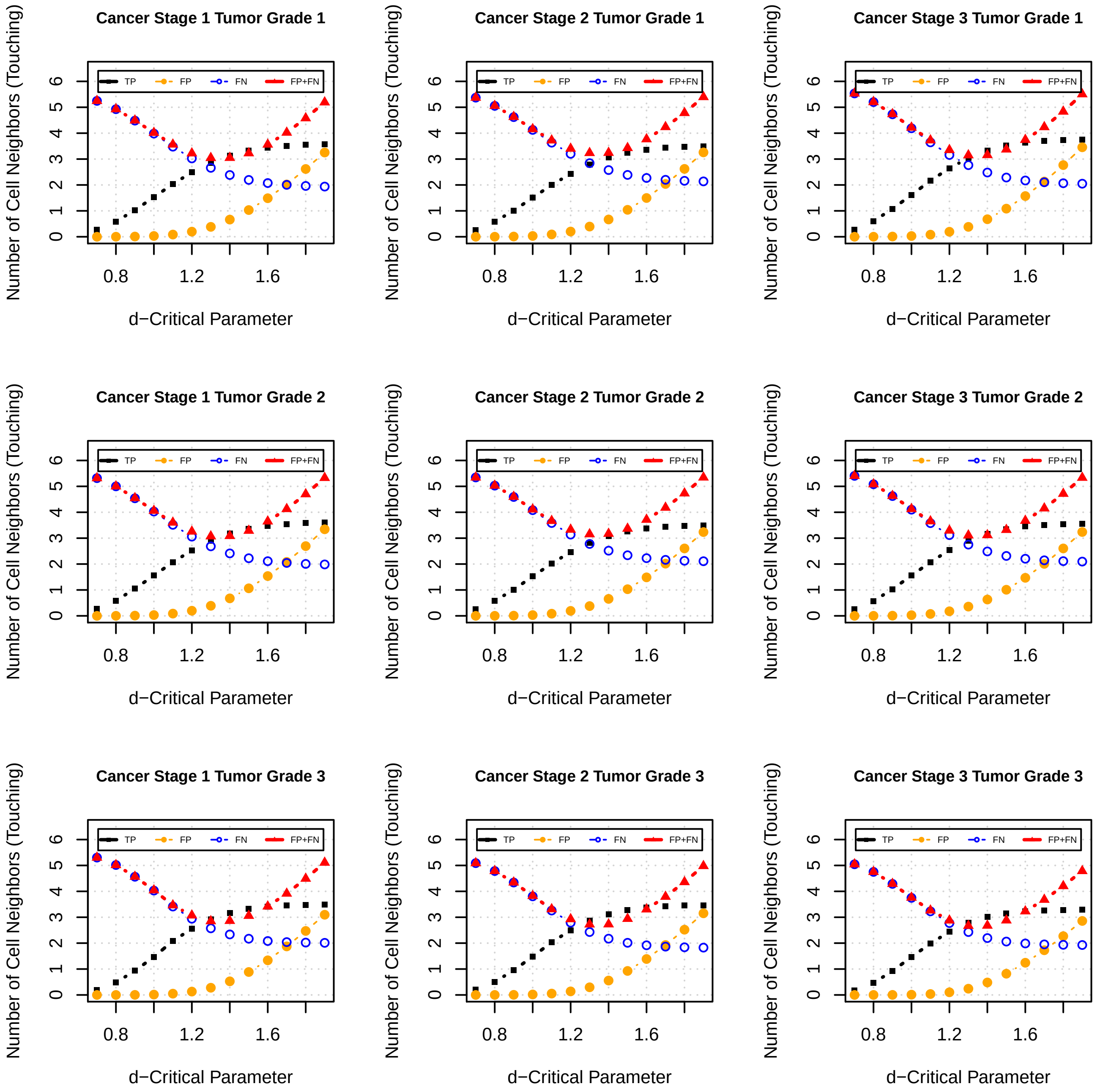
Cancer Stage 1 Tumor Grade 1
Cancer Stage 2 Tumor Grade 1
Cancer Stage 3 Tumor Grade 1
Cancer Stage 1 Tumor Grade 2
Cancer Stage 2 Tumor Grade 2
Cancer Stage 3 Tumor Grade 2
Cancer Stage 1 Tumor Grade 3
Cancer Stage 2 Tumor Grade 3
Cancer Stage 3 Tumor Grade 3
TP
FP
FN
FP+FN
Number of Cell Neighbors (Touching)
d−Critical Parameter
0
1
2
3
4
5
6
0.8
1.2
1.6

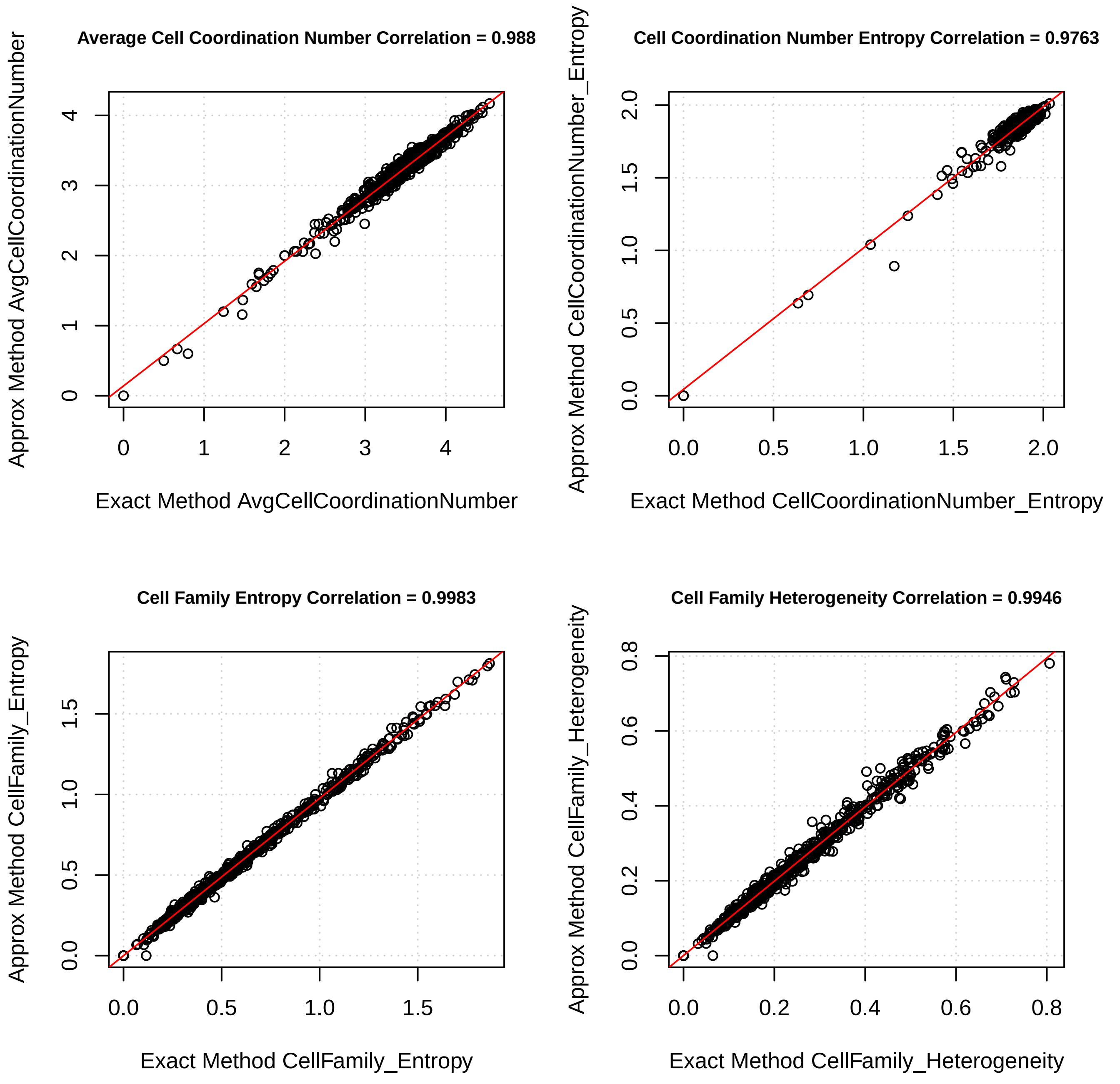

Average Cell Coordination Number Correlation = 0.988
Approx Method AvgCellCoordinationNumber
Exact Method AvgCellCoordinationNumber
0
1
2
3
4
Cell Coordination Number Entropy Correlation = 0.9763
Approx Method CellCoordinationNumber_Entropy
Exact Method CellCoordinationNumber_Entropy
0.0
0.5
1.0
1.5
2.0
Cell Family Entropy Correlation = 0.9983
Approx Method CellFamily_Entropy
Exact Method CellFamily_Entropy
0.0
0.5
1.0
1.5
Cell Family Heterogeneity Correlation = 0.9946
Approx Method CellFamily_Heterogeneity
Exact Method CellFamily_Heterogeneity
0.0
0.2
0.4
0.6
0.8

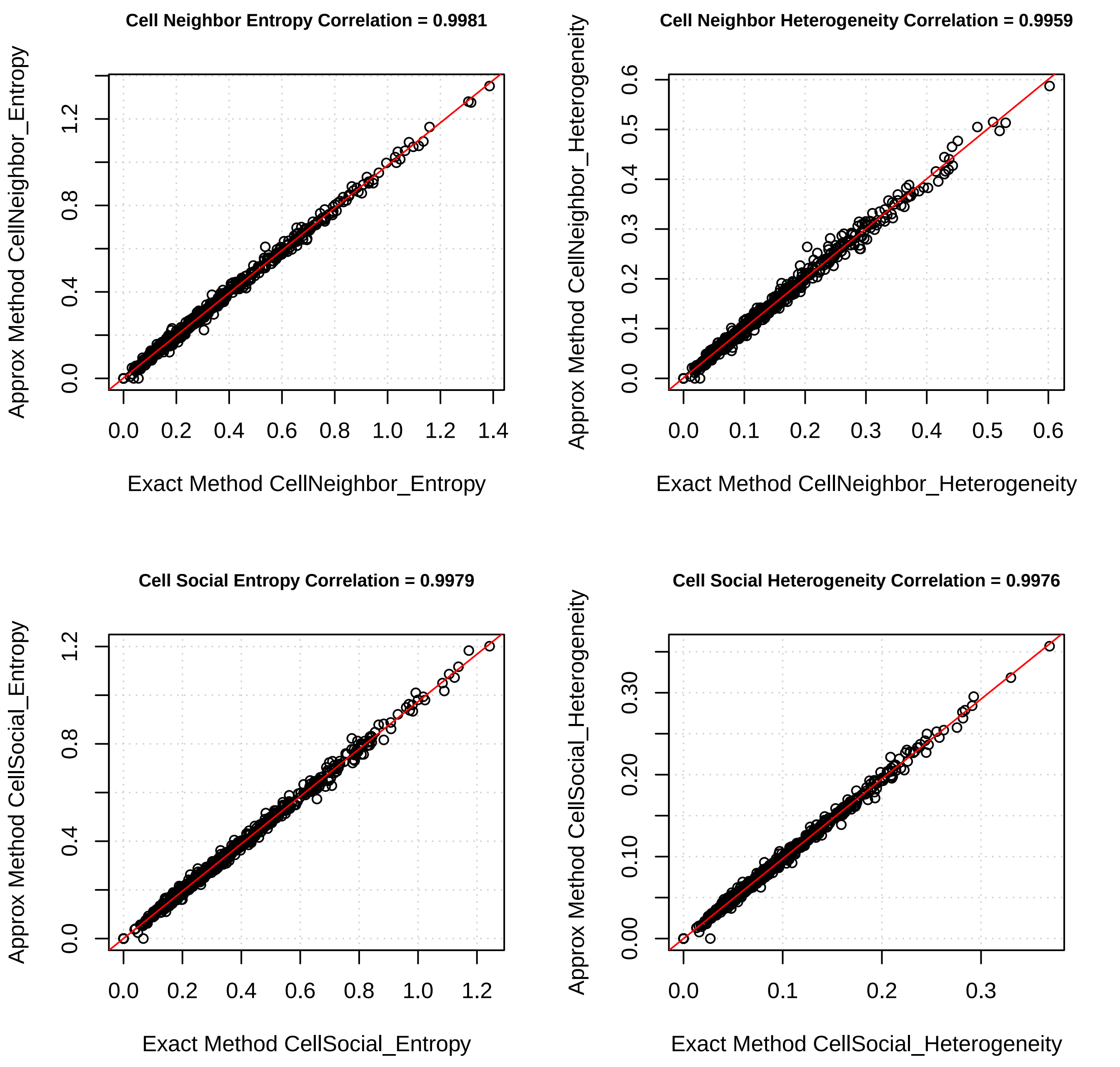

Cell Neighbor Entropy Correlation = 0.9981
Approx Method CellNeighbor_Entropy
Exact Method CellNeighbor_Entropy
0.0
0.4
0.8
1.2
0.0
0.2
0.4
0.6
0.8
1.0
1.2
1.4
Cell Neighbor Heterogeneity Correlation = 0.9959
Approx Method CellNeighbor_Heterogeneity
Exact Method CellNeighbor_Heterogeneity
0.0
0.1
0.2
0.3
0.4
0.5
0.6
0.0
0.1
0.2
0.3
0.4
0.5
0.6
Cell Social Entropy Correlation = 0.9979
Approx Method CellSocial_Entropy
Exact Method CellSocial_Entropy
0.0
0.4
0.8
1.2
0.0
0.2
0.4
0.6
0.8
1.0
1.2
Cell Social Heterogeneity Correlation = 0.9976
Approx Method CellSocial_Heterogeneity
Exact Method CellSocial_Heterogeneity
0.00
0.10
0.20
0.30
0.0
0.1
0.2
0.3

Section 3

Box charts with diversity metrics by cancer stage and tumor grade

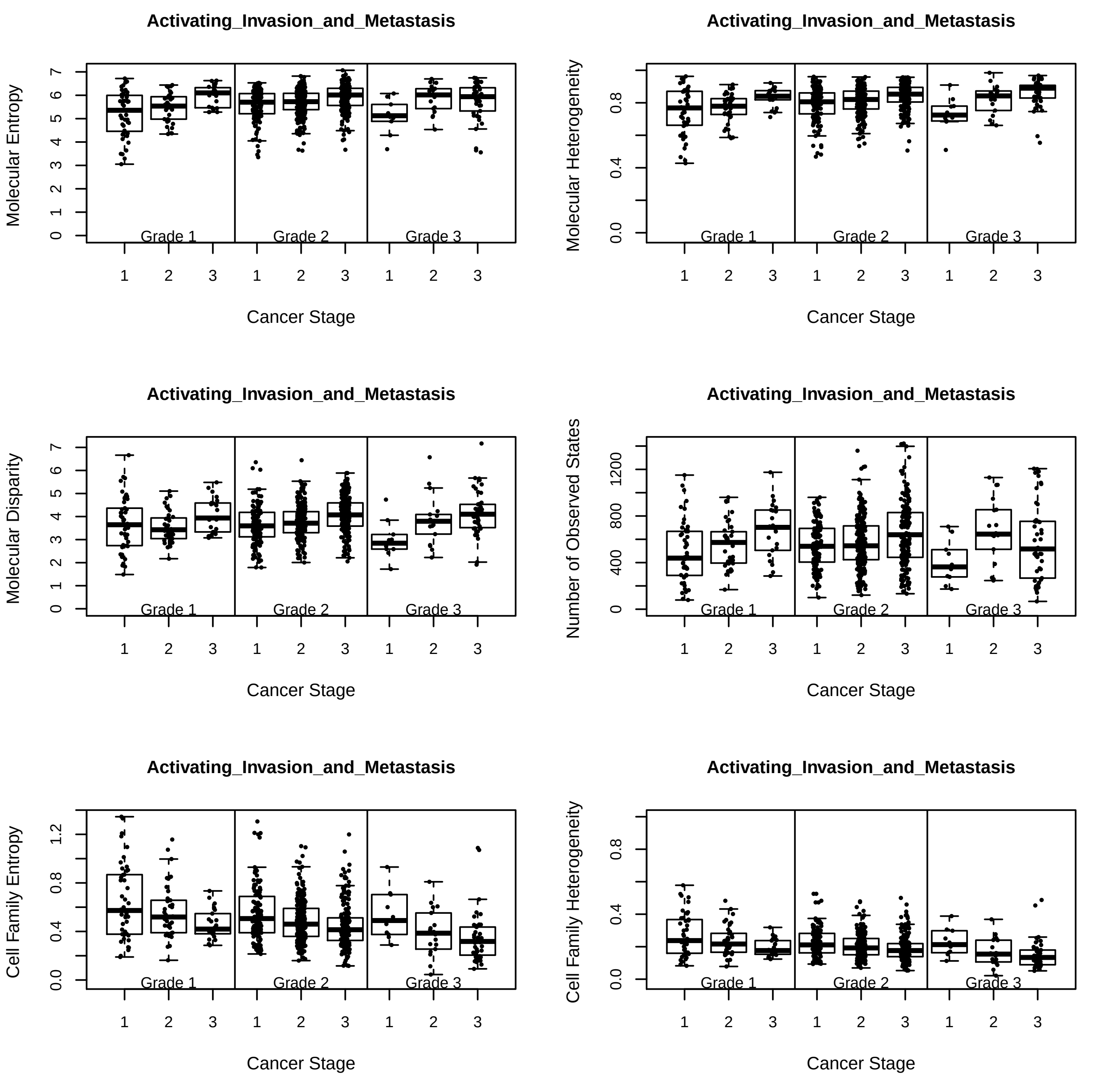
Activating_Invasion_and_Metastasis
Molecular Entropy
0 1 2 3 4 5 6 7
Grade 1
Grade 2
Grade 3
1 2 3 1 2 3 1 2 3
Cancer Stage
Activating_Invasion_and_Metastasis
Molecular Heterogeneity
0.0 0.4 0.8
Grade 1
Grade 2
Grade 3
1 2 3 1 2 3 1 2 3
Cancer Stage
Activating_Invasion_and_Metastasis
Molecular Disparity
0 1 2 3 4 5 6 7
Grade 1
Grade 2
Grade 3
1 2 3 1 2 3 1 2 3
Cancer Stage
Activating_Invasion_and_Metastasis
Number of Observed States
0 400 800 1200
Grade 1
Grade 2
Grade 3
1 2 3 1 2 3 1 2 3
Cancer Stage
Activating_Invasion_and_Metastasis
Cell Family Entropy
0.0 0.4 0.8 1.2
Grade 1
Grade 2
Grade 3
1 2 3 1 2 3 1 2 3
Cancer Stage
Activating_Invasion_and_Metastasis
Cell Family Heterogeneity
0.0 0.4 0.8
Grade 1
Grade 2
Grade 3
1 2 3 1 2 3 1 2 3
Cancer Stage

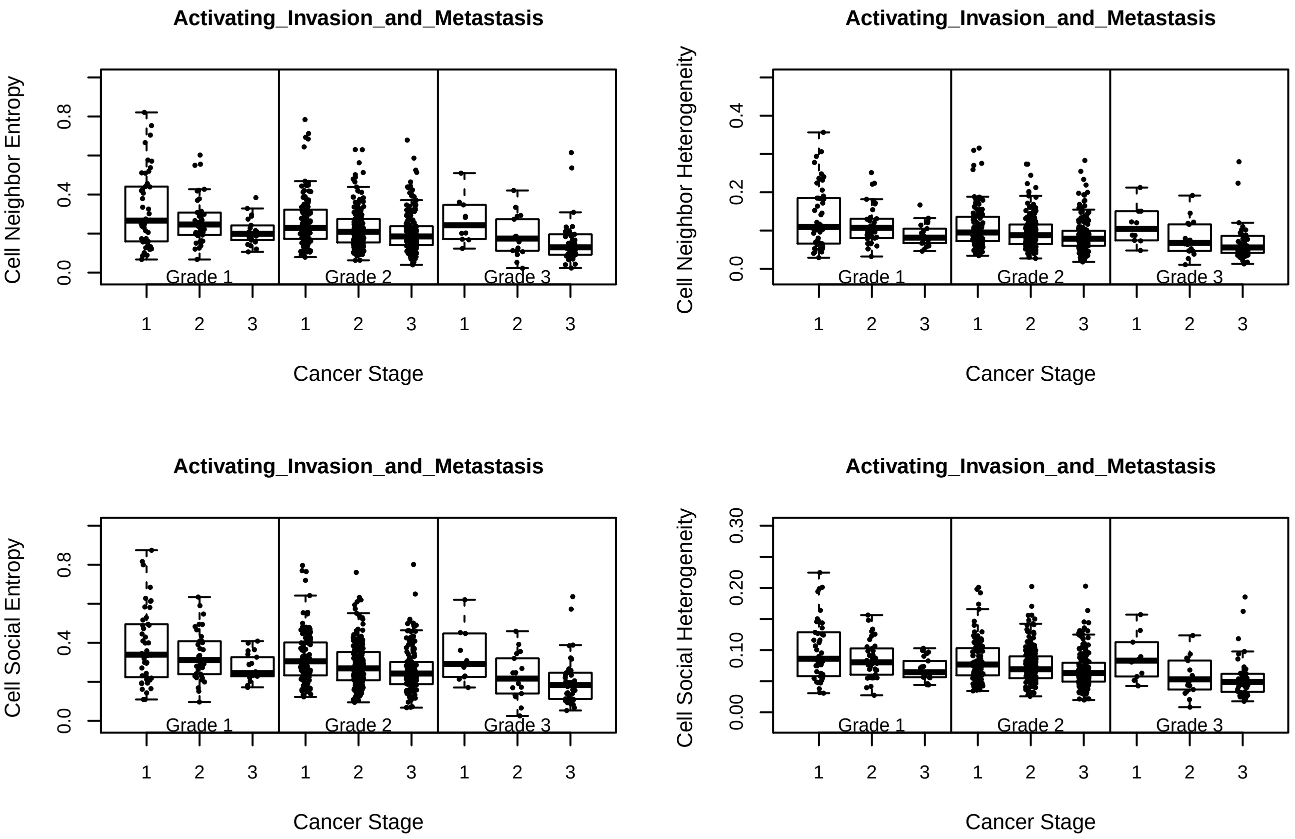
Activating_Invasion_and_Metastasis
Cell Neighbor Entropy
0.0
0.4
0.8
Grade 1
Grade 2
Grade 3
1 2 3 1 2 3 1 2 3
Cancer Stage
Activating_Invasion_and_Metastasis
Cell Neighbor Heterogeneity
0.0
0.2
0.4
Grade 1
Grade 2
Grade 3
1 2 3 1 2 3 1 2 3
Cancer Stage
Activating_Invasion_and_Metastasis
Cell Social Entropy
0.0
0.4
0.8
Grade 1
Grade 2
Grade 3
1 2 3 1 2 3 1 2 3
Cancer Stage
Activating_Invasion_and_Metastasis
Cell Social Heterogeneity
0.00
0.10
0.20
0.30
Grade 1
Grade 2
Grade 3
1 2 3 1 2 3 1 2 3
Cancer Stage

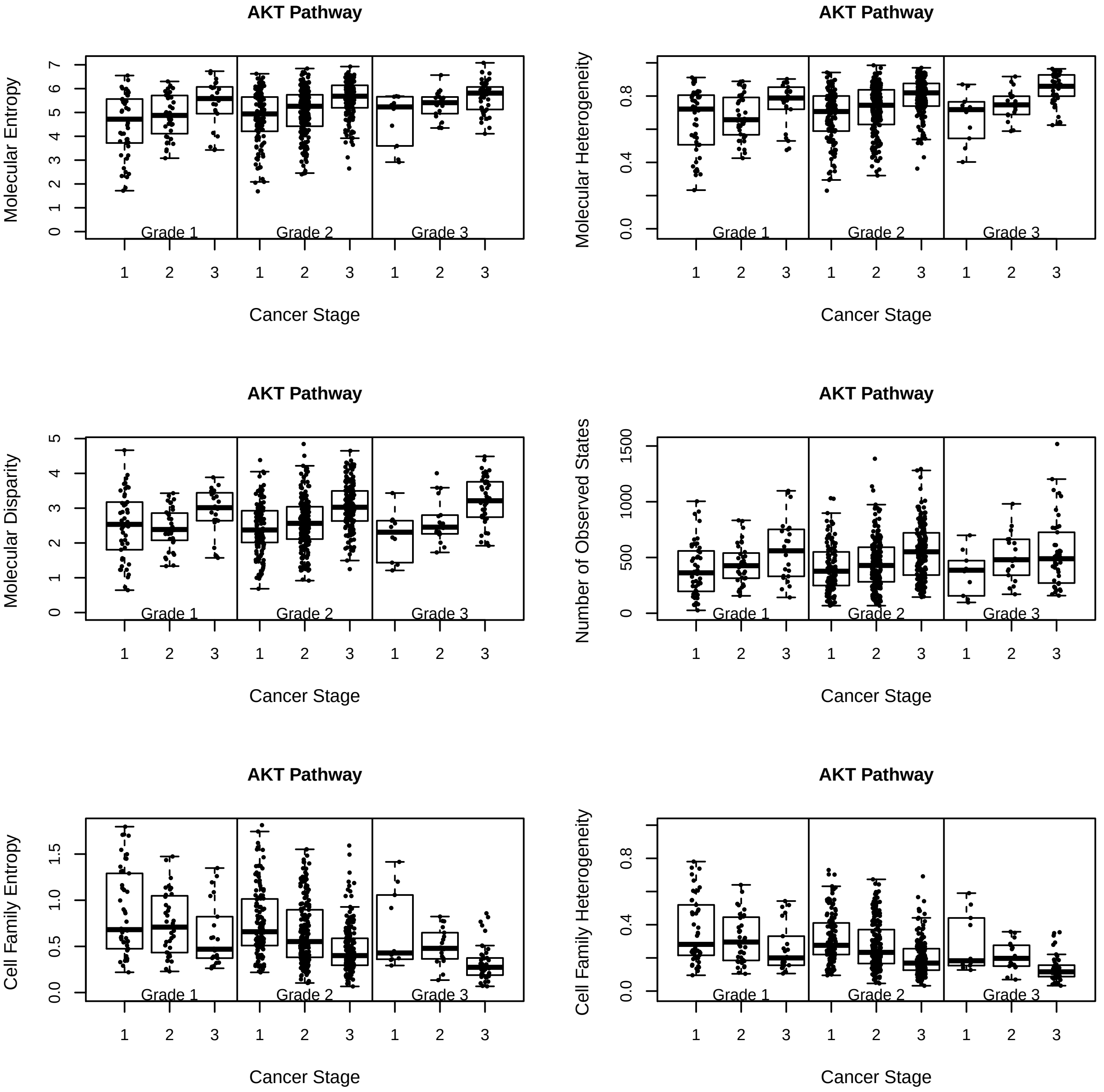
AKT Pathway
Molecular Entropy
Grade 1
Grade 2
Grade 3
Cancer Stage
AKT Pathway
Molecular Heterogeneity
Grade 1
Grade 2
Grade 3
Cancer Stage
AKT Pathway
Molecular Disparity
Grade 1
Grade 2
Grade 3
Cancer Stage
AKT Pathway
Number of Observed States
Grade 1
Grade 2
Grade 3
Cancer Stage
AKT Pathway
Cell Family Entropy
Grade 1
Grade 2
Grade 3
Cancer Stage
AKT Pathway
Cell Family Heterogeneity
Grade 1
Grade 2
Grade 3
Cancer Stage

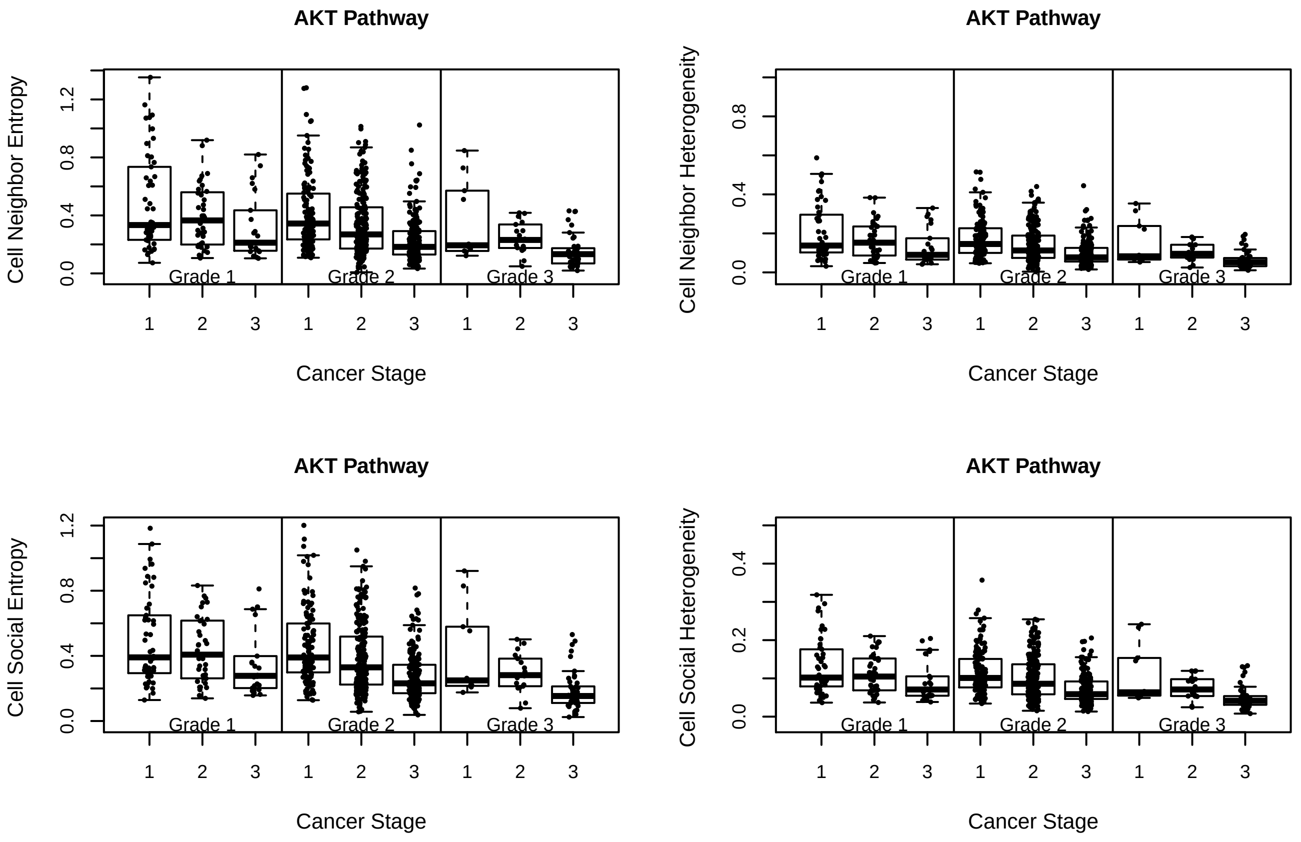
AKT Pathway
Cell Neighbor Entropy
Grade 1
Grade 2
Grade 3
Cancer Stage
AKT Pathway
Cell Neighbor Heterogeneity
Grade 1
Grade 2
Grade 3
Cancer Stage
AKT Pathway
Cell Social Entropy
Grade 1
Grade 2
Grade 3
Cancer Stage
AKT Pathway
Cell Social Heterogeneity
Grade 1
Grade 2
Grade 3
Cancer Stage

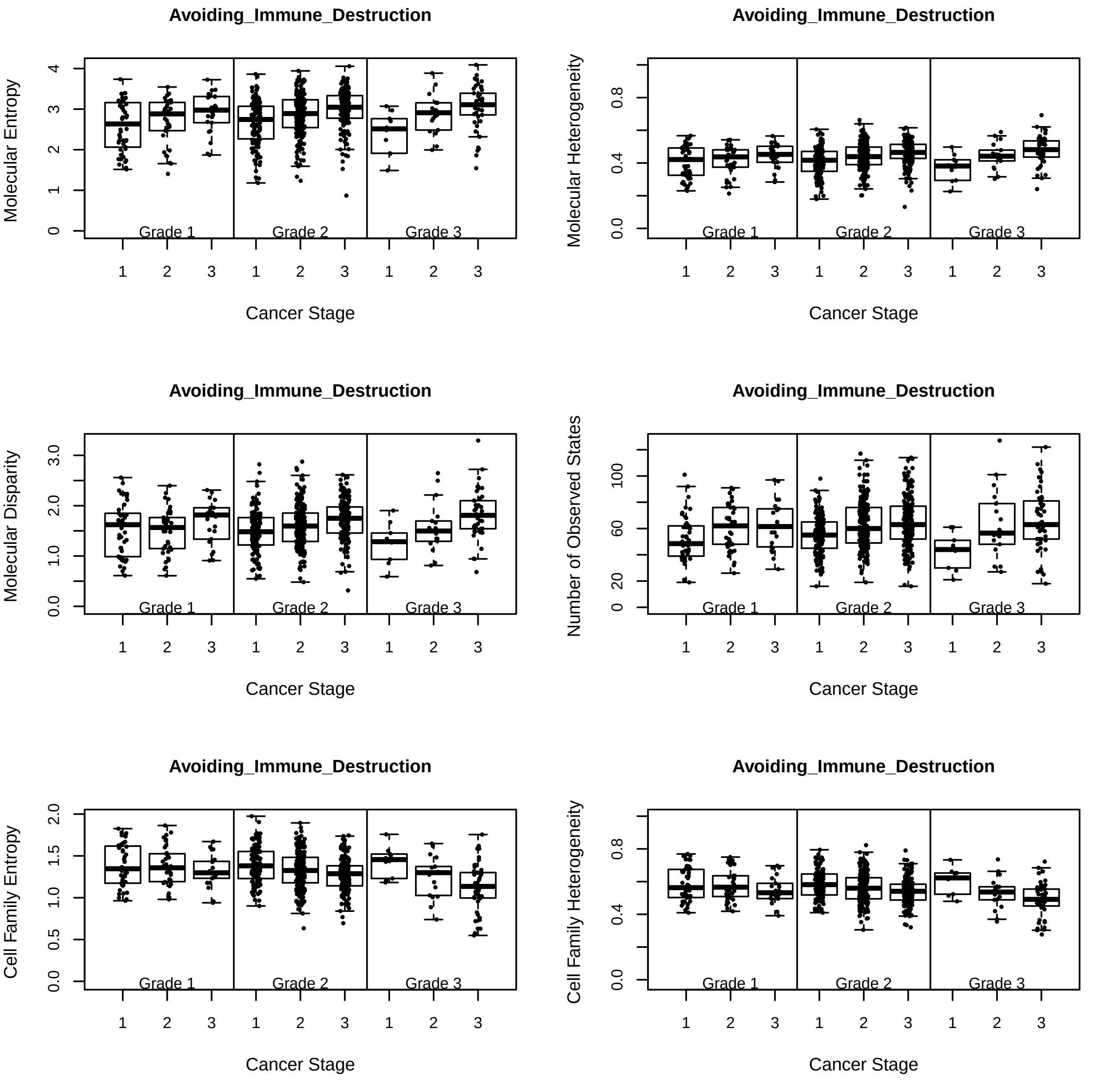
Avoiding_Immune_Destruction
Molecular Entropy
Molecular Heterogeneity
Molecular Disparity
Number of Observed States
Cell Family Entropy
Cell Family Heterogeneity
Grade 1
Grade 2
Grade 3
Cancer Stage

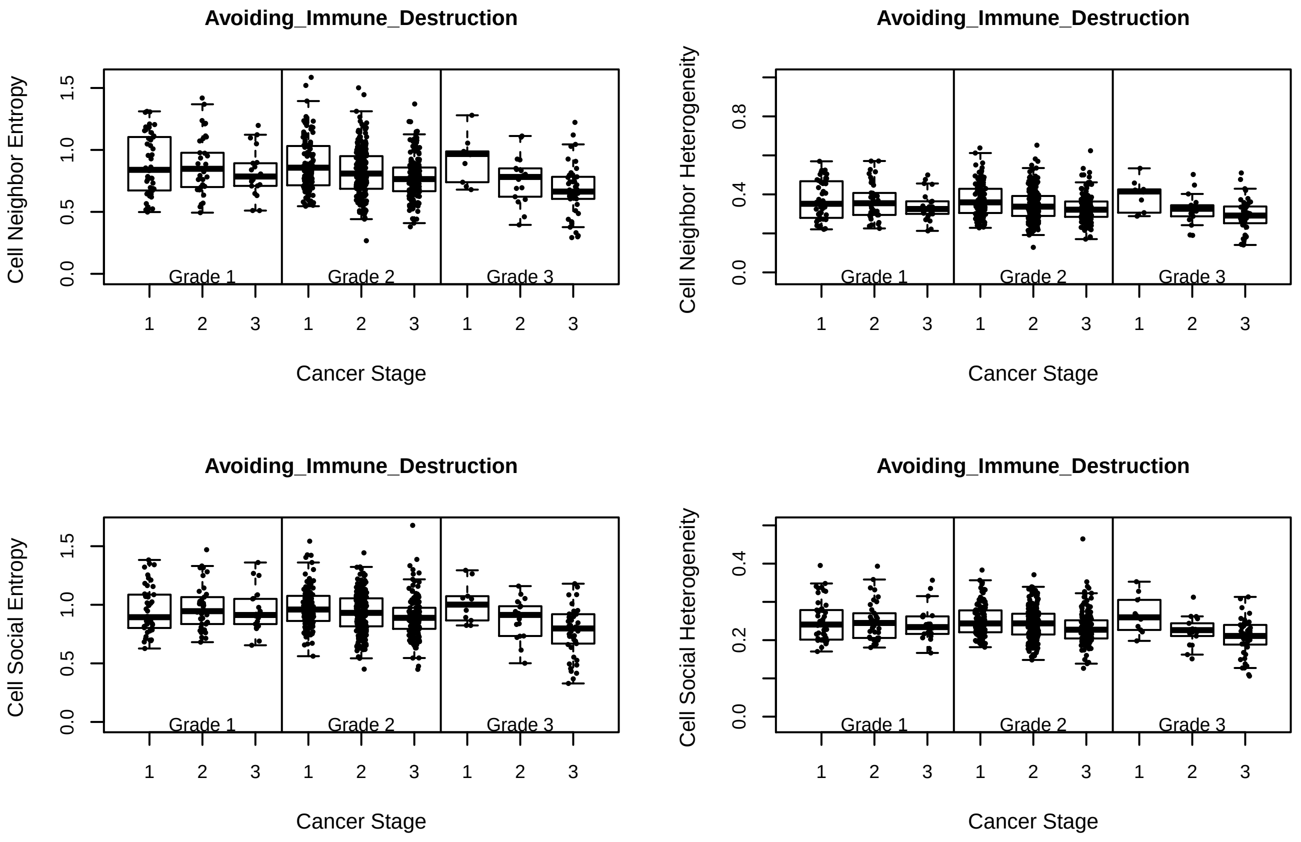
Avoiding_Immune_Destruction
Cell Neighbor Entropy
0.0
0.5
1.0
1.5
Grade 1
Grade 2
Grade 3
1 2 3 1 2 3 1 2 3
Cancer Stage
Avoiding_Immune_Destruction
Cell Neighbor Heterogeneity
0.0
0.4
0.8
Grade 1
Grade 2
Grade 3
1 2 3 1 2 3 1 2 3
Cancer Stage
Avoiding_Immune_Destruction
Cell Social Entropy
0.0
0.5
1.0
1.5
Grade 1
Grade 2
Grade 3
1 2 3 1 2 3 1 2 3
Cancer Stage
Avoiding_Immune_Destruction
Cell Social Heterogeneity
0.0
0.2
0.4
Grade 1
Grade 2
Grade 3
1 2 3 1 2 3 1 2 3
Cancer Stage

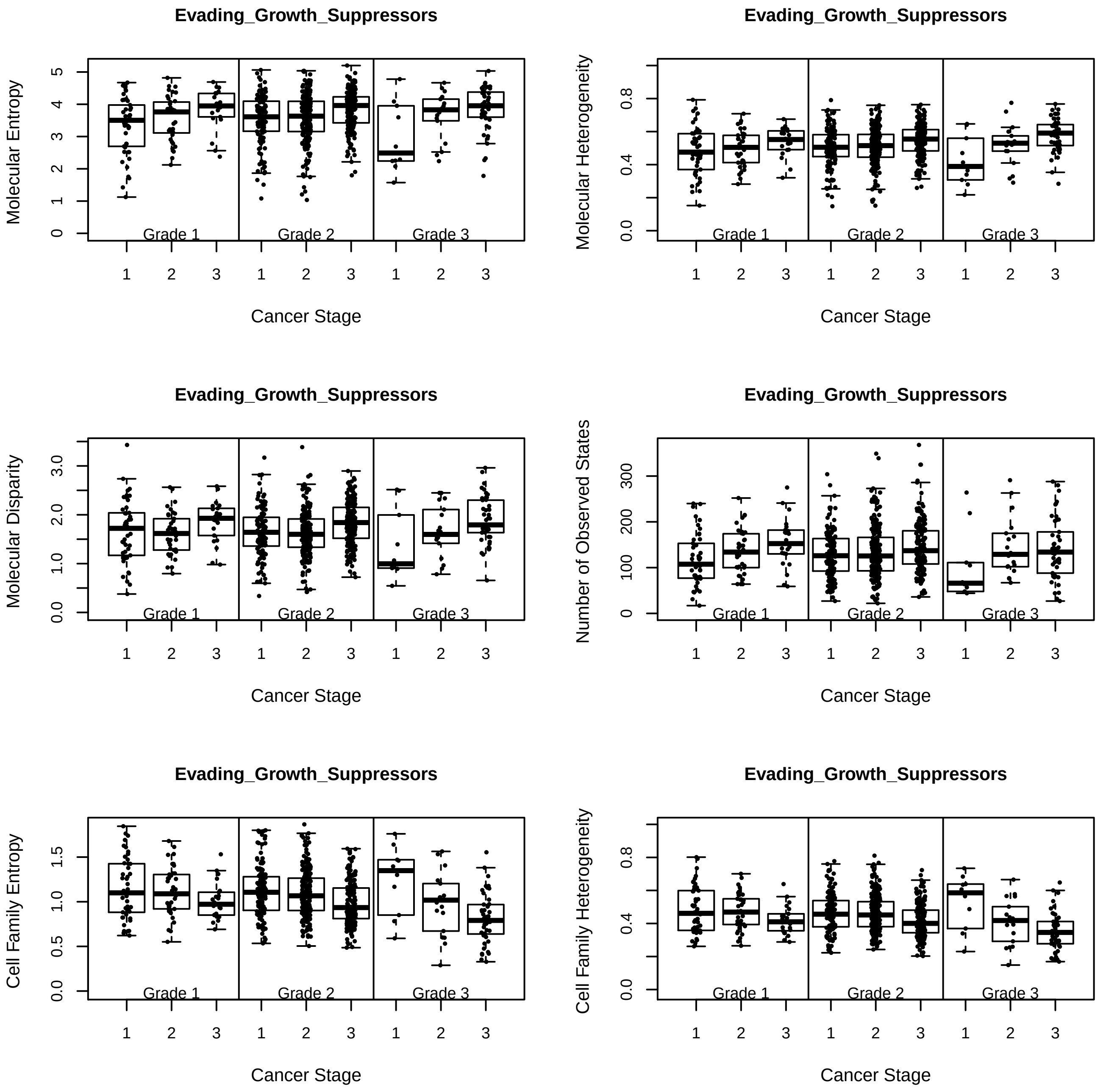
Evading_Growth_Suppressors
Molecular Entropy
Molecular Heterogeneity
Molecular Disparity
Number of Observed States
Cell Family Entropy
Cell Family Heterogeneity
Grade 1
Grade 2
Grade 3
1
2
3
Cancer Stage

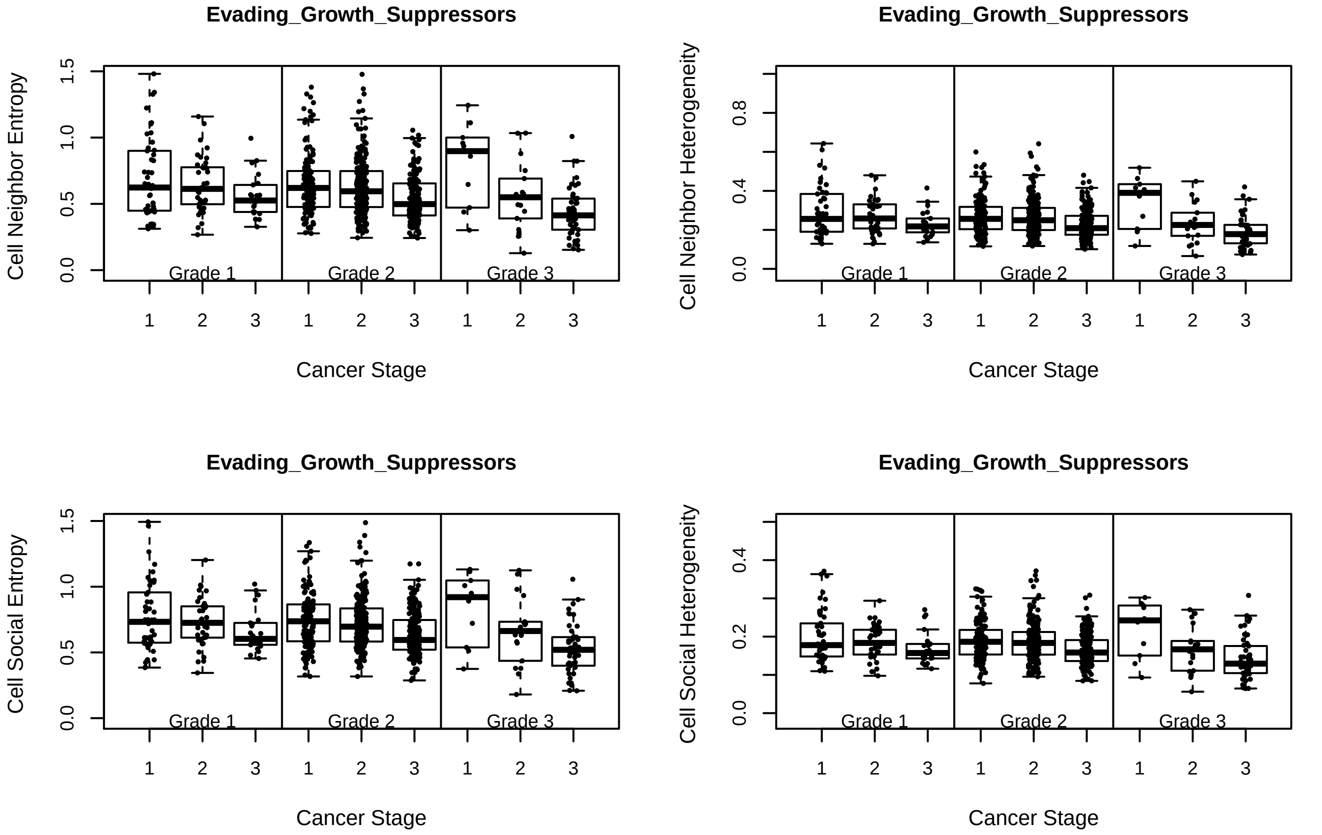
Evading_Growth_Suppressors
Cell Neighbor Entropy
Evading_Growth_Suppressors
Cell Neighbor Heterogeneity
Evading_Growth_Suppressors
Cell Social Entropy
Evading_Growth_Suppressors
Cell Social Heterogeneity
Grade 1
Grade 2
Grade 3
Cancer Stage

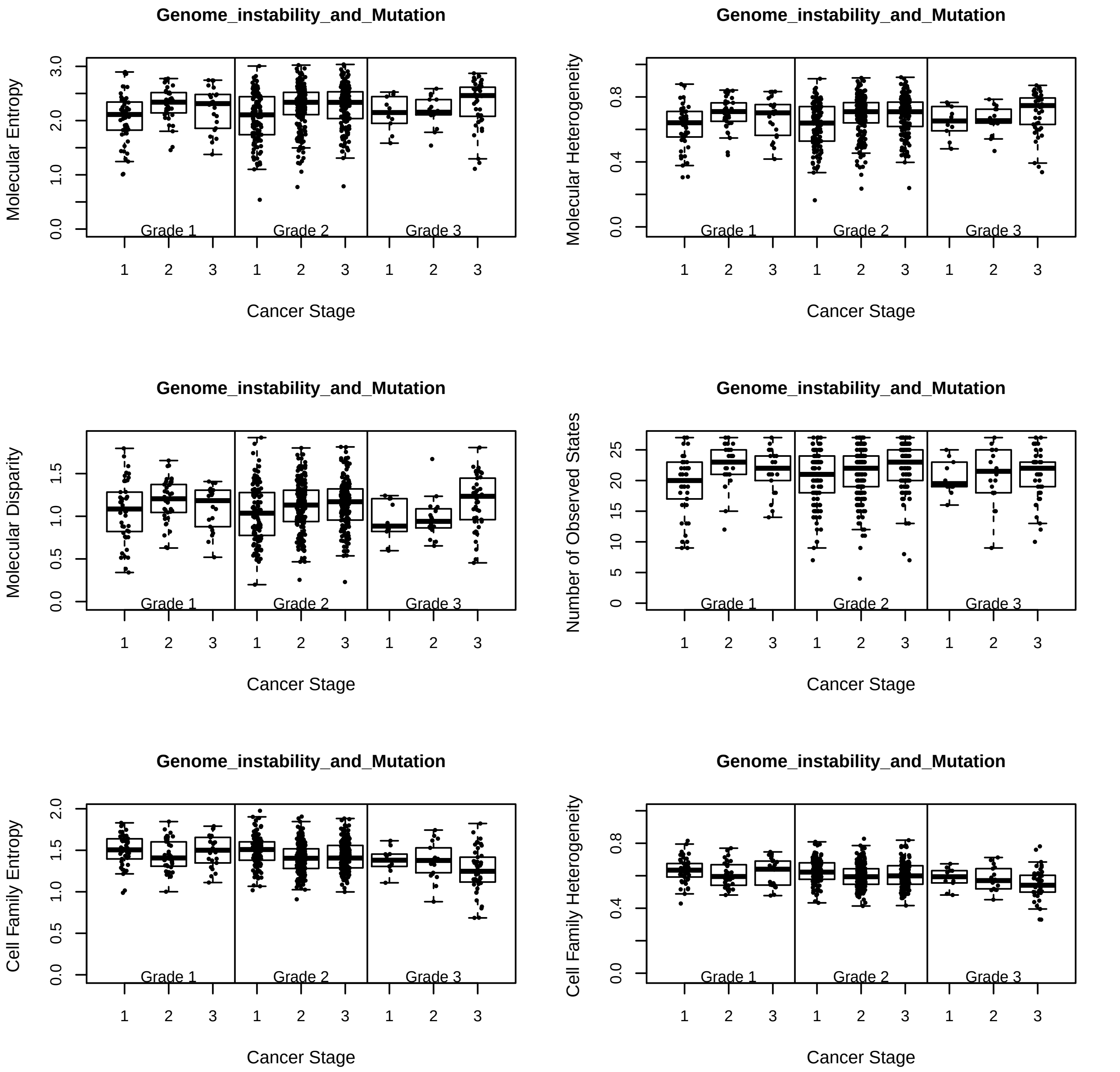
Genome_instability_and_Mutation
Molecular Entropy
Molecular Heterogeneity
Molecular Disparity
Number of Observed States
Cell Family Entropy
Cell Family Heterogeneity
Grade 1
Grade 2
Grade 3
Cancer Stage

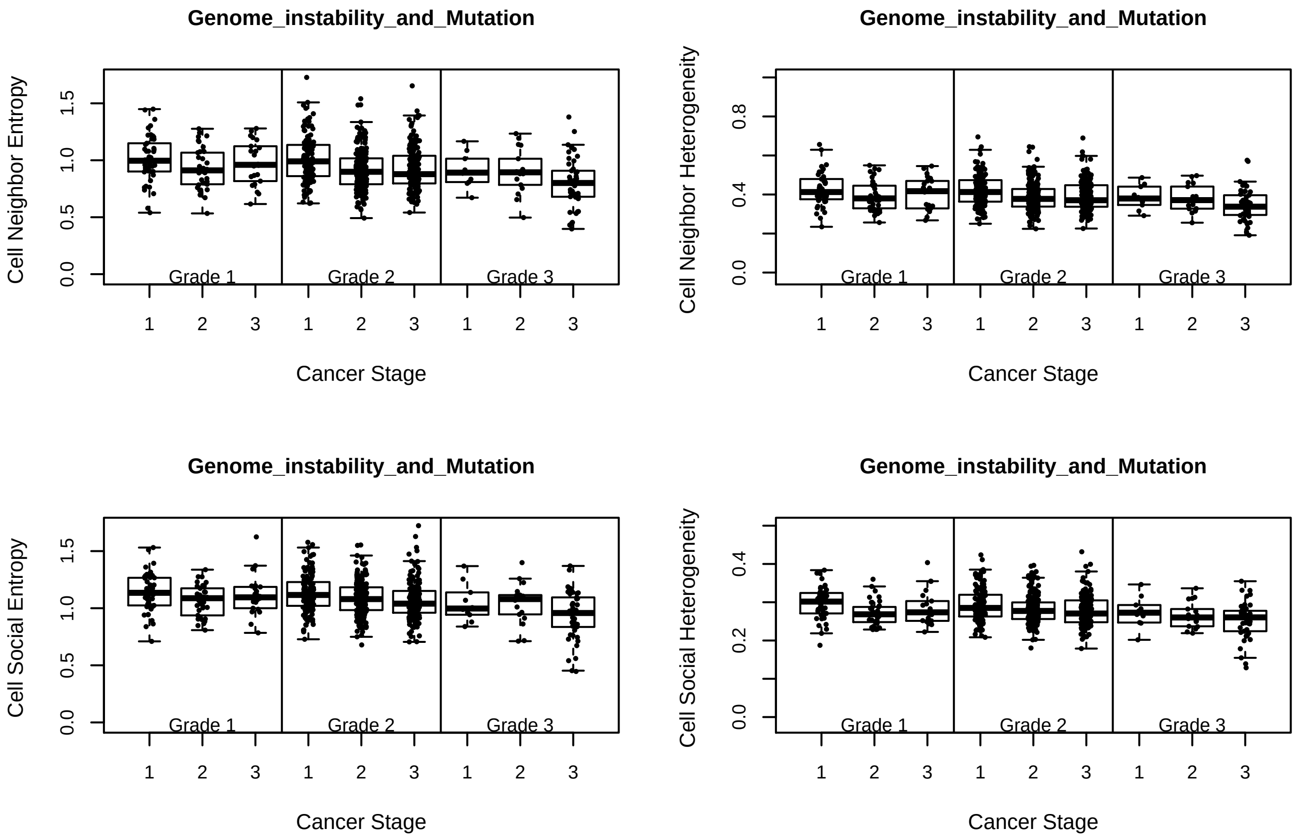
Genome_instability_and_Mutation
Cell Neighbor Entropy
Cancer Stage
Grade 1
Grade 2
Grade 3
Genome_instability_and_Mutation
Cell Neighbor Heterogeneity
Cancer Stage
Grade 1
Grade 2
Grade 3
Genome_instability_and_Mutation
Cell Social Entropy
Cancer Stage
Grade 1
Grade 2
Grade 3
Genome_instability_and_Mutation
Cell Social Heterogeneity
Cancer Stage
Grade 1
Grade 2
Grade 3

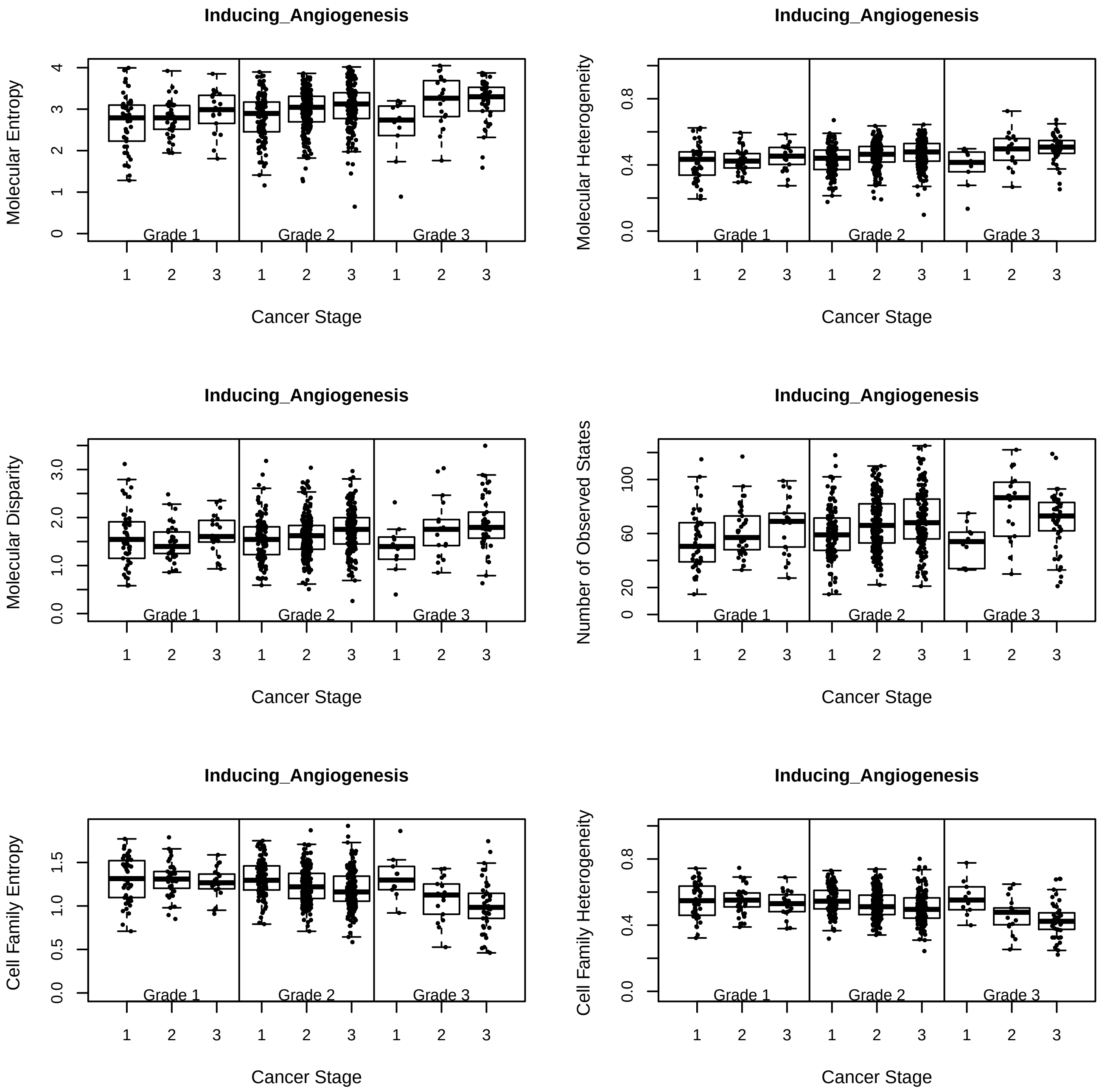
Inducing_Angiogenesis
Molecular Entropy
Inducing_Angiogenesis
Molecular Heterogeneity
Inducing_Angiogenesis
Molecular Disparity
Inducing_Angiogenesis
Number of Observed States
Inducing_Angiogenesis
Cell Family Entropy
Inducing_Angiogenesis
Cell Family Heterogeneity
Grade 1
Grade 2
Grade 3
Cancer Stage

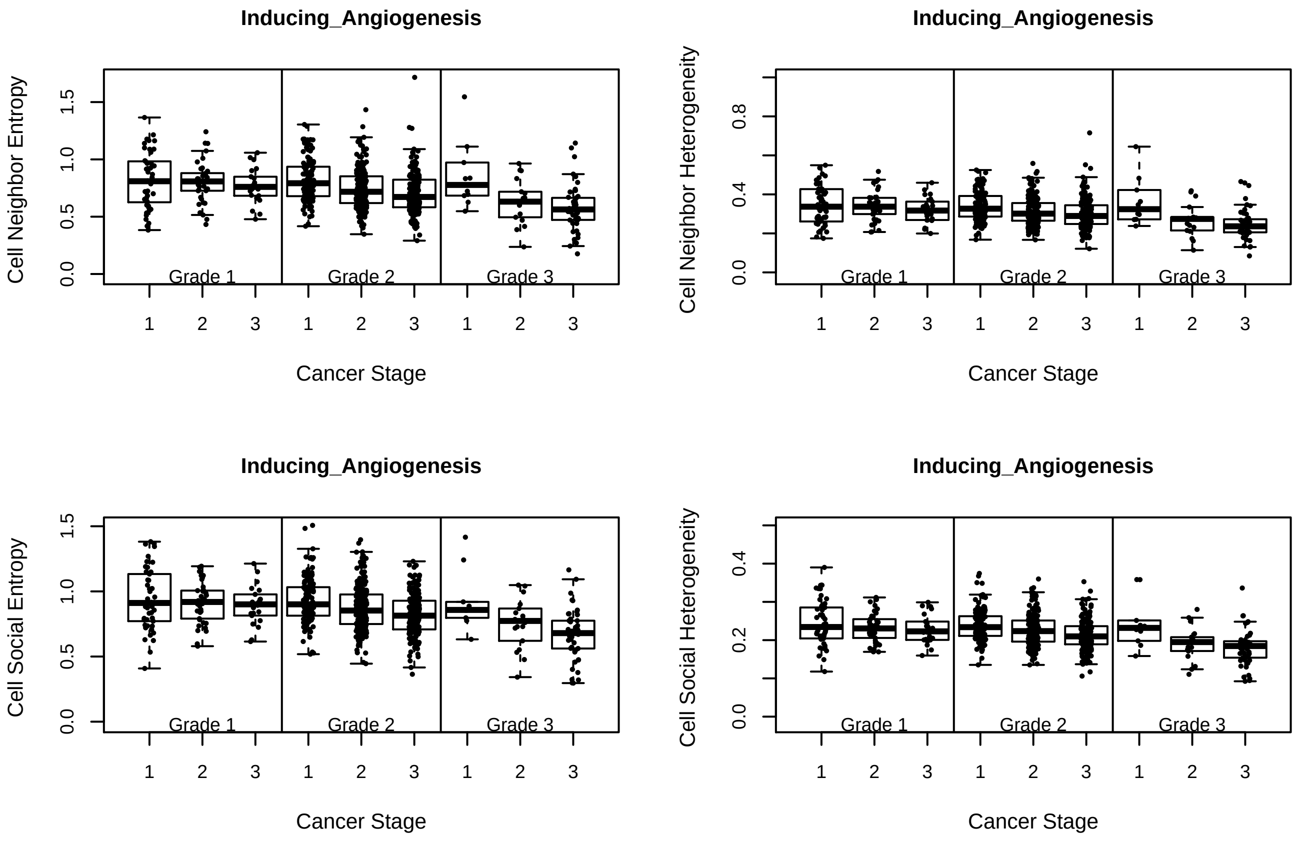

Inducing_Angiogenesis
Cell Neighbor Entropy
0.0
0.5
1.0
1.5
Grade 1
Grade 2
Grade 3
1 2 3 1 2 3 1 2 3
Cancer Stage
Inducing_Angiogenesis
Cell Neighbor Heterogeneity
0.0
0.4
0.8
Grade 1
Grade 2
Grade 3
1 2 3 1 2 3 1 2 3
Cancer Stage
Inducing_Angiogenesis
Cell Social Entropy
0.0
0.5
1.0
1.5
Grade 1
Grade 2
Grade 3
1 2 3 1 2 3 1 2 3
Cancer Stage
Inducing_Angiogenesis
Cell Social Heterogeneity
0.0
0.2
0.4
Grade 1
Grade 2
Grade 3
1 2 3 1 2 3 1 2 3
Cancer Stage

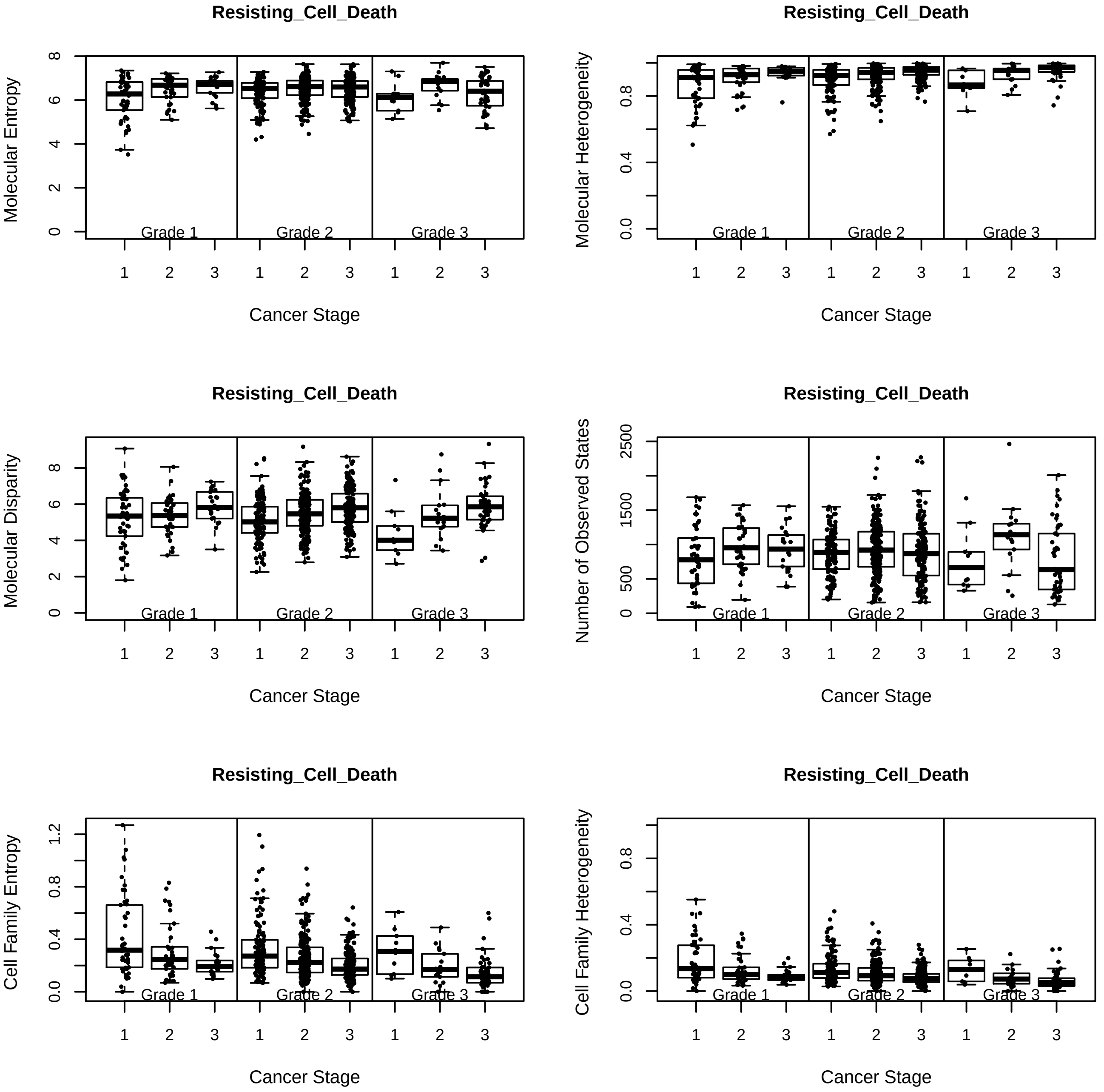
Resisting_Cell_Death
Molecular Entropy
Molecular Heterogeneity
Molecular Disparity
Number of Observed States
Cell Family Entropy
Cell Family Heterogeneity
Grade 1
Grade 2
Grade 3
Cancer Stage

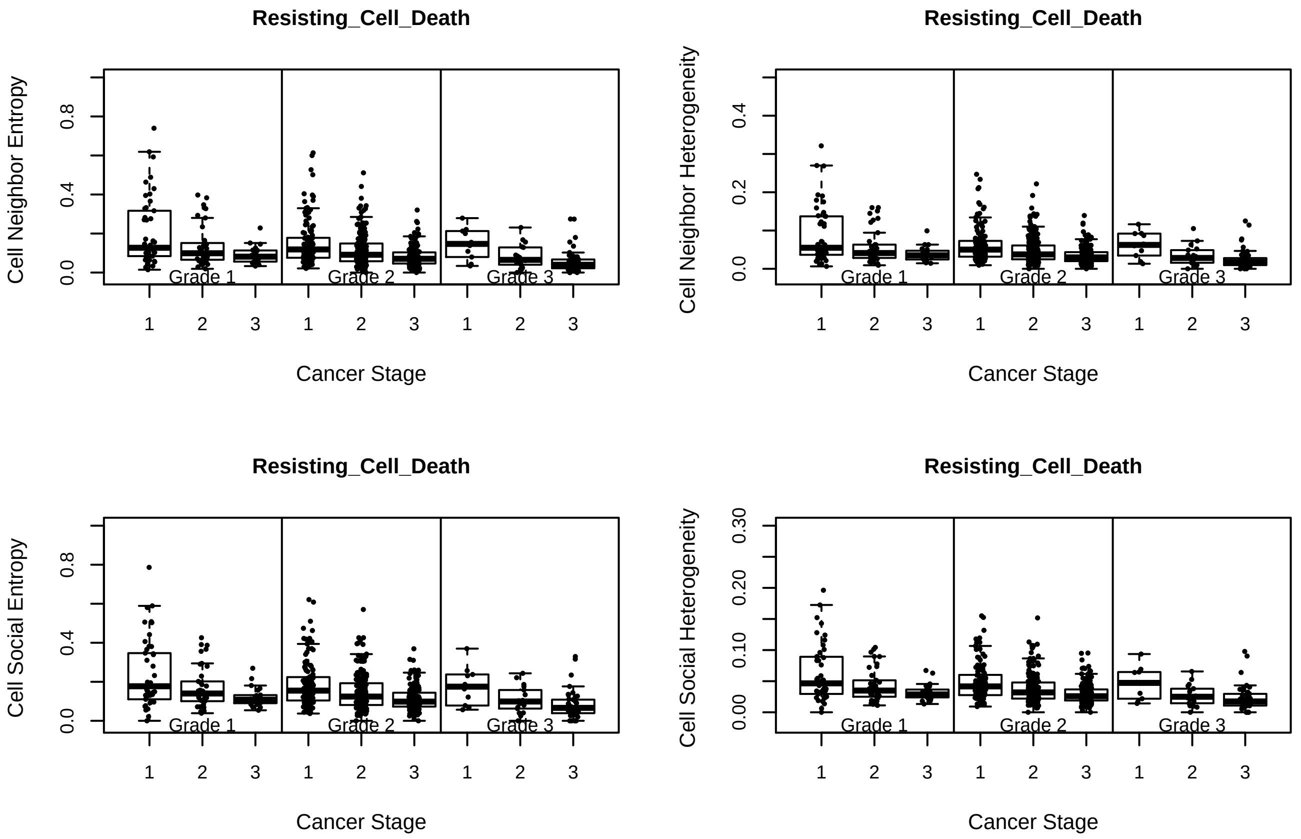
Resisting_Cell_Death
Cell Neighbor Entropy
0.0
0.4
0.8
Grade 1
Grade 2
Grade 3
1
2
3
Cancer Stage
Resisting_Cell_Death
Cell Neighbor Heterogeneity
0.0
0.2
0.4
Grade 1
Grade 2
Grade 3
1
2
3
Cancer Stage
Resisting_Cell_Death
Cell Social Entropy
0.0
0.4
0.8
Grade 1
Grade 2
Grade 3
1
2
3
Cancer Stage
Resisting_Cell_Death
Cell Social Heterogeneity
0.00
0.10
0.20
0.30
Grade 1
Grade 2
Grade 3
1
2
3
Cancer Stage

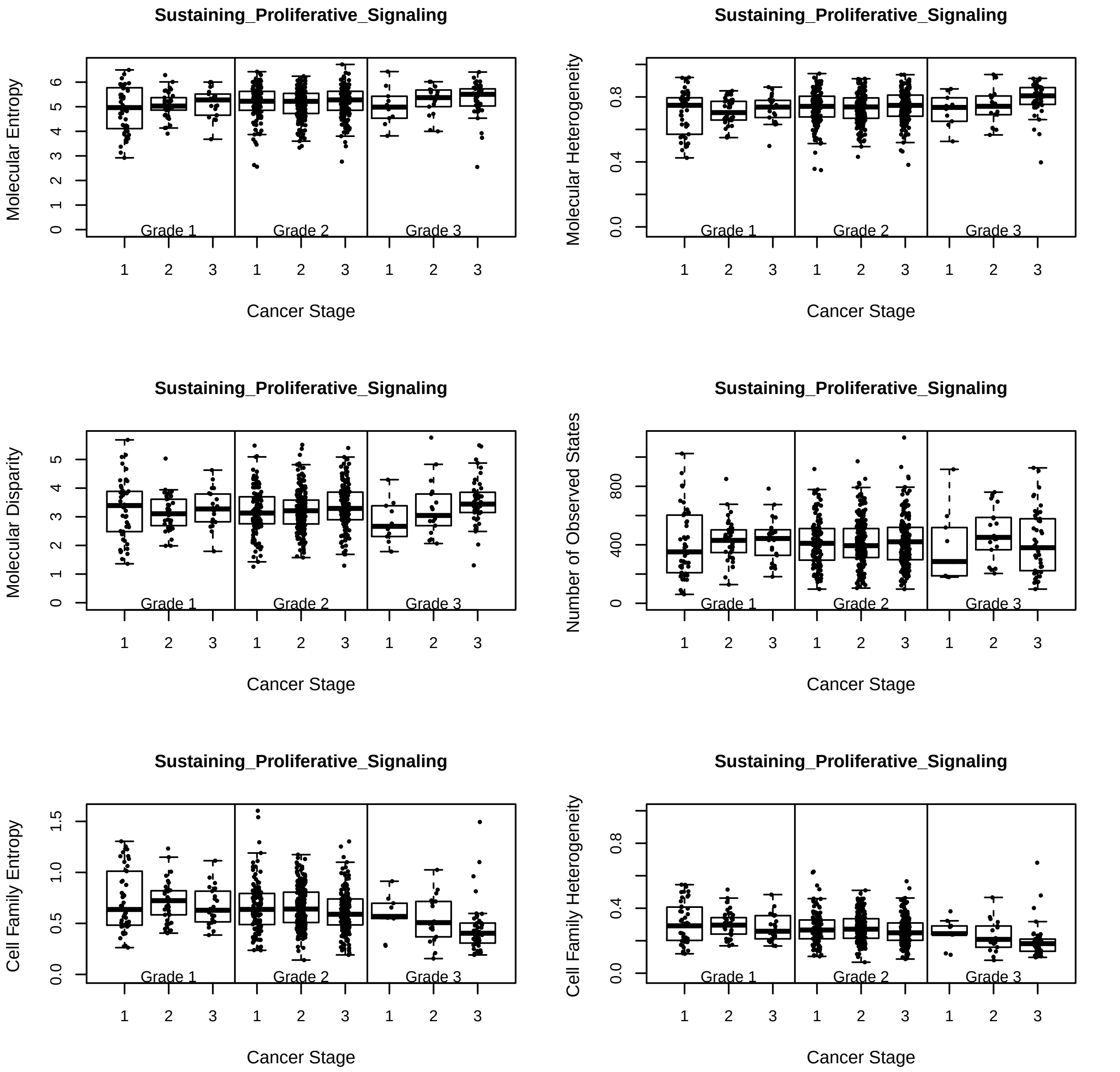
Sustaining_Proliferative_Signaling
Molecular Entropy
Grade 1
Grade 2
Grade 3
Cancer Stage
Sustaining_Proliferative_Signaling
Molecular Heterogeneity
Grade 1
Grade 2
Grade 3
Cancer Stage
Sustaining_Proliferative_Signaling
Molecular Disparity
Grade 1
Grade 2
Grade 3
Cancer Stage
Sustaining_Proliferative_Signaling
Number of Observed States
Grade 1
Grade 2
Grade 3
Cancer Stage
Sustaining_Proliferative_Signaling
Cell Family Entropy
Grade 1
Grade 2
Grade 3
Cancer Stage
Sustaining_Proliferative_Signaling
Cell Family Heterogeneity
Grade 1
Grade 2
Grade 3
Cancer Stage

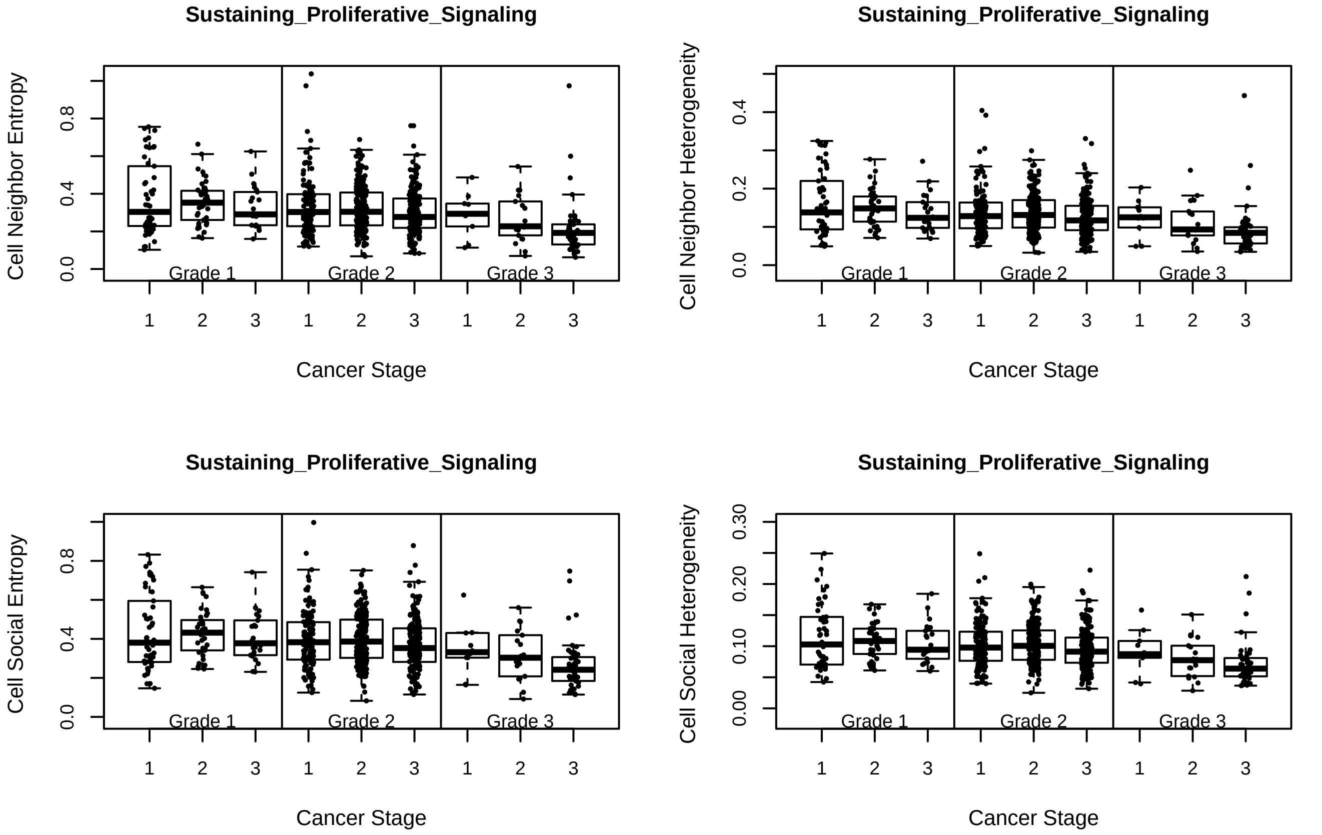
Sustaining_Proliferative_Signaling
Cell Neighbor Entropy
Cell Neighbor Heterogeneity
Cell Social Entropy
Cell Social Heterogeneity
Grade 1
Grade 2
Grade 3
Cancer Stage

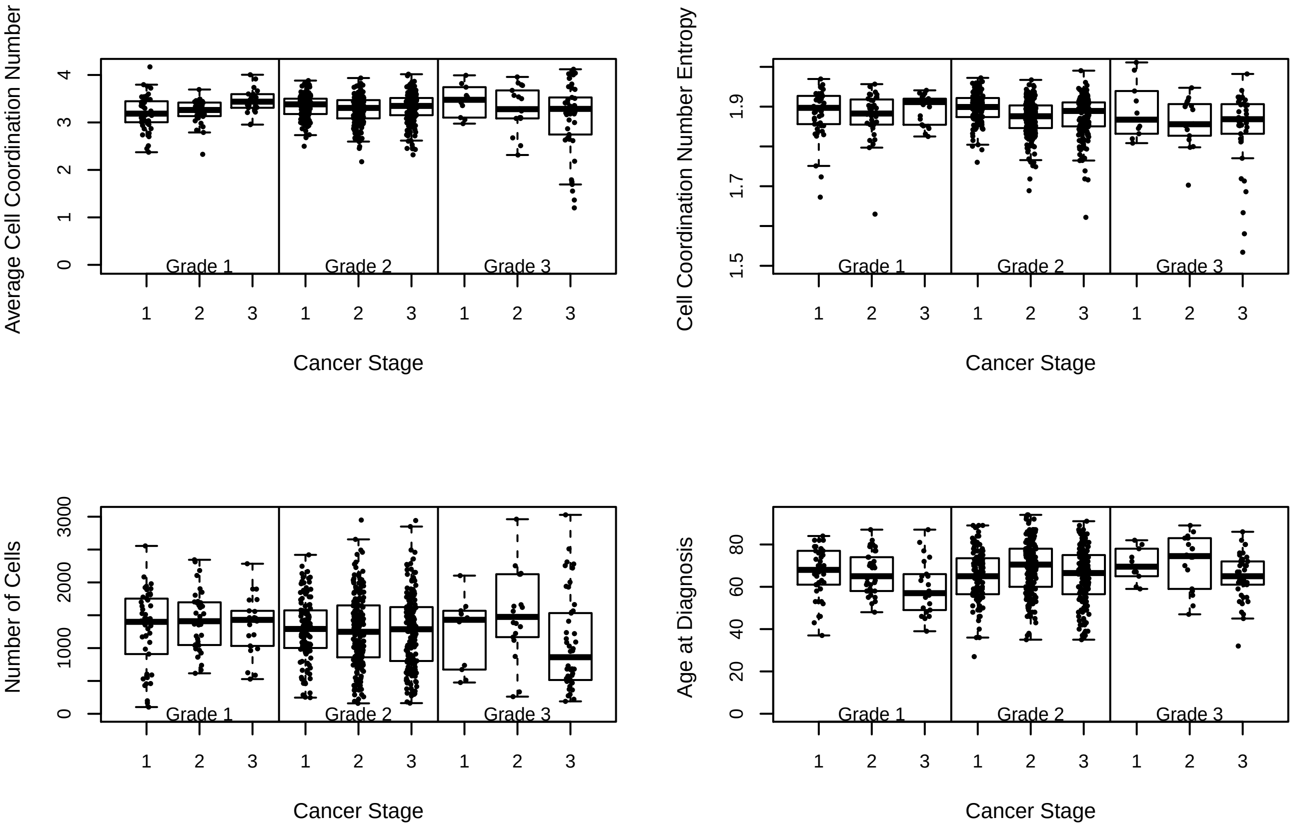
Average Cell Coordination Number
Cell Coordination Number Entropy
Number of Cells
Age at Diagnosis
Grade 1
Grade 2
Grade 3
Cancer Stage
0
1
2
3
4
1.5
1.7
1.9
1000
2000
3000
20
40
60
80

Section 4

Plots with molecular disparity, cell family, cell neighbor, and cell social heterogeneity versus molecular heterogeneity computed across 7 gene sets corresponding to cancer hallmarks and the AKT pathway. The values are colored by cancer stage.

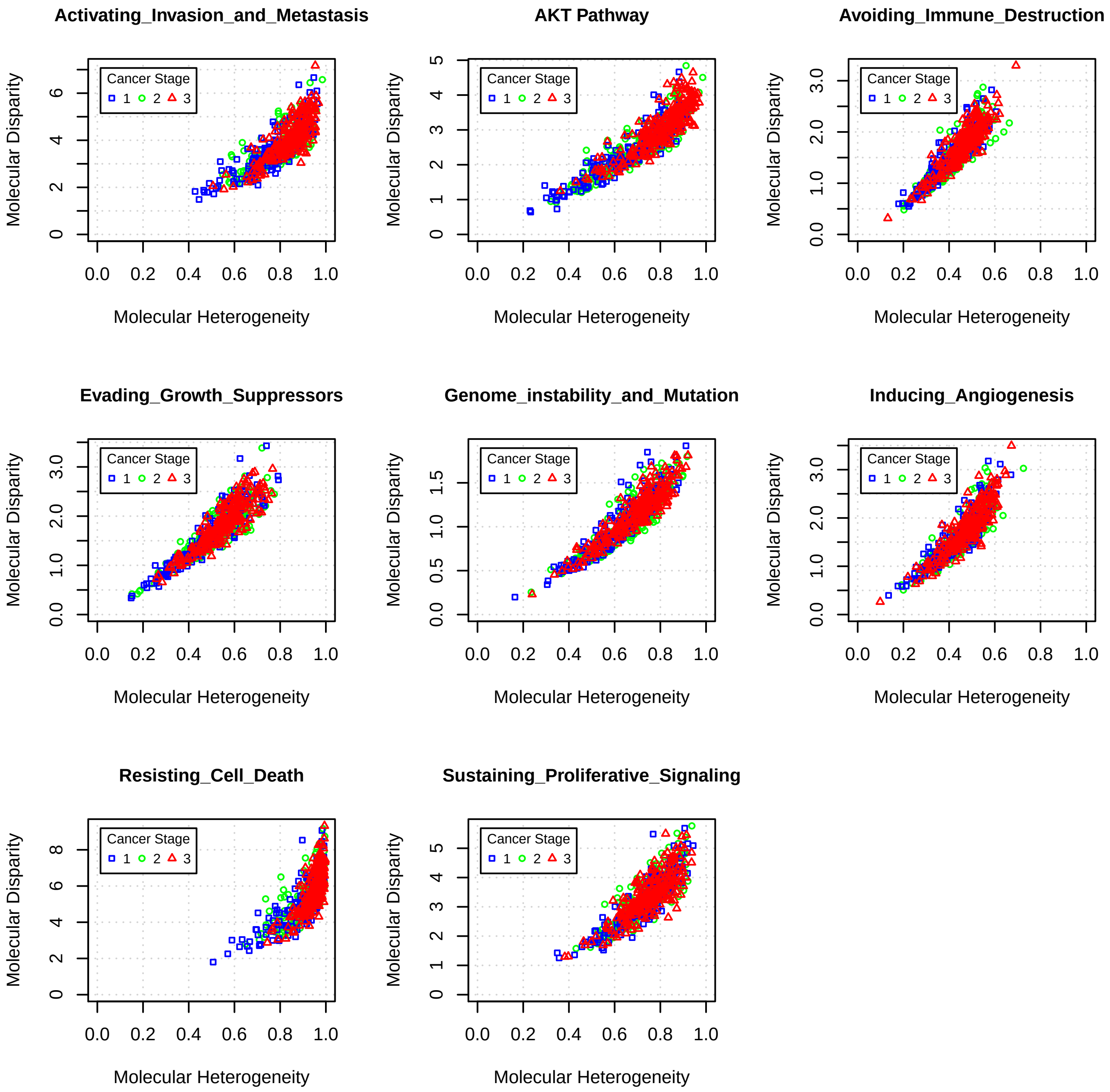
Activating_Invasion_and_Metastasis
AKT Pathway
Avoiding_Immune_Destruction
Evading_Growth_Suppressors
Genome_instability_and_Mutation
Inducing_Angiogenesis
Resisting_Cell_Death
Sustaining_Proliferative_Signaling
Cancer Stage
1 2 3
Molecular Disparity
Molecular Heterogeneity
0.0 0.2 0.4 0.6 0.8 1.0

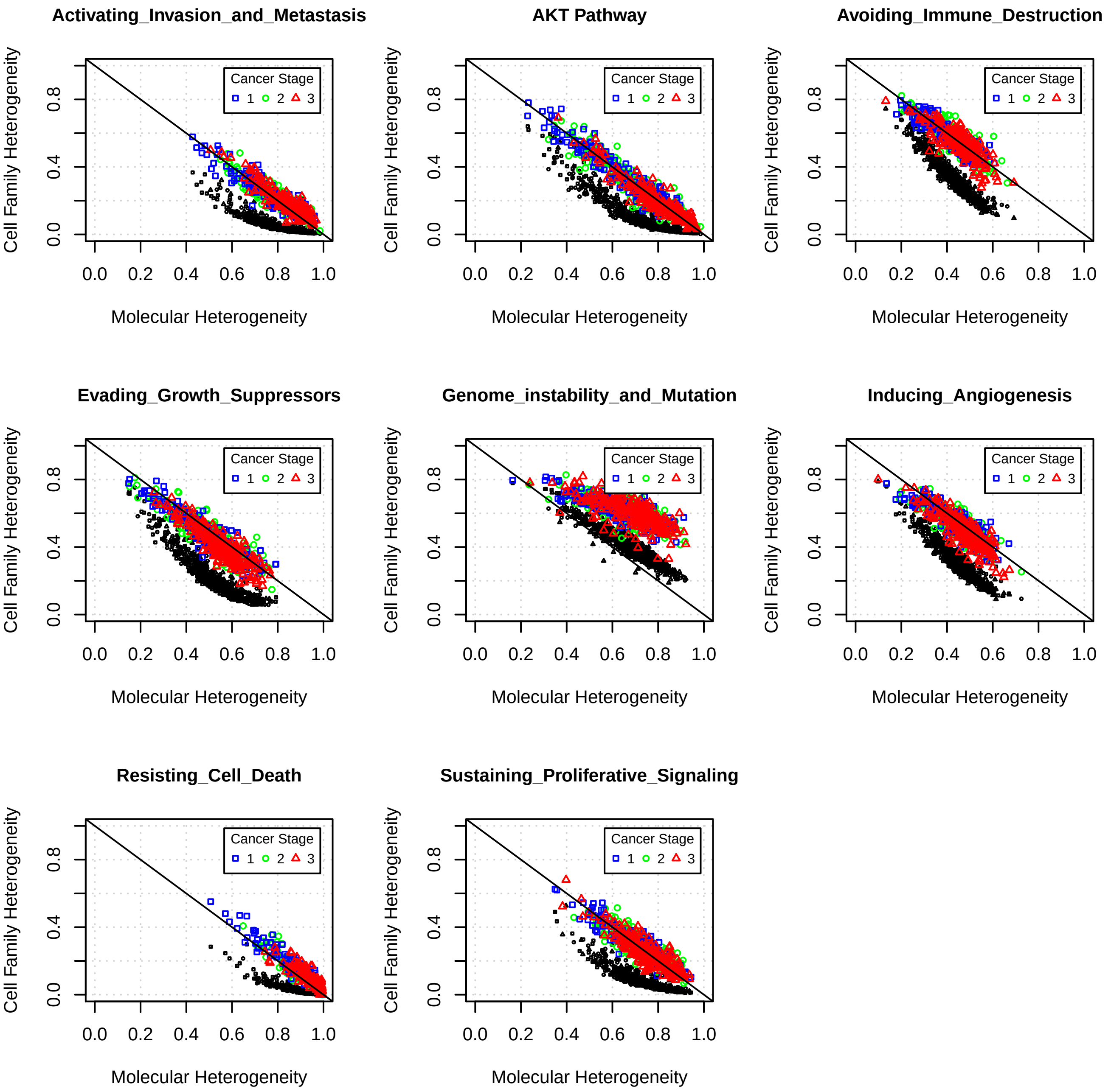

Activating_Invasion_and_Metastasis
AKT Pathway
Avoiding_Immune_Destruction
Evading_Growth_Suppressors
Genome_instability_and_Mutation
Inducing_Angiogenesis
Resisting_Cell_Death
Sustaining_Proliferative_Signaling
Cancer Stage
1 2 3
Cell Family Heterogeneity
Molecular Heterogeneity
0.0 0.4 0.8
0.0 0.2 0.4 0.6 0.8 1.0

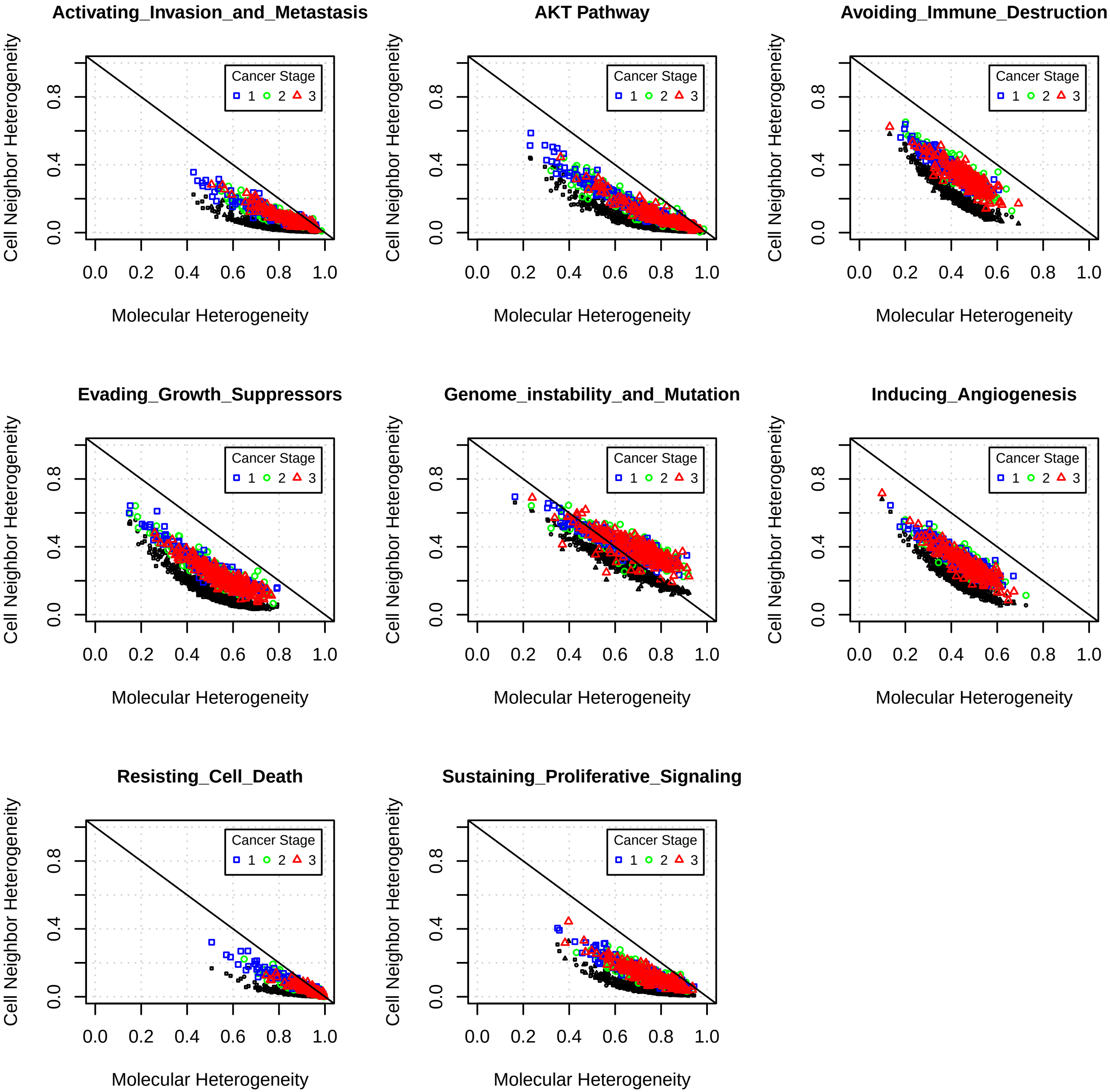
Activating_Invasion_and_Metastasis
AKT Pathway
Avoiding_Immune_Destruction
Evading_Growth_Suppressors
Genome_instability_and_Mutation
Inducing_Angiogenesis
Resisting_Cell_Death
Sustaining_Proliferative_Signaling
Cancer Stage
1 2 3
Cell Neighbor Heterogeneity
Molecular Heterogeneity
0.0
0.4
0.8
0.0 0.2 0.4 0.6 0.8 1.0

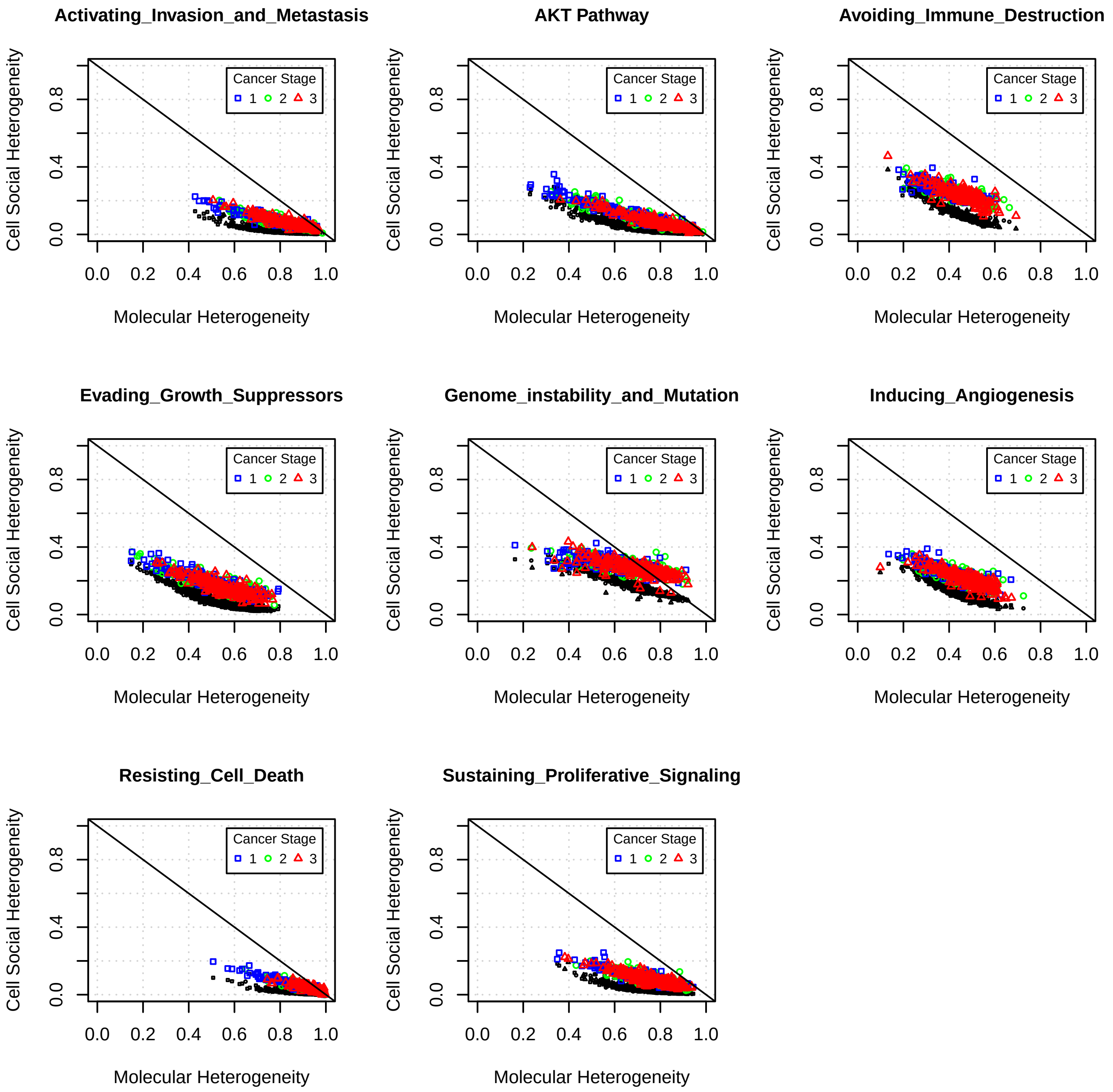
Activating_Invasion_and_Metastasis
AKT Pathway
Avoiding_Immune_Destruction
Evading_Growth_Suppressors
Genome_instability_and_Mutation
Inducing_Angiogenesis
Resisting_Cell_Death
Sustaining_Proliferative_Signaling
Cancer Stage
1 2 3
Cell Social Heterogeneity
Molecular Heterogeneity
0.0 0.4 0.8
0.0 0.2 0.4 0.6 0.8 1.0

Section 5

Plots with molecular disparity, cell family, cell neighbor, and cell social heterogeneity versus molecular heterogeneity computed across 7 gene sets corresponding to cancer hallmarks and the AKT pathway. The values are colored by cancer grade.

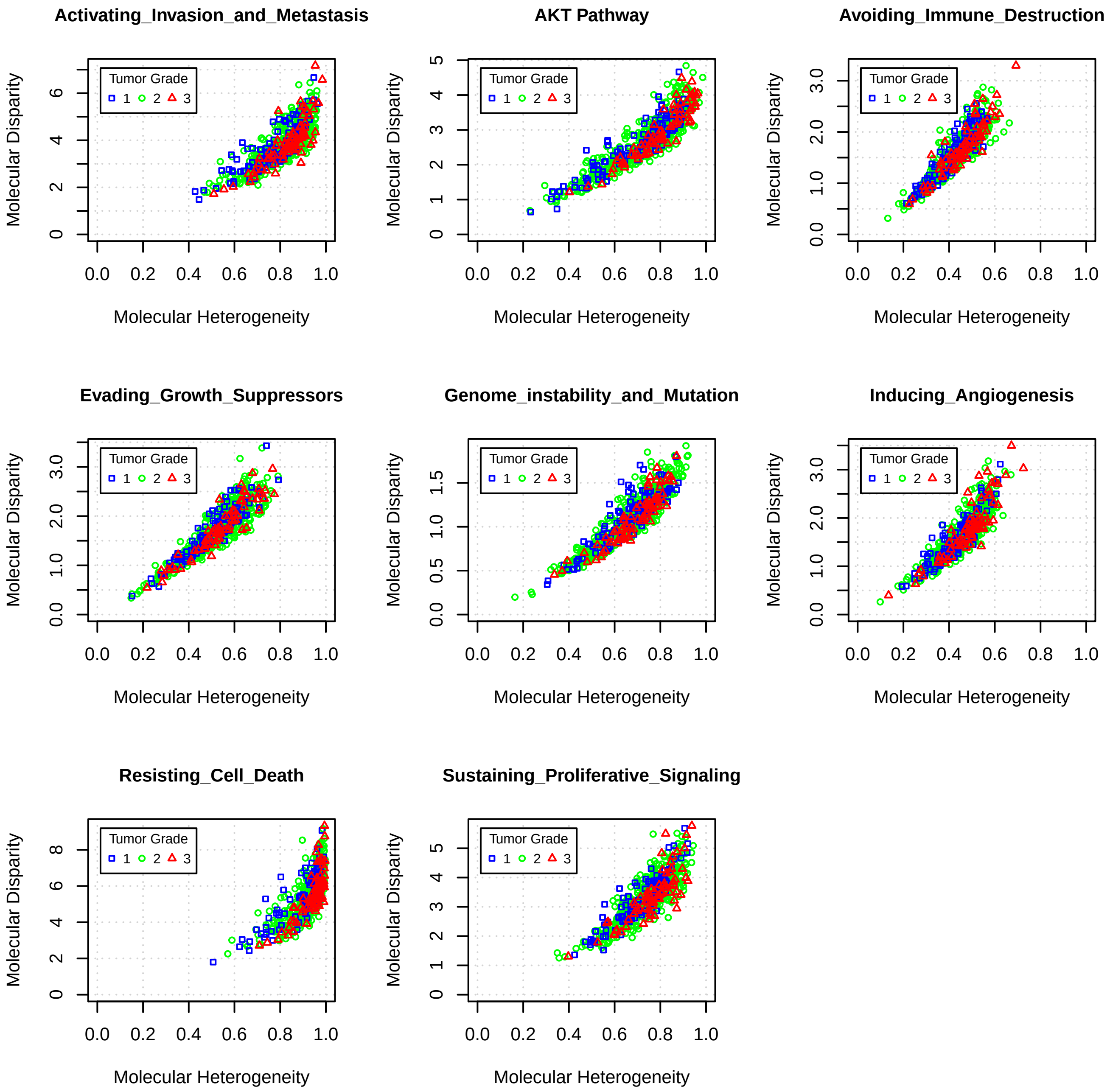

Activating_Invasion_and_Metastasis
AKT Pathway
Avoiding_Immune_Destruction
Evading_Growth_Suppressors
Genome_instability_and_Mutation
Inducing_Angiogenesis
Resisting_Cell_Death
Sustaining_Proliferative_Signaling
Tumor Grade
1 2 3
Molecular Disparity
Molecular Heterogeneity
0.0 0.2 0.4 0.6 0.8 1.0

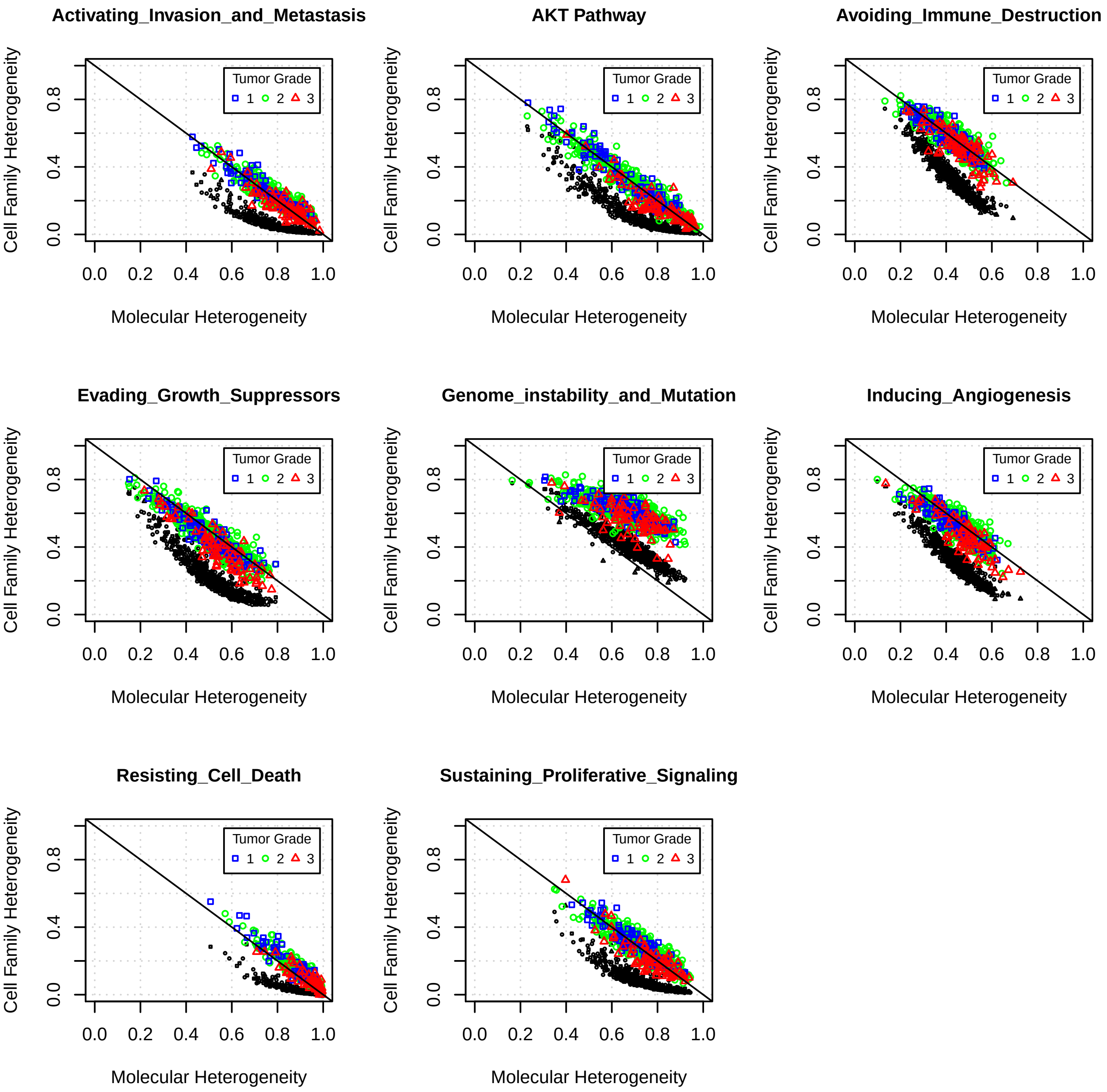

Activating_Invasion_and_Metastasis
AKT Pathway
Avoiding_Immune_Destruction
Evading_Growth_Suppressors
Genome_instability_and_Mutation
Inducing_Angiogenesis
Resisting_Cell_Death
Sustaining_Proliferative_Signaling
Tumor Grade
1 2 3
Cell Family Heterogeneity
Molecular Heterogeneity
0.0 0.4 0.8
0.0 0.2 0.4 0.6 0.8 1.0

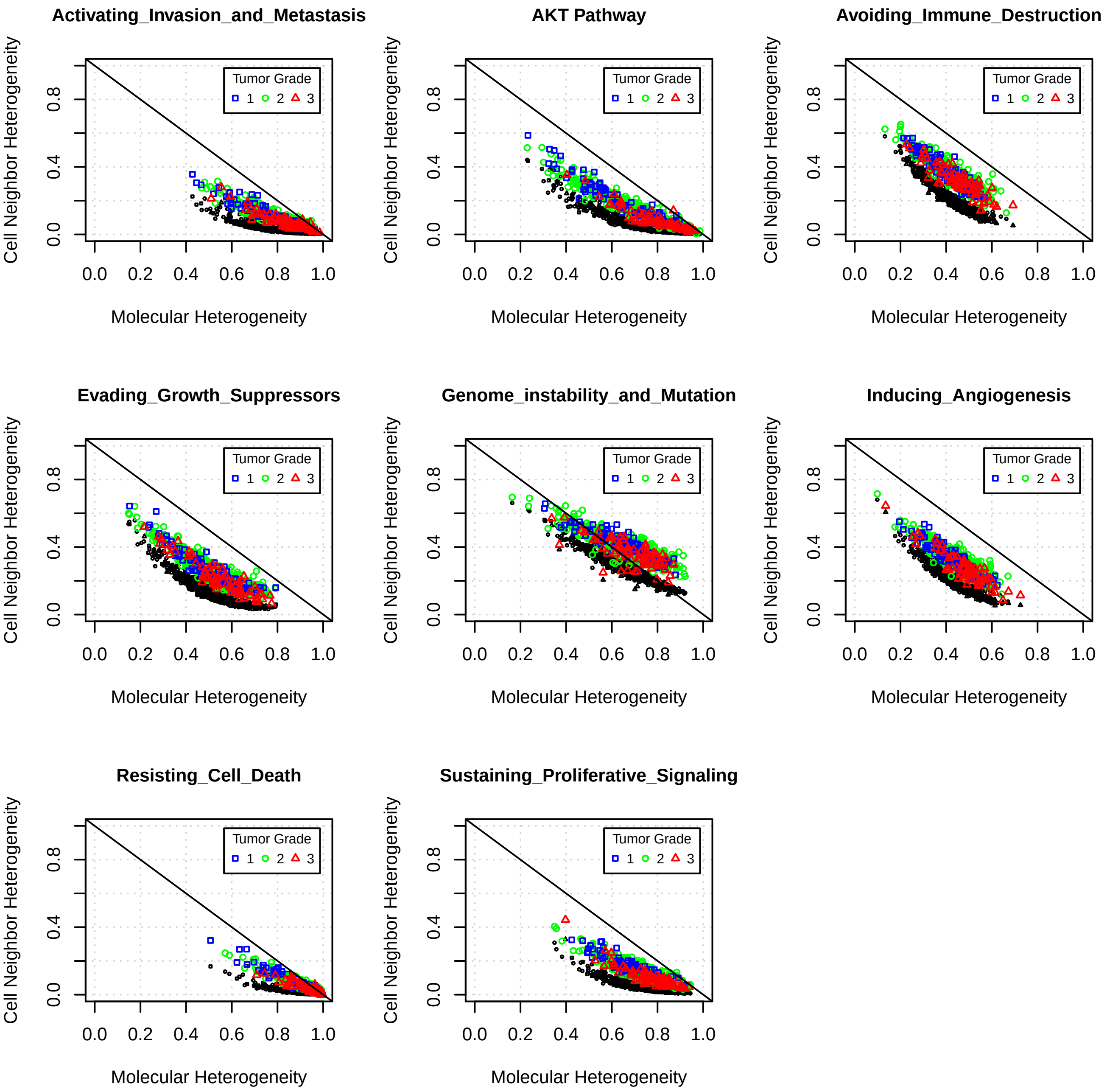

Activating_Invasion_and_Metastasis
AKT Pathway
Avoiding_Immune_Destruction
Evading_Growth_Suppressors
Genome_instability_and_Mutation
Inducing_Angiogenesis
Resisting_Cell_Death
Sustaining_Proliferative_Signaling
Tumor Grade
1 2 3
Cell Neighbor Heterogeneity
Molecular Heterogeneity
0.0 0.2 0.4 0.6 0.8 1.0
0.0 0.4 0.8

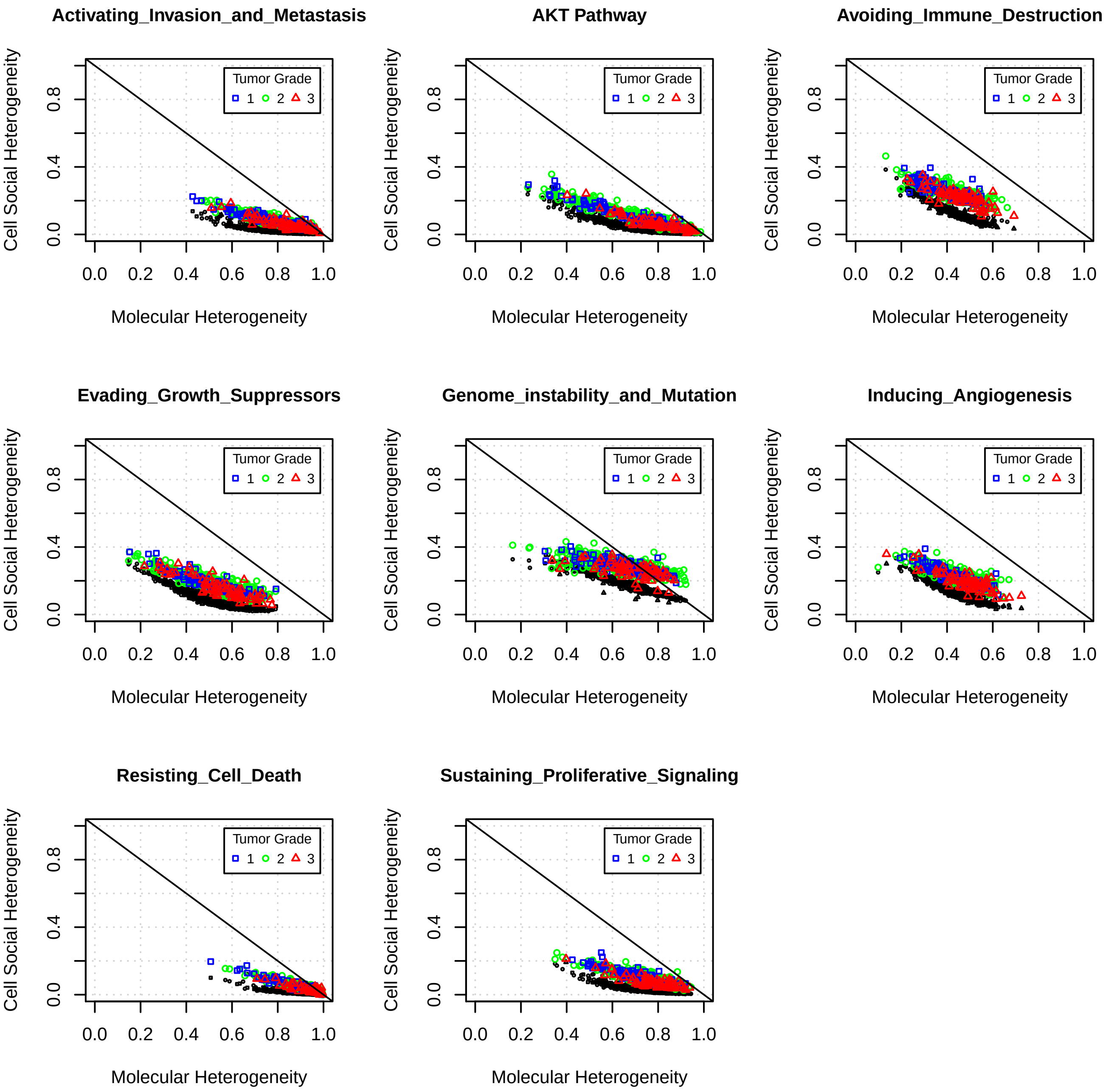
Activating_Invasion_and_Metastasis
AKT Pathway
Avoiding_Immune_Destruction
Evading_Growth_Suppressors
Genome_instability_and_Mutation
Inducing_Angiogenesis
Resisting_Cell_Death
Sustaining_Proliferative_Signaling
Tumor Grade
1 2 3
Cell Social Heterogeneity
Molecular Heterogeneity
0.0 0.4 0.8
0.0 0.2 0.4 0.6 0.8 1.0

Section 6

Box charts with diversity metrics by chemotherapy treatment and recurrence calculated based on 7 gene sets corresponding to cancer hallmarks and the AKT pathway. Box charts of average cell coordination number, number of cells and age at diagnosis broken down by treatment and recurrence are also included.

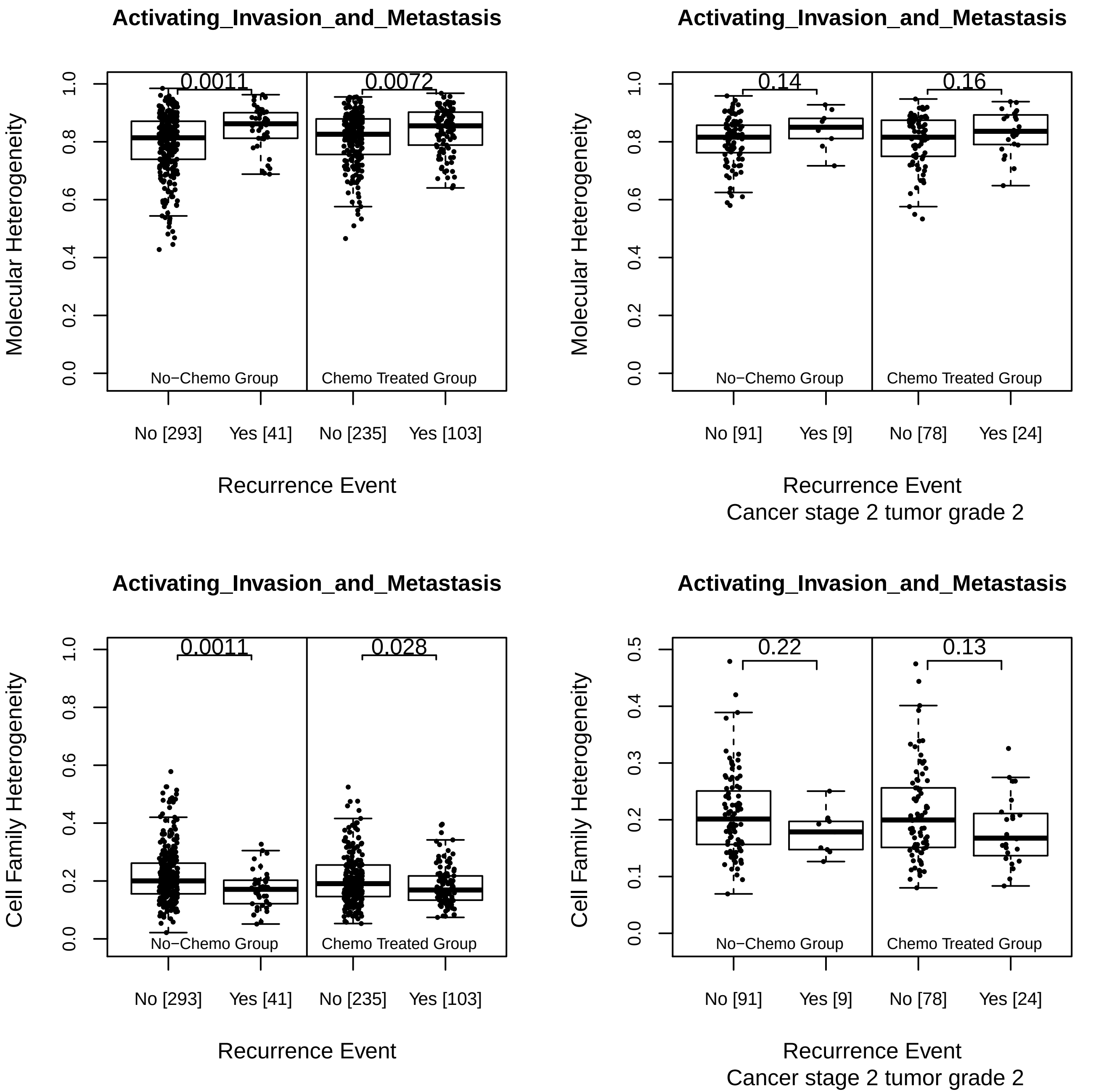
Activating_Invasion_and_Metastasis
Molecular Heterogeneity
0.0011
0.0072
No−Chemo Group
Chemo Treated Group
No [293]
Yes [41]
No [235]
Yes [103]
Recurrence Event
Activating_Invasion_and_Metastasis
Molecular Heterogeneity
0.14
0.16
No−Chemo Group
Chemo Treated Group
No [91]
Yes [9]
No [78]
Yes [24]
Recurrence Event
Cancer stage 2 tumor grade 2
Activating_Invasion_and_Metastasis
Cell Family Heterogeneity
0.0011
0.028
No−Chemo Group
Chemo Treated Group
No [293]
Yes [41]
No [235]
Yes [103]
Recurrence Event
Activating_Invasion_and_Metastasis
Cell Family Heterogeneity
0.22
0.13
No−Chemo Group
Chemo Treated Group
No [91]
Yes [9]
No [78]
Yes [24]
Recurrence Event
Cancer stage 2 tumor grade 2

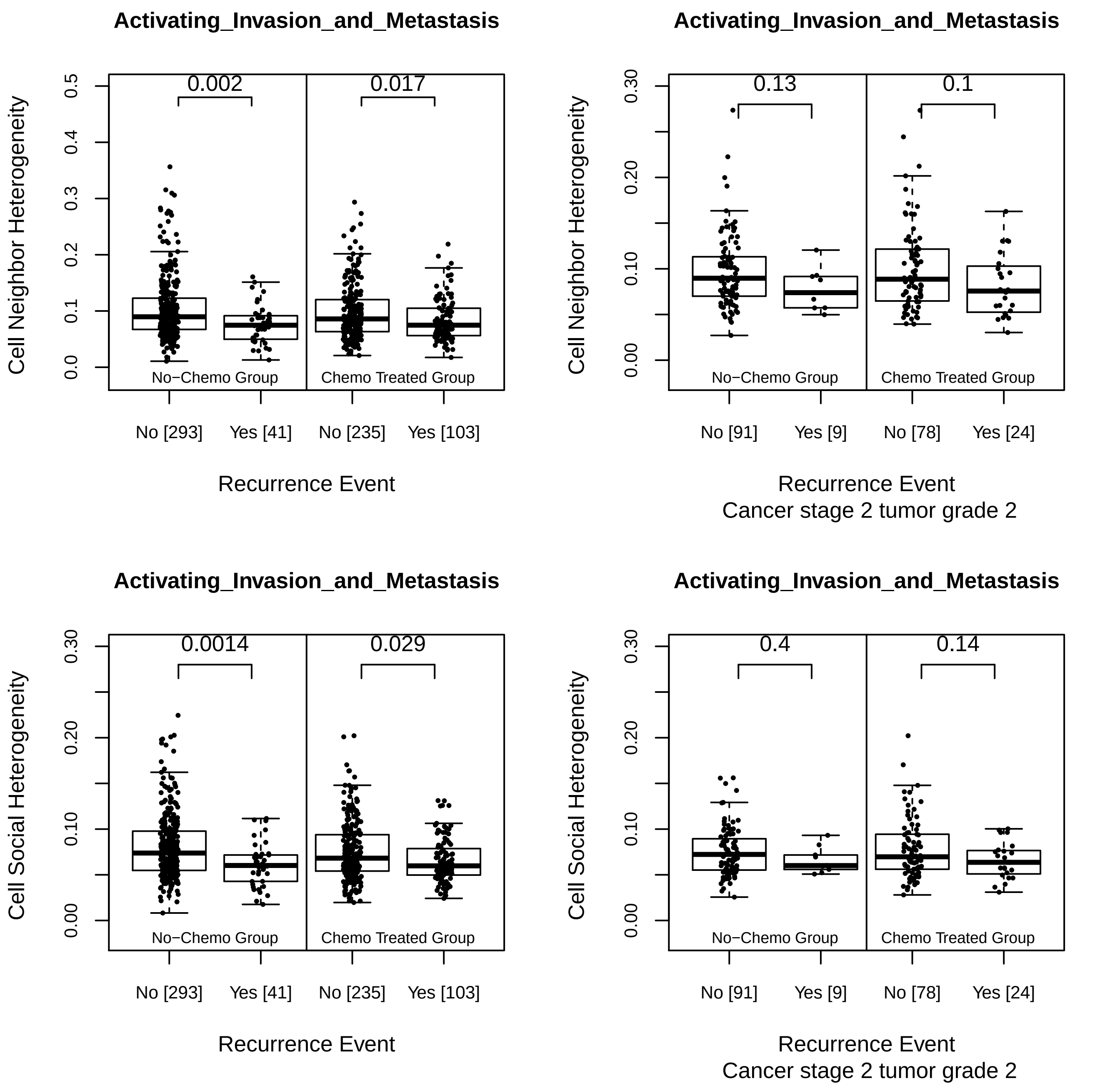
Activating_Invasion_and_Metastasis
Cell Neighbor Heterogeneity
0.002
0.017
No−Chemo Group
Chemo Treated Group
No [293]
Yes [41]
No [235]
Yes [103]
Recurrence Event
Activating_Invasion_and_Metastasis
Cell Neighbor Heterogeneity
0.13
0.1
No−Chemo Group
Chemo Treated Group
No [91]
Yes [9]
No [78]
Yes [24]
Recurrence Event
Cancer stage 2 tumor grade 2
Activating_Invasion_and_Metastasis
Cell Social Heterogeneity
0.0014
0.029
No−Chemo Group
Chemo Treated Group
No [293]
Yes [41]
No [235]
Yes [103]
Recurrence Event
Activating_Invasion_and_Metastasis
Cell Social Heterogeneity
0.4
0.14
No−Chemo Group
Chemo Treated Group
No [91]
Yes [9]
No [78]
Yes [24]
Recurrence Event
Cancer stage 2 tumor grade 2

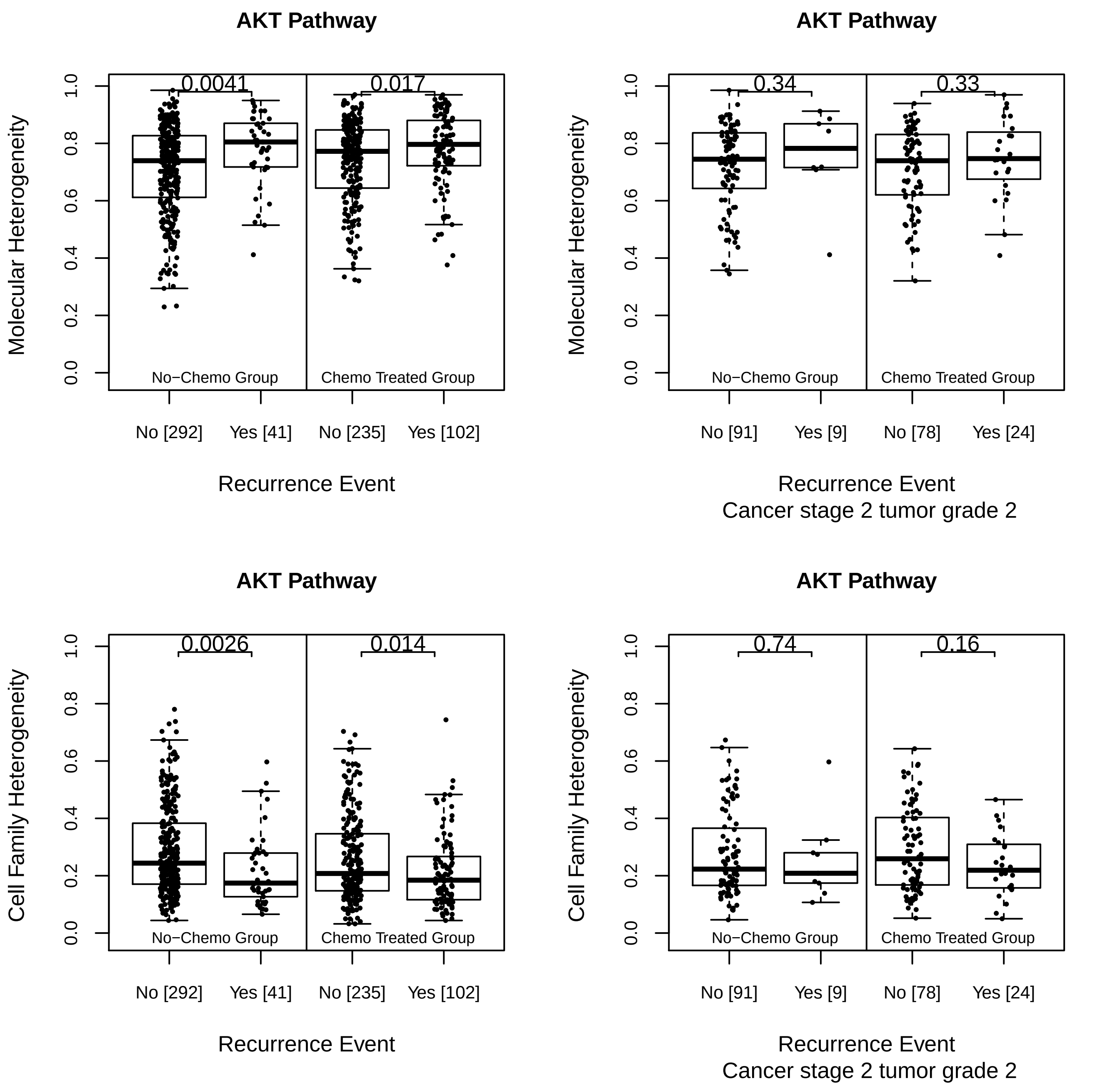
AKT Pathway
Molecular Heterogeneity
0.0041
0.017
No−Chemo Group
Chemo Treated Group
No [292]
Yes [41]
No [235]
Yes [102]
Recurrence Event
AKT Pathway
Molecular Heterogeneity
0.34
0.33
No−Chemo Group
Chemo Treated Group
No [91]
Yes [9]
No [78]
Yes [24]
Recurrence Event
Cancer stage 2 tumor grade 2
AKT Pathway
Cell Family Heterogeneity
0.0026
0.014
No−Chemo Group
Chemo Treated Group
No [292]
Yes [41]
No [235]
Yes [102]
Recurrence Event
AKT Pathway
Cell Family Heterogeneity
0.74
0.16
No−Chemo Group
Chemo Treated Group
No [91]
Yes [9]
No [78]
Yes [24]
Recurrence Event
Cancer stage 2 tumor grade 2

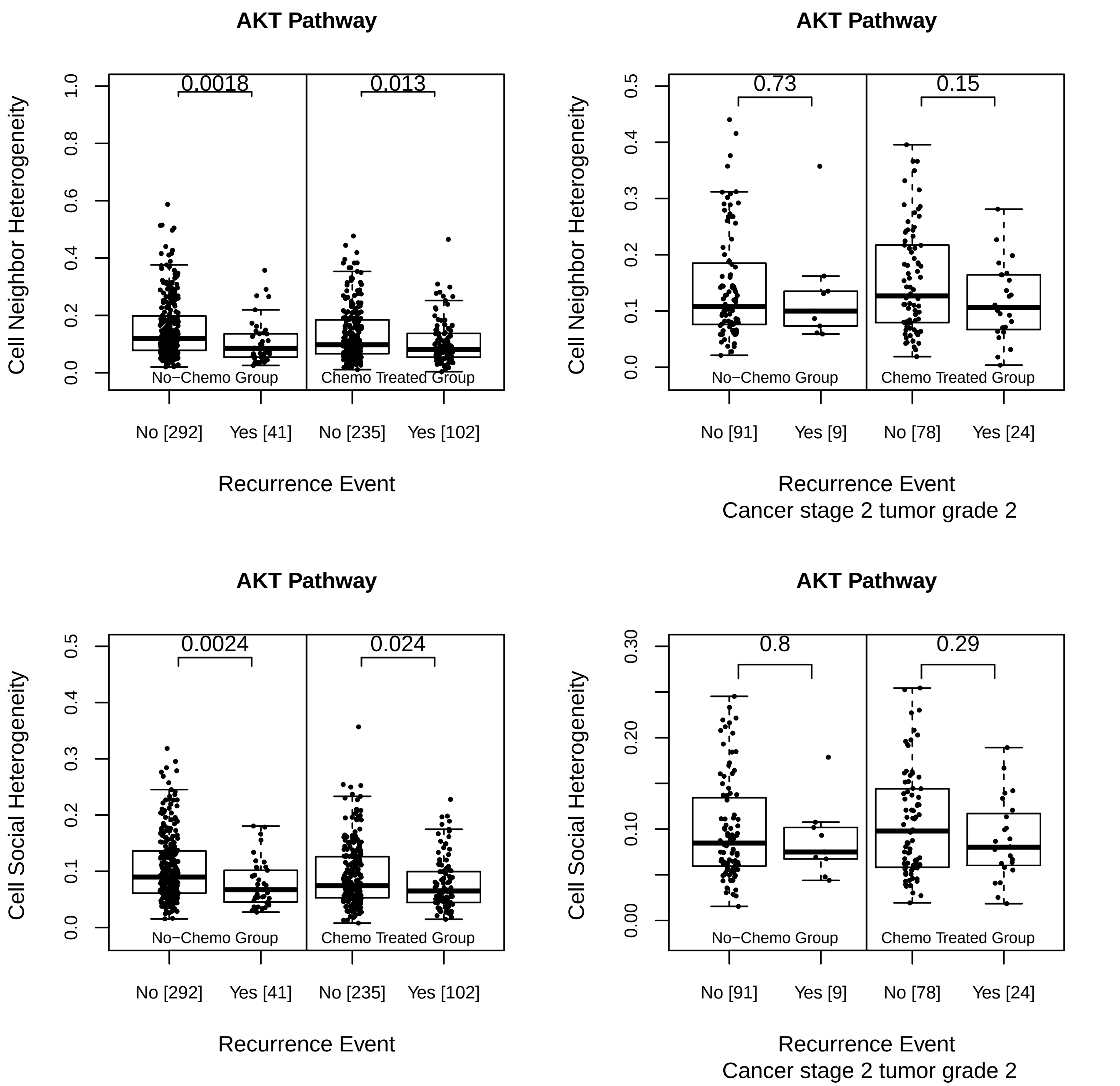
AKT Pathway
Cell Neighbor Heterogeneity
0.0018
0.013
No−Chemo Group
Chemo Treated Group
No [292]
Yes [41]
No [235]
Yes [102]
Recurrence Event
AKT Pathway
Cell Neighbor Heterogeneity
0.73
0.15
No−Chemo Group
Chemo Treated Group
No [91]
Yes [9]
No [78]
Yes [24]
Recurrence Event
Cancer stage 2 tumor grade 2
AKT Pathway
Cell Social Heterogeneity
0.0024
0.024
No−Chemo Group
Chemo Treated Group
No [292]
Yes [41]
No [235]
Yes [102]
Recurrence Event
AKT Pathway
Cell Social Heterogeneity
0.8
0.29
No−Chemo Group
Chemo Treated Group
No [91]
Yes [9]
No [78]
Yes [24]
Recurrence Event
Cancer stage 2 tumor grade 2

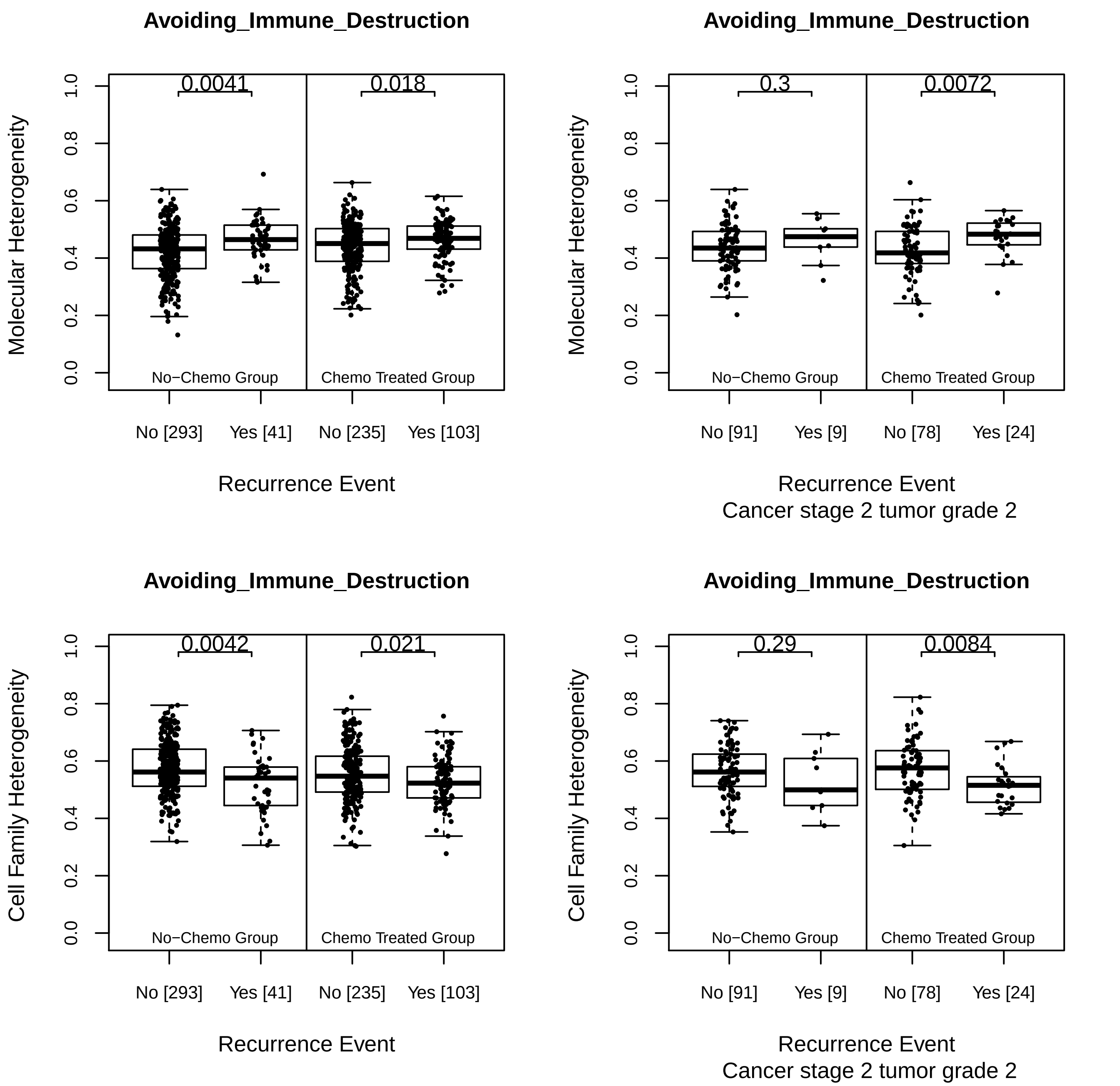

Avoiding_Immune_Destruction
Molecular Heterogeneity
0.0041
0.018
No−Chemo Group
Chemo Treated Group
No [293]
Yes [41]
No [235]
Yes [103]
Recurrence Event
Avoiding_Immune_Destruction
Molecular Heterogeneity
0.3
0.0072
No−Chemo Group
Chemo Treated Group
No [91]
Yes [9]
No [78]
Yes [24]
Recurrence Event
Cancer stage 2 tumor grade 2
Avoiding_Immune_Destruction
Cell Family Heterogeneity
0.0042
0.021
No−Chemo Group
Chemo Treated Group
No [293]
Yes [41]
No [235]
Yes [103]
Recurrence Event
Avoiding_Immune_Destruction
Cell Family Heterogeneity
0.29
0.0084
No−Chemo Group
Chemo Treated Group
No [91]
Yes [9]
No [78]
Yes [24]
Recurrence Event
Cancer stage 2 tumor grade 2

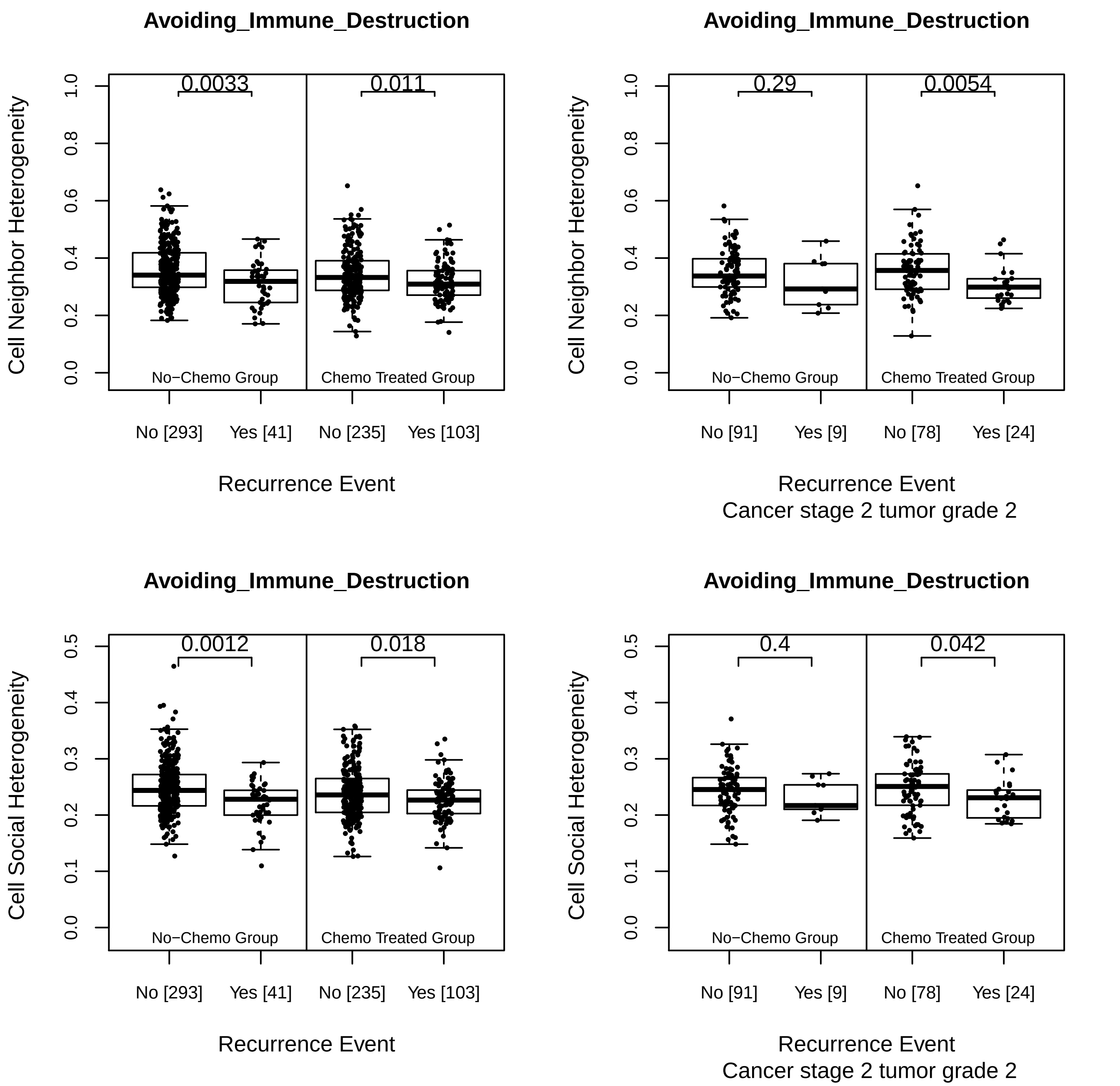

Avoiding_Immune_Destruction
Cell Neighbor Heterogeneity
0.0033
0.011
No−Chemo Group
Chemo Treated Group
No [293]
Yes [41]
No [235]
Yes [103]
Recurrence Event
Avoiding_Immune_Destruction
Cell Neighbor Heterogeneity
0.29
0.0054
No−Chemo Group
Chemo Treated Group
No [91]
Yes [9]
No [78]
Yes [24]
Recurrence Event
Cancer stage 2 tumor grade 2
Avoiding_Immune_Destruction
Cell Social Heterogeneity
0.0012
0.018
No−Chemo Group
Chemo Treated Group
No [293]
Yes [41]
No [235]
Yes [103]
Recurrence Event
Avoiding_Immune_Destruction
Cell Social Heterogeneity
0.4
0.042
No−Chemo Group
Chemo Treated Group
No [91]
Yes [9]
No [78]
Yes [24]
Recurrence Event
Cancer stage 2 tumor grade 2

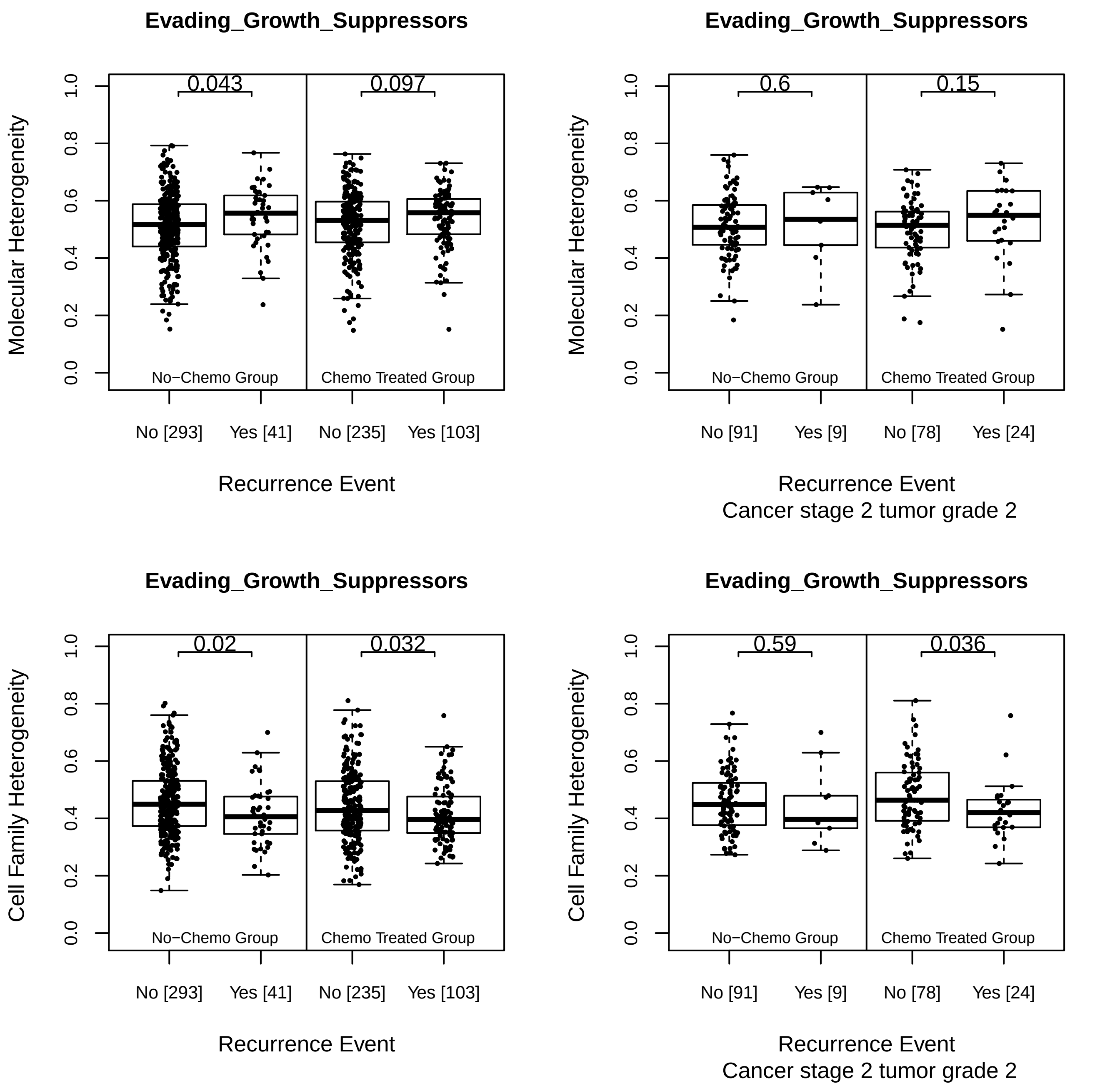

Evading_Growth_Suppressors
Molecular Heterogeneity
0.043
0.097
No−Chemo Group
Chemo Treated Group
No [293]
Yes [41]
No [235]
Yes [103]
Recurrence Event
Evading_Growth_Suppressors
Molecular Heterogeneity
0.6
0.15
No−Chemo Group
Chemo Treated Group
No [91]
Yes [9]
No [78]
Yes [24]
Recurrence Event
Cancer stage 2 tumor grade 2
Evading_Growth_Suppressors
Cell Family Heterogeneity
0.02
0.032
No−Chemo Group
Chemo Treated Group
No [293]
Yes [41]
No [235]
Yes [103]
Recurrence Event
Evading_Growth_Suppressors
Cell Family Heterogeneity
0.59
0.036
No−Chemo Group
Chemo Treated Group
No [91]
Yes [9]
No [78]
Yes [24]
Recurrence Event
Cancer stage 2 tumor grade 2

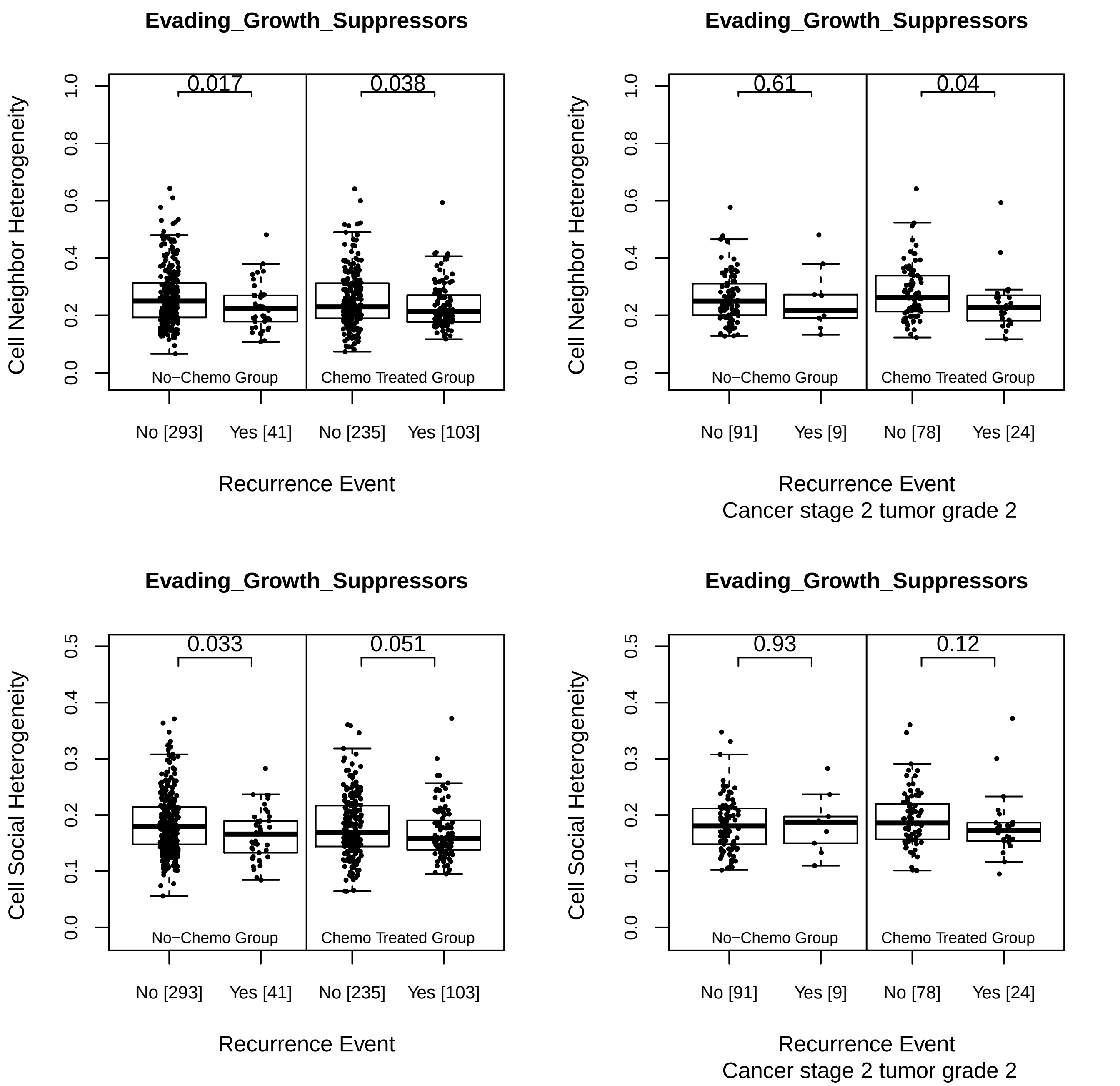
Evading_Growth_Suppressors
Cell Neighbor Heterogeneity
0.017
0.038
No−Chemo Group
Chemo Treated Group
No [293]
Yes [41]
No [235]
Yes [103]
Recurrence Event
Evading_Growth_Suppressors
Cell Neighbor Heterogeneity
0.61
0.04
No−Chemo Group
Chemo Treated Group
No [91]
Yes [9]
No [78]
Yes [24]
Recurrence Event
Cancer stage 2 tumor grade 2
Evading_Growth_Suppressors
Cell Social Heterogeneity
0.033
0.051
No−Chemo Group
Chemo Treated Group
No [293]
Yes [41]
No [235]
Yes [103]
Recurrence Event
Evading_Growth_Suppressors
Cell Social Heterogeneity
0.93
0.12
No−Chemo Group
Chemo Treated Group
No [91]
Yes [9]
No [78]
Yes [24]
Recurrence Event
Cancer stage 2 tumor grade 2

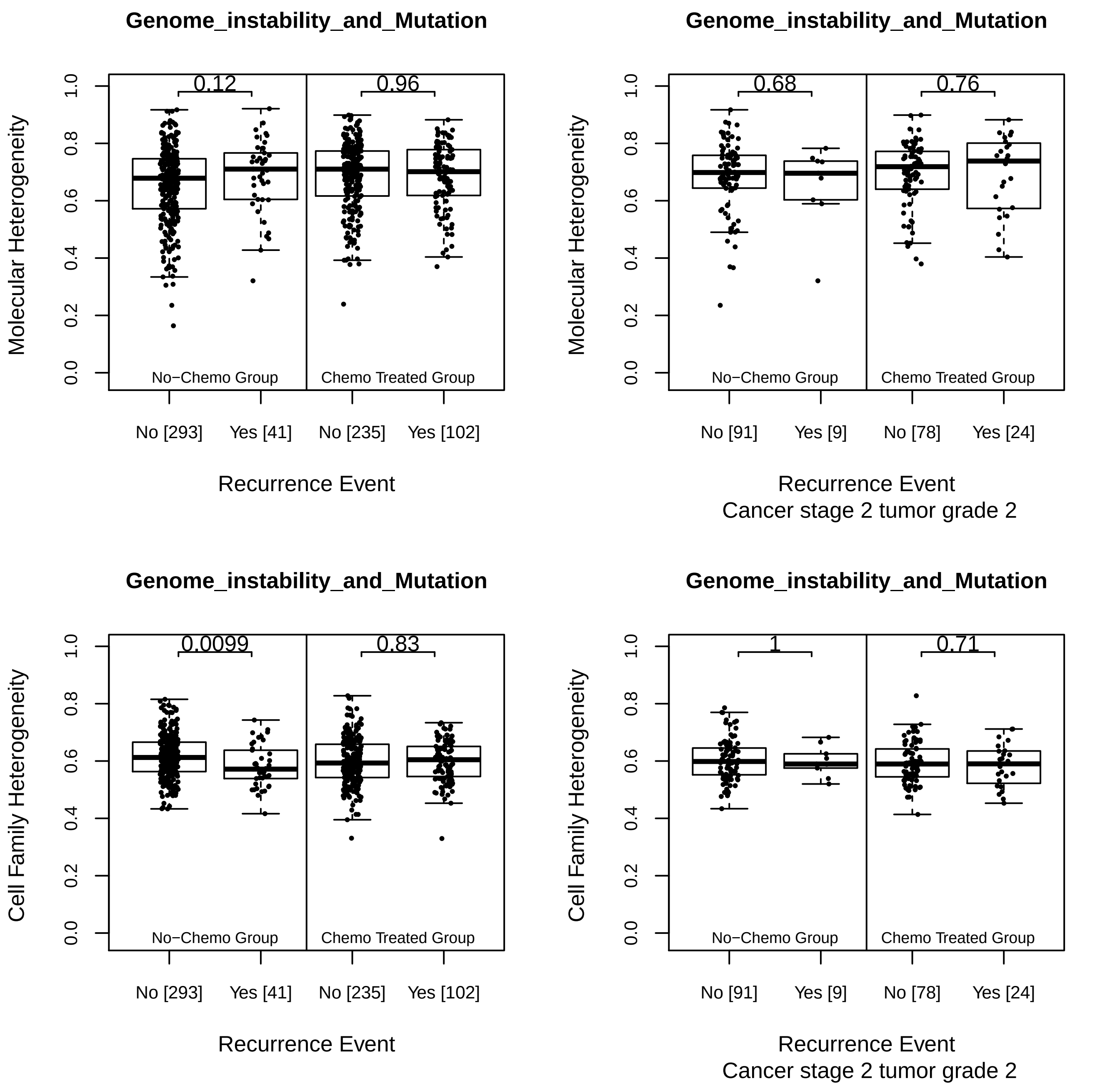
Genome_instability_and_Mutation
Molecular Heterogeneity
0.12
0.96
No−Chemo Group
Chemo Treated Group
No [293]
Yes [41]
No [235]
Yes [102]
Recurrence Event
Genome_instability_and_Mutation
Molecular Heterogeneity
0.68
0.76
No−Chemo Group
Chemo Treated Group
No [91]
Yes [9]
No [78]
Yes [24]
Recurrence Event
Cancer stage 2 tumor grade 2
Genome_instability_and_Mutation
Cell Family Heterogeneity
0.0099
0.83
No−Chemo Group
Chemo Treated Group
No [293]
Yes [41]
No [235]
Yes [102]
Recurrence Event
Genome_instability_and_Mutation
Cell Family Heterogeneity
1
0.71
No−Chemo Group
Chemo Treated Group
No [91]
Yes [9]
No [78]
Yes [24]
Recurrence Event
Cancer stage 2 tumor grade 2

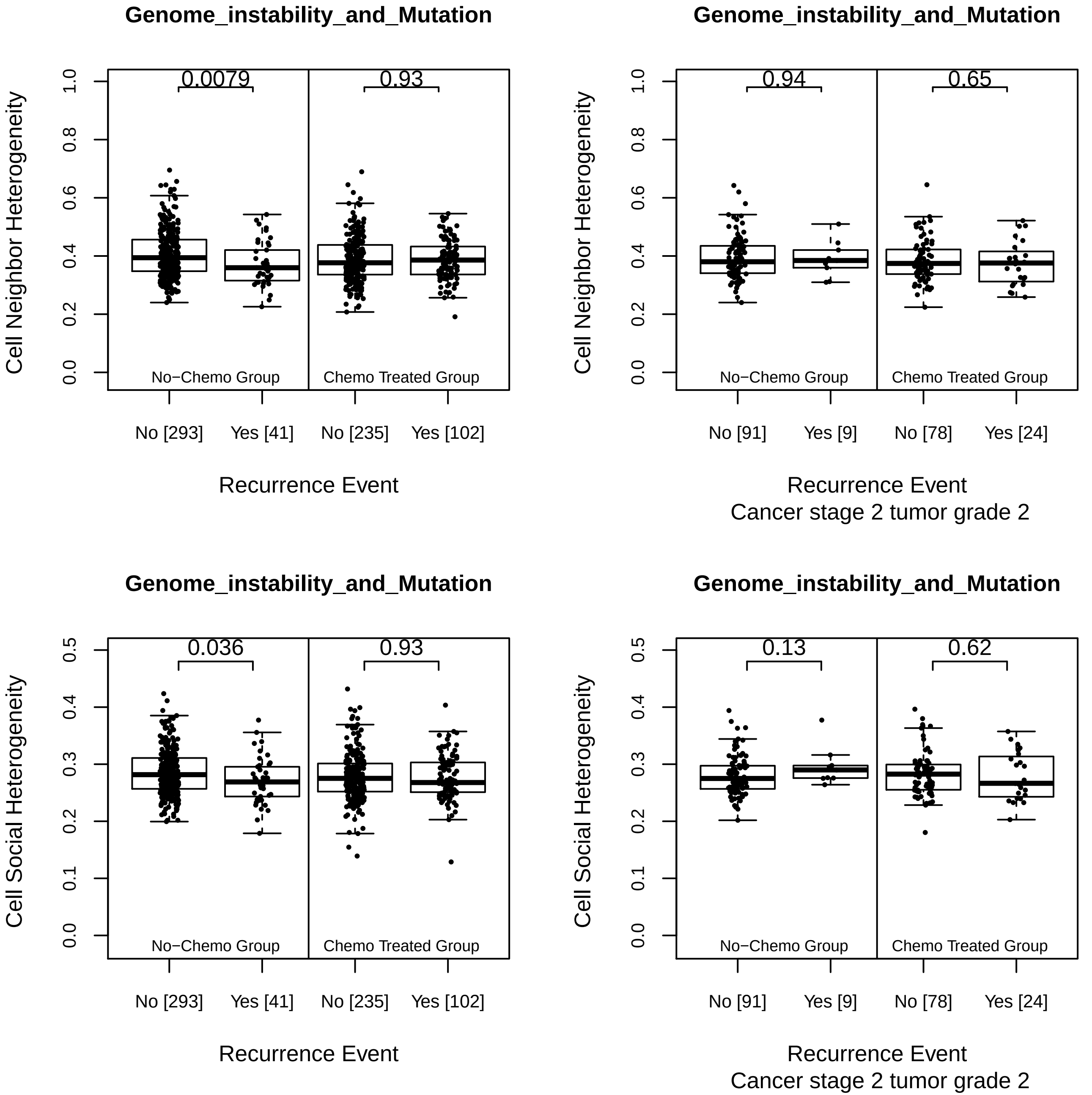
Genome_instability_and_Mutation
Cell Neighbor Heterogeneity
0.0079
0.93
No−Chemo Group
Chemo Treated Group
No [293]
Yes [41]
No [235]
Yes [102]
Recurrence Event
Genome_instability_and_Mutation
Cell Neighbor Heterogeneity
0.94
0.65
No−Chemo Group
Chemo Treated Group
No [91]
Yes [9]
No [78]
Yes [24]
Recurrence Event
Cancer stage 2 tumor grade 2
Genome_instability_and_Mutation
Cell Social Heterogeneity
0.036
0.93
No−Chemo Group
Chemo Treated Group
No [293]
Yes [41]
No [235]
Yes [102]
Recurrence Event
Genome_instability_and_Mutation
Cell Social Heterogeneity
0.13
0.62
No−Chemo Group
Chemo Treated Group
No [91]
Yes [9]
No [78]
Yes [24]
Recurrence Event
Cancer stage 2 tumor grade 2

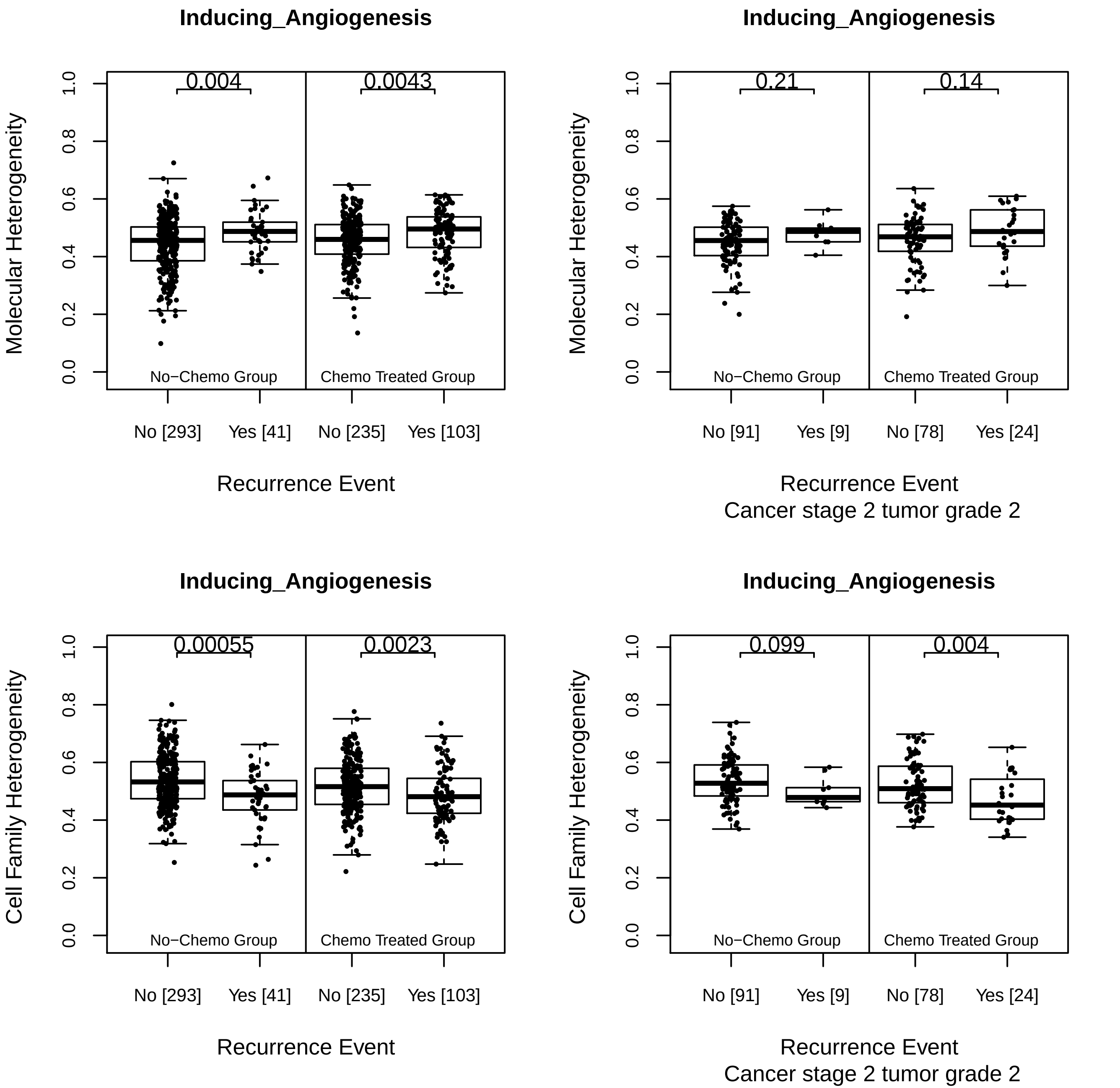
Inducing_Angiogenesis
Molecular Heterogeneity
1.0
0.8
0.6
0.4
0.2
0.0
0.004
0.0043
No−Chemo Group
Chemo Treated Group
No [293]
Yes [41]
No [235]
Yes [103]
Recurrence Event
Inducing_Angiogenesis
Molecular Heterogeneity
0.21
0.14
No−Chemo Group
Chemo Treated Group
No [91]
Yes [9]
No [78]
Yes [24]
Recurrence Event
Cancer stage 2 tumor grade 2
Inducing_Angiogenesis
Cell Family Heterogeneity
0.00055
0.0023
No−Chemo Group
Chemo Treated Group
No [293]
Yes [41]
No [235]
Yes [103]
Recurrence Event
Inducing_Angiogenesis
Cell Family Heterogeneity
0.099
0.004
No−Chemo Group
Chemo Treated Group
No [91]
Yes [9]
No [78]
Yes [24]
Recurrence Event
Cancer stage 2 tumor grade 2

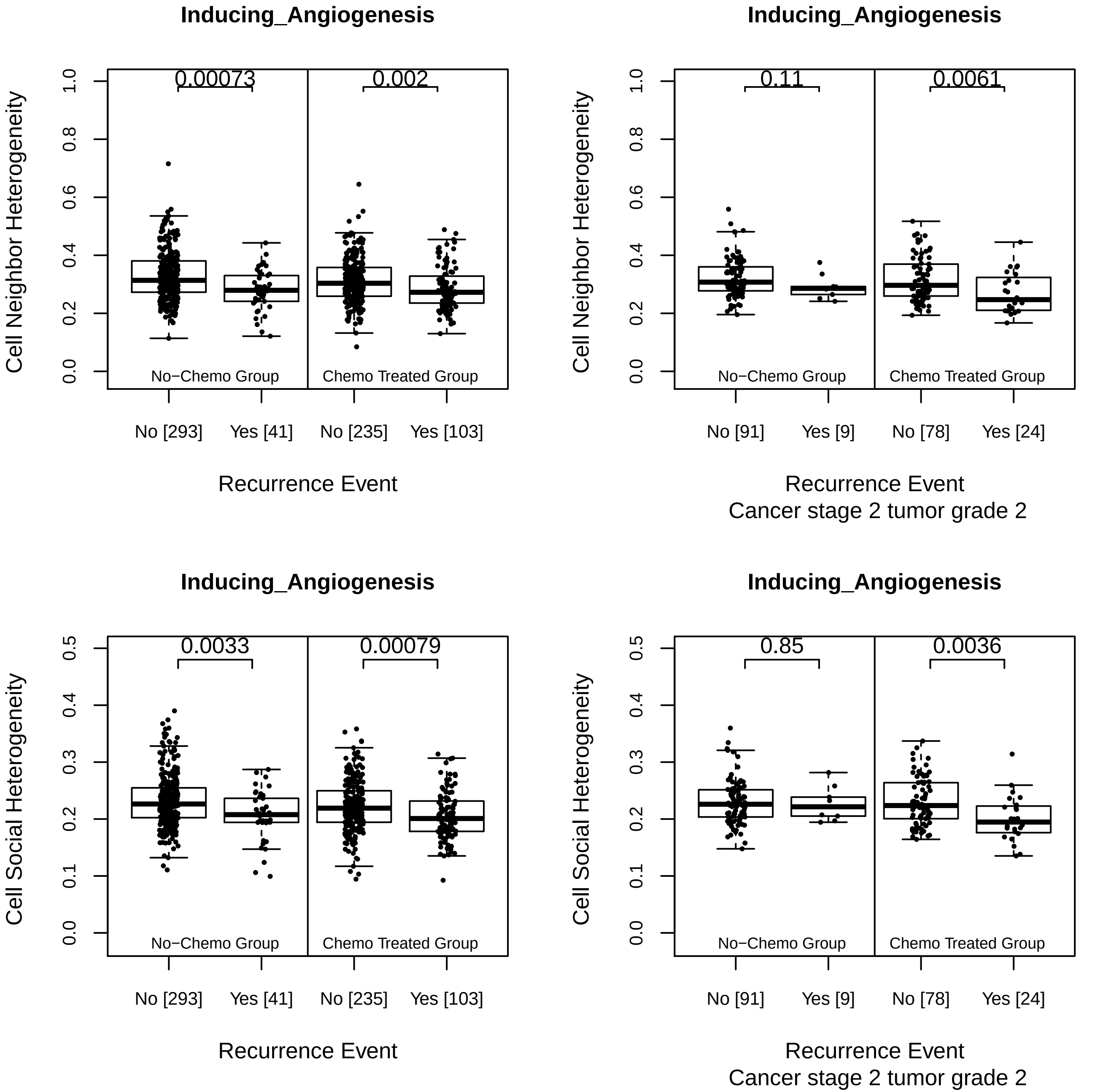
Inducing_Angiogenesis
Cell Neighbor Heterogeneity
0.0
0.2
0.4
0.6
0.8
1.0
0.00073
0.002
No−Chemo Group
Chemo Treated Group
No [293]
Yes [41]
No [235]
Yes [103]
Recurrence Event
Inducing_Angiogenesis
Cell Neighbor Heterogeneity
0.11
0.0061
No−Chemo Group
Chemo Treated Group
No [91]
Yes [9]
No [78]
Yes [24]
Recurrence Event
Cancer stage 2 tumor grade 2
Inducing_Angiogenesis
Cell Social Heterogeneity
0.0
0.1
0.2
0.3
0.4
0.5
0.0033
0.00079
No−Chemo Group
Chemo Treated Group
No [293]
Yes [41]
No [235]
Yes [103]
Recurrence Event
Inducing_Angiogenesis
Cell Social Heterogeneity
0.85
0.0036
No−Chemo Group
Chemo Treated Group
No [91]
Yes [9]
No [78]
Yes [24]
Recurrence Event
Cancer stage 2 tumor grade 2

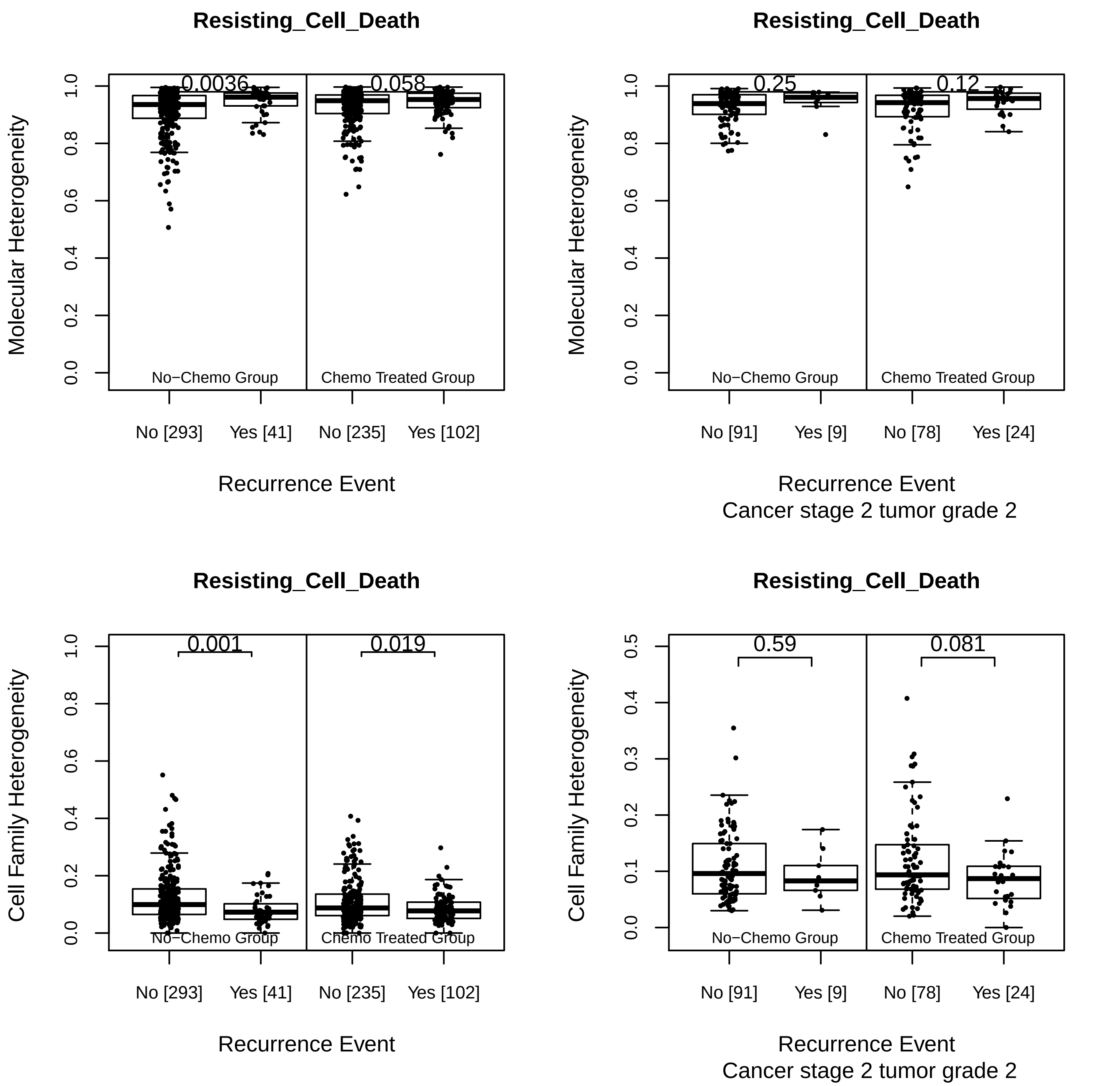
Resisting_Cell_Death
Molecular Heterogeneity
0.0036
0.058
No−Chemo Group
Chemo Treated Group
No [293]
Yes [41]
No [235]
Yes [102]
Recurrence Event
Resisting_Cell_Death
Molecular Heterogeneity
0.25
0.12
No−Chemo Group
Chemo Treated Group
No [91]
Yes [9]
No [78]
Yes [24]
Recurrence Event
Cancer stage 2 tumor grade 2
Resisting_Cell_Death
Cell Family Heterogeneity
0.001
0.019
No−Chemo Group
Chemo Treated Group
No [293]
Yes [41]
No [235]
Yes [102]
Recurrence Event
Resisting_Cell_Death
Cell Family Heterogeneity
0.59
0.081
No−Chemo Group
Chemo Treated Group
No [91]
Yes [9]
No [78]
Yes [24]
Recurrence Event
Cancer stage 2 tumor grade 2

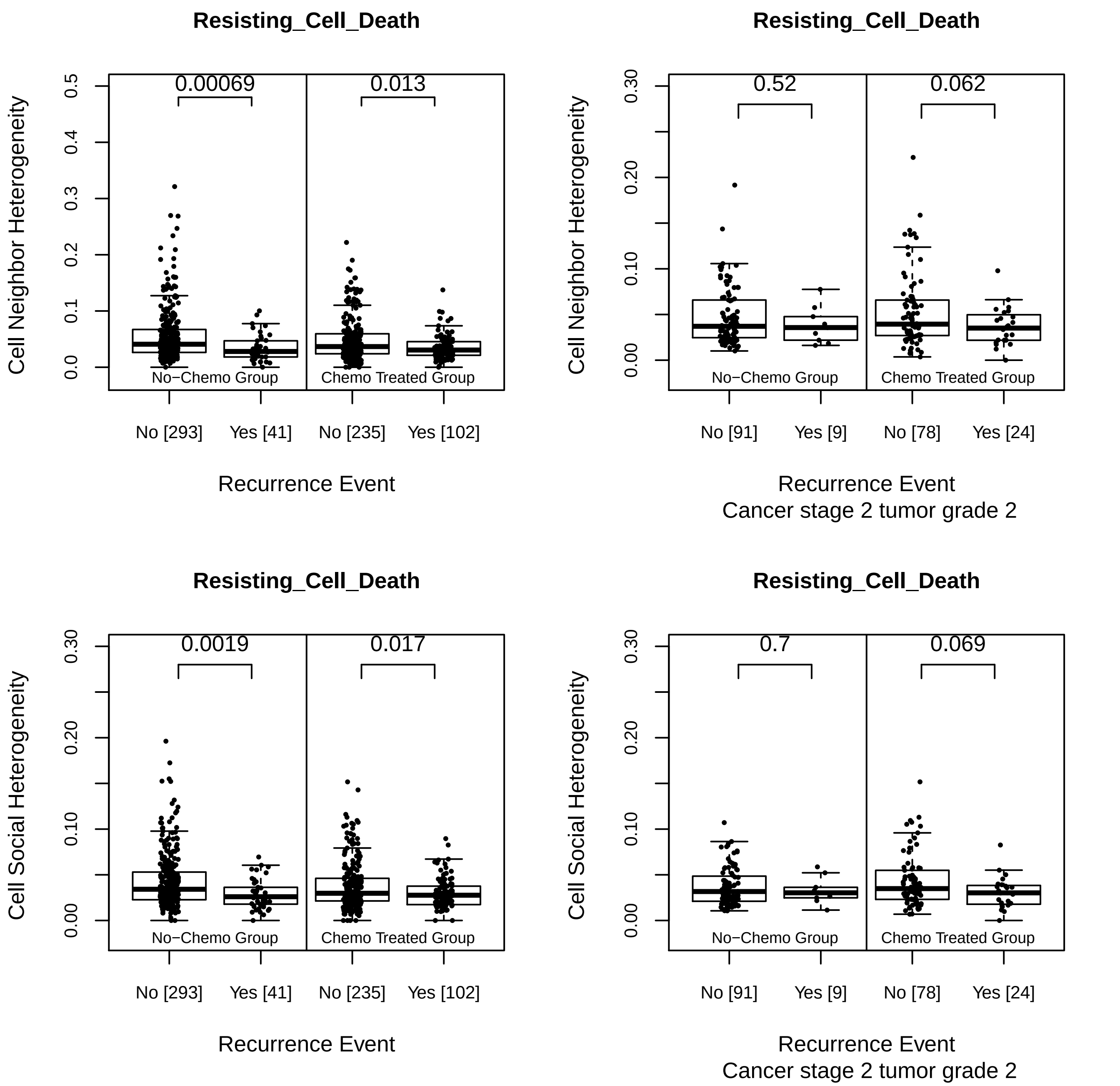
Resisting_Cell_Death
Cell Neighbor Heterogeneity
0.00069
0.013
No−Chemo Group
Chemo Treated Group
No [293]
Yes [41]
No [235]
Yes [102]
Recurrence Event
Resisting_Cell_Death
Cell Neighbor Heterogeneity
0.52
0.062
No−Chemo Group
Chemo Treated Group
No [91]
Yes [9]
No [78]
Yes [24]
Recurrence Event
Cancer stage 2 tumor grade 2
Resisting_Cell_Death
Cell Social Heterogeneity
0.0019
0.017
No−Chemo Group
Chemo Treated Group
No [293]
Yes [41]
No [235]
Yes [102]
Recurrence Event
Resisting_Cell_Death
Cell Social Heterogeneity
0.7
0.069
No−Chemo Group
Chemo Treated Group
No [91]
Yes [9]
No [78]
Yes [24]
Recurrence Event
Cancer stage 2 tumor grade 2

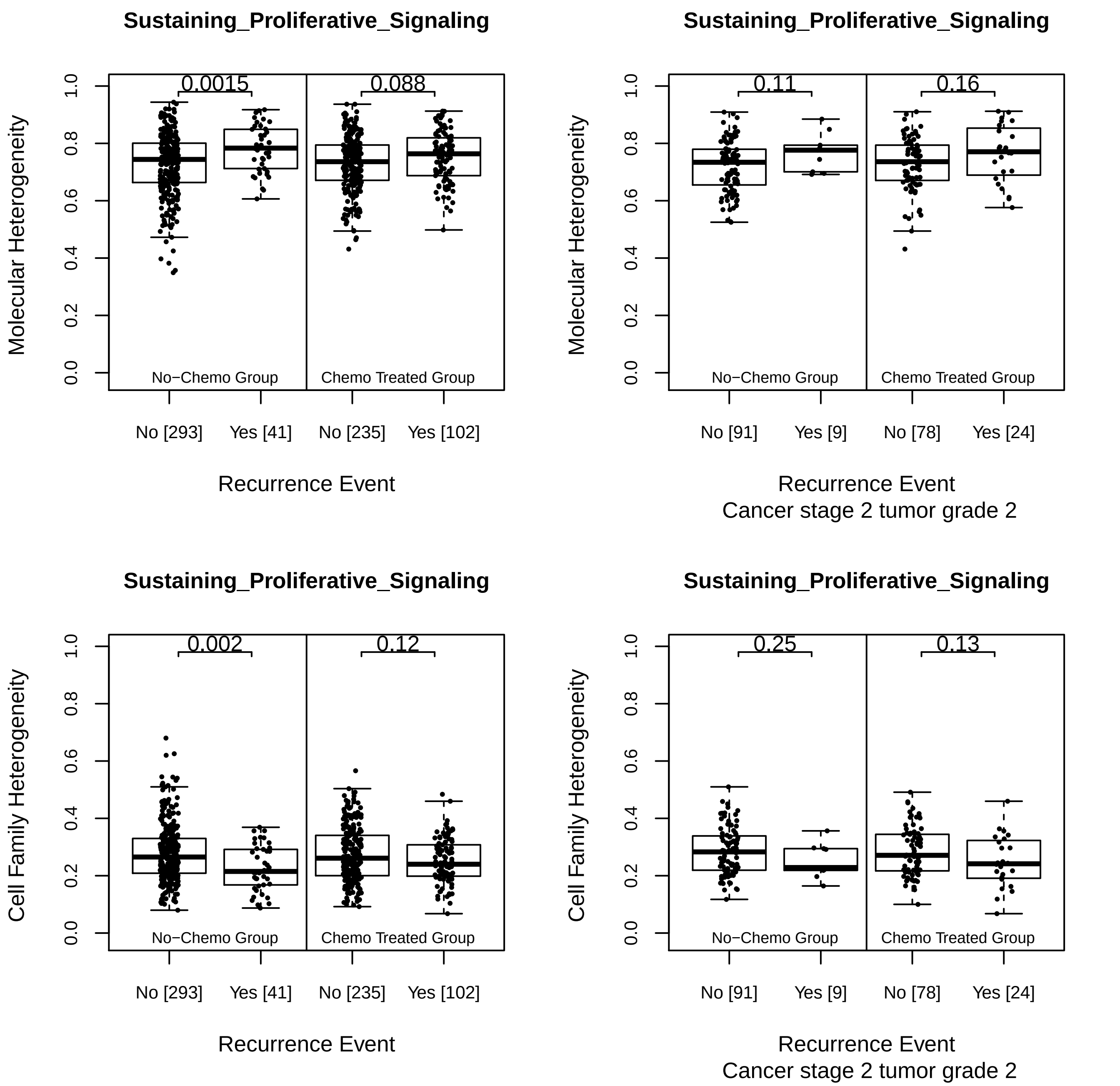

Sustaining_Proliferative_Signaling
Molecular Heterogeneity
0.0015
0.088
No−Chemo Group
Chemo Treated Group
No [293]
Yes [41]
No [235]
Yes [102]
Recurrence Event
Sustaining_Proliferative_Signaling
Molecular Heterogeneity
0.11
0.16
No−Chemo Group
Chemo Treated Group
No [91]
Yes [9]
No [78]
Yes [24]
Recurrence Event
Cancer stage 2 tumor grade 2
Sustaining_Proliferative_Signaling
Cell Family Heterogeneity
0.002
0.12
No−Chemo Group
Chemo Treated Group
No [293]
Yes [41]
No [235]
Yes [102]
Recurrence Event
Sustaining_Proliferative_Signaling
Cell Family Heterogeneity
0.25
0.13
No−Chemo Group
Chemo Treated Group
No [91]
Yes [9]
No [78]
Yes [24]
Recurrence Event
Cancer stage 2 tumor grade 2

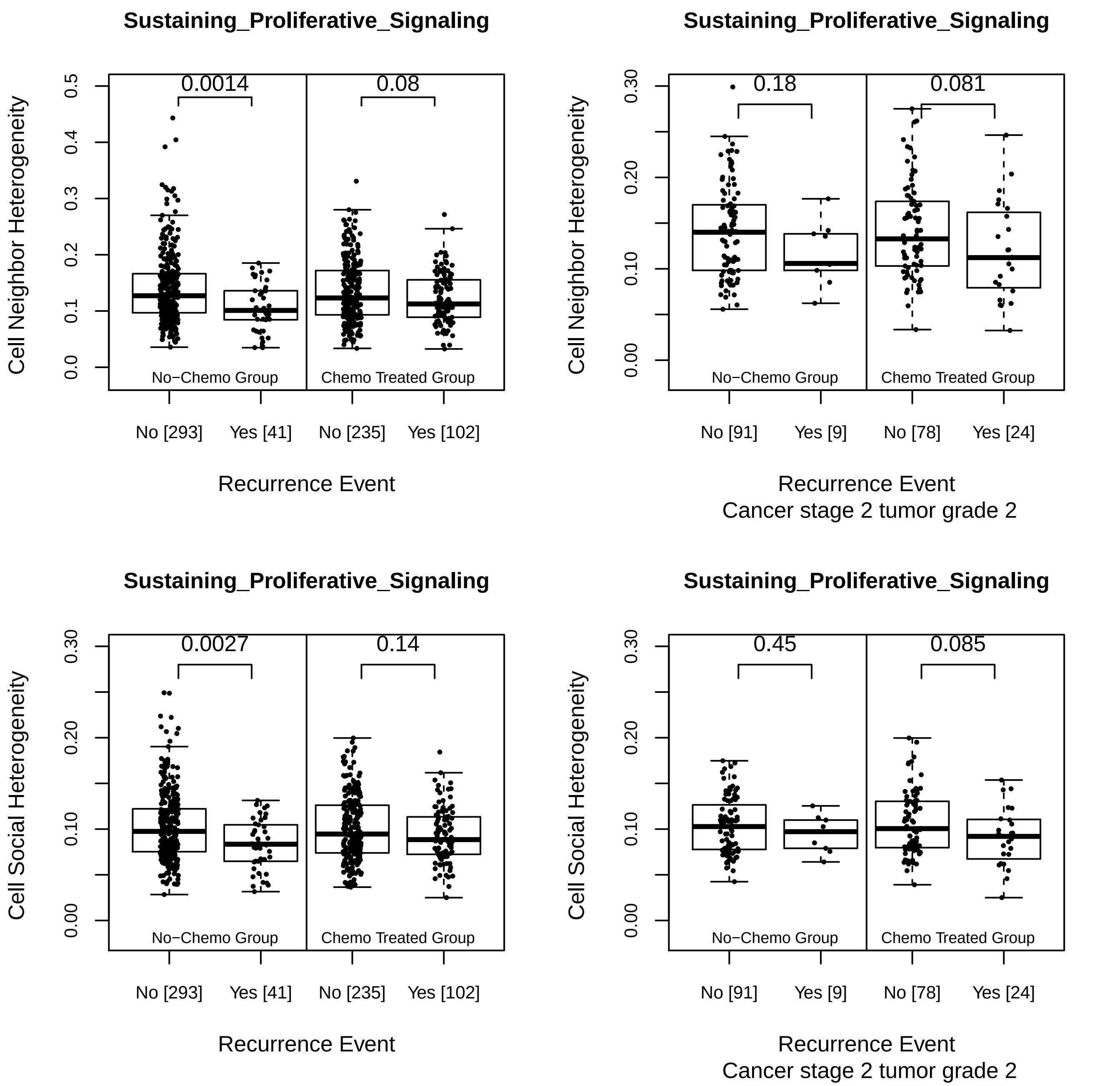
Sustaining_Proliferative_Signaling
Cell Neighbor Heterogeneity
0.0
0.1
0.2
0.3
0.4
0.5
0.0014
0.08
No−Chemo Group
Chemo Treated Group
No [293]
Yes [41]
No [235]
Yes [102]
Recurrence Event
Sustaining_Proliferative_Signaling
Cell Neighbor Heterogeneity
0.00
0.10
0.20
0.30
0.18
0.081
No−Chemo Group
Chemo Treated Group
No [91]
Yes [9]
No [78]
Yes [24]
Recurrence Event
Cancer stage 2 tumor grade 2
Sustaining_Proliferative_Signaling
Cell Social Heterogeneity
0.00
0.10
0.20
0.30
0.0027
0.14
No−Chemo Group
Chemo Treated Group
No [293]
Yes [41]
No [235]
Yes [102]
Recurrence Event
Sustaining_Proliferative_Signaling
Cell Social Heterogeneity
0.00
0.10
0.20
0.30
0.45
0.085
No−Chemo Group
Chemo Treated Group
No [91]
Yes [9]
No [78]
Yes [24]
Recurrence Event
Cancer stage 2 tumor grade 2

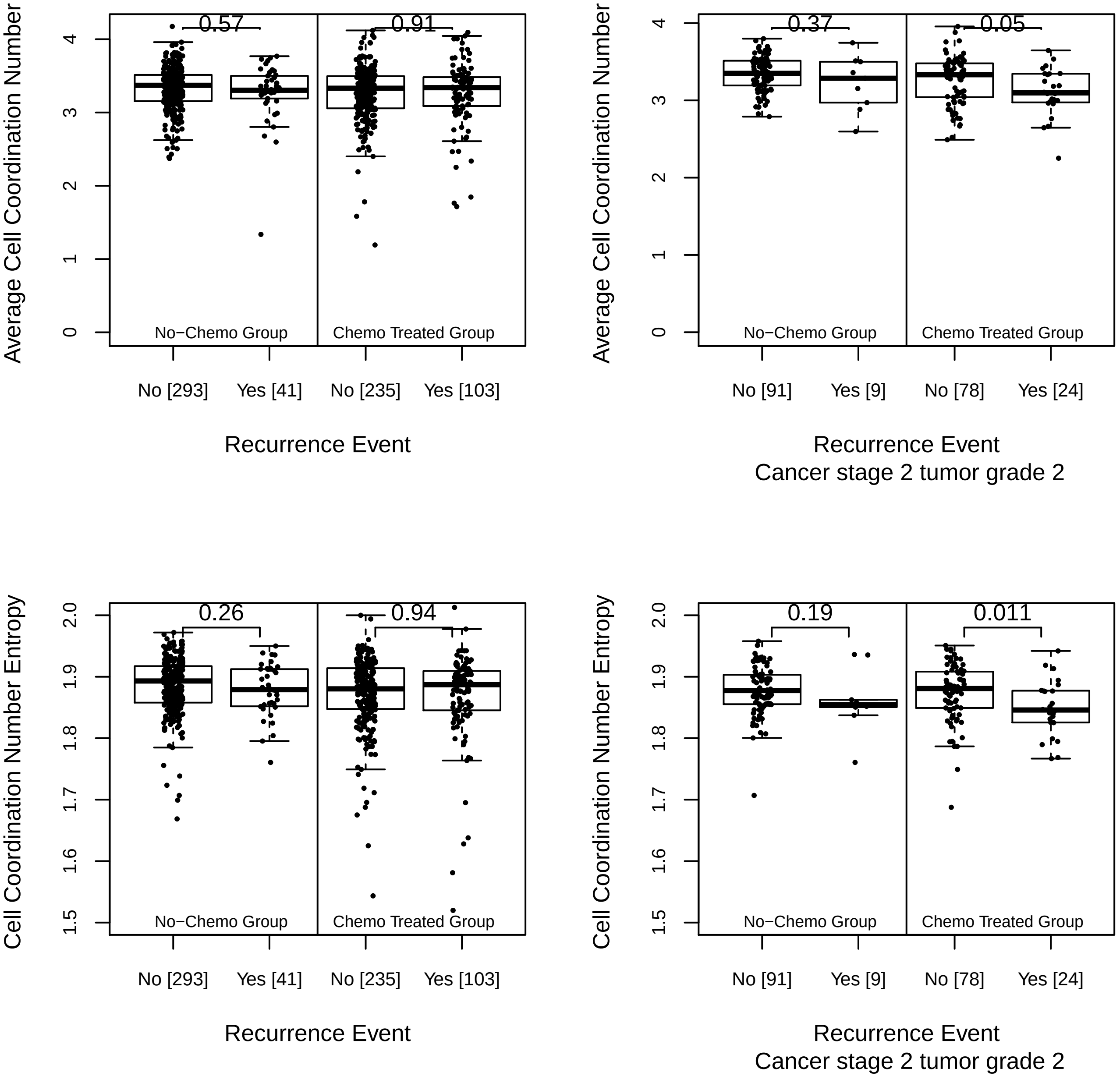

Average Cell Coordination Number
0.57
0.91
No−Chemo Group
Chemo Treated Group
No [293]
Yes [41]
No [235]
Yes [103]
Recurrence Event
Average Cell Coordination Number
0.37
0.05
No−Chemo Group
Chemo Treated Group
No [91]
Yes [9]
No [78]
Yes [24]
Recurrence Event
Cancer stage 2 tumor grade 2
Cell Coordination Number Entropy
0.26
0.94
No−Chemo Group
Chemo Treated Group
No [293]
Yes [41]
No [235]
Yes [103]
Recurrence Event
Cell Coordination Number Entropy
0.19
0.011
No−Chemo Group
Chemo Treated Group
No [91]
Yes [9]
No [78]
Yes [24]
Recurrence Event
Cancer stage 2 tumor grade 2

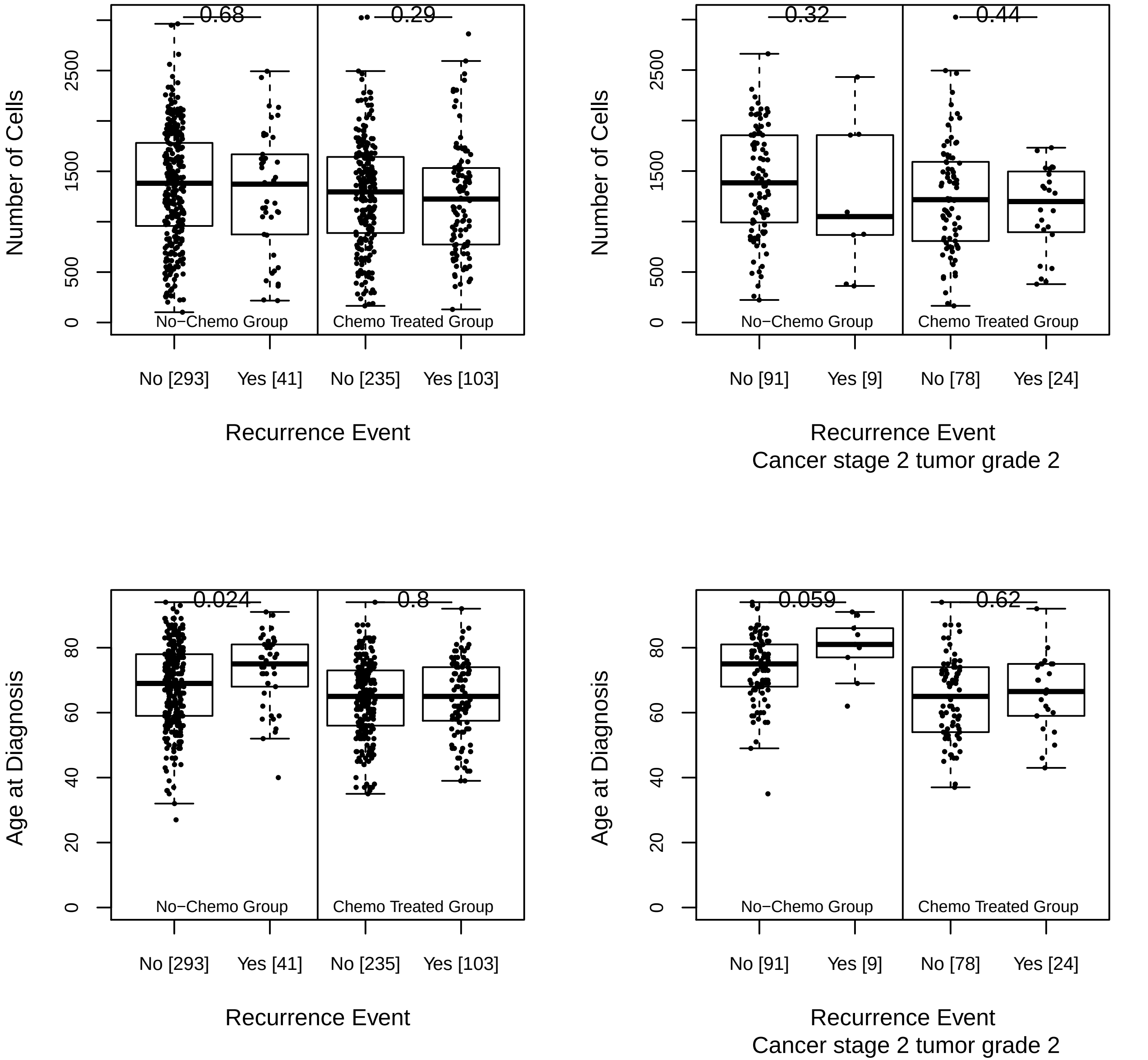
0.68
0.29
0.32
0.44
Number of Cells
0
500
1500
2500
No−Chemo Group
Chemo Treated Group
No [293]
Yes [41]
No [235]
Yes [103]
Recurrence Event
No [91]
Yes [9]
No [78]
Yes [24]
Recurrence Event
Cancer stage 2 tumor grade 2
0.024
0.8
0.059
0.62
Age at Diagnosis
0
20
40
60
80
Recurrence Event
Cancer stage 2 tumor grade 2

Section 7

Frequency distributions of cell coordination numbers and by cancer stage and tumor grade.

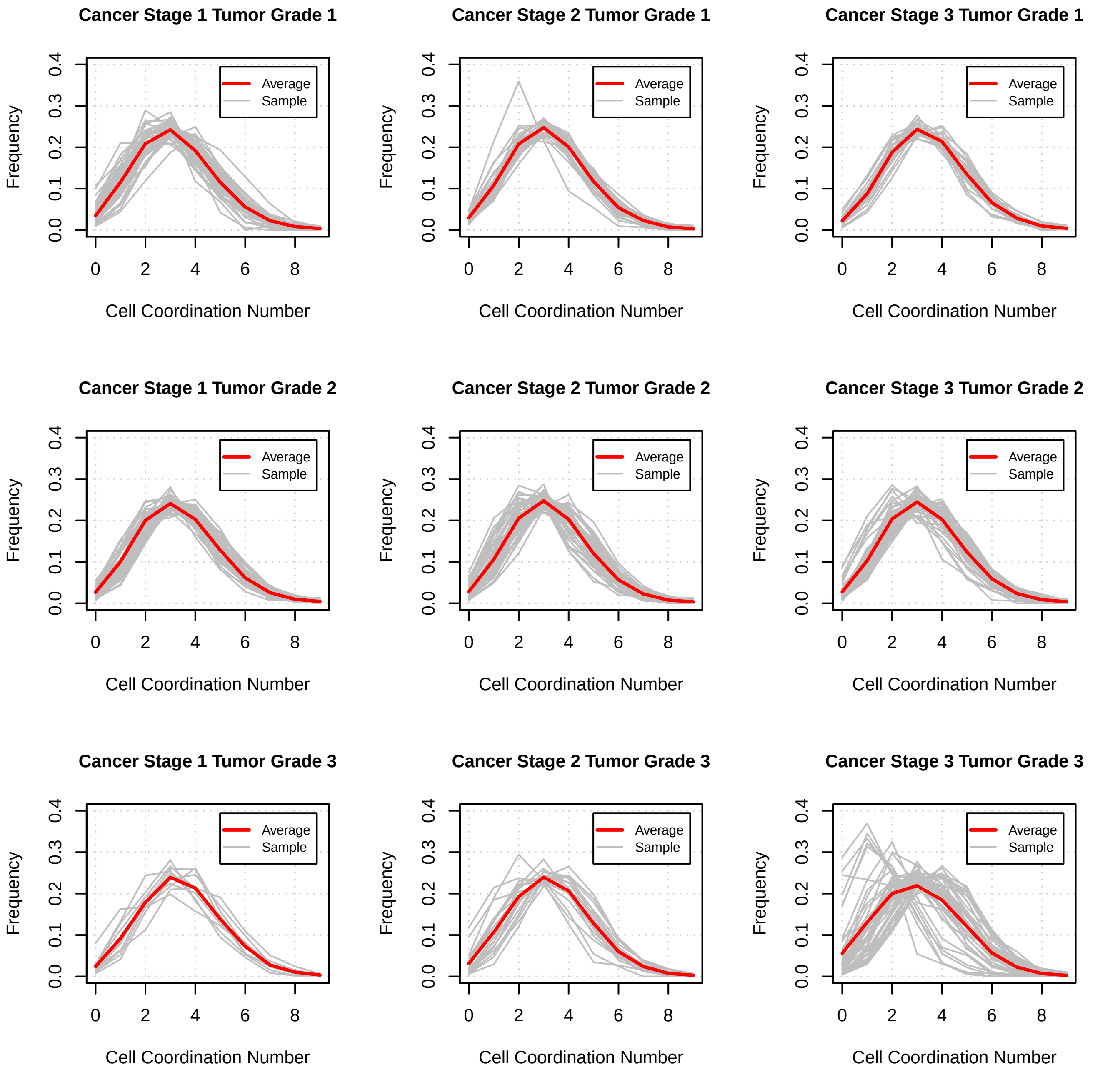

Cancer Stage 1 Tumor Grade 1
Cancer Stage 2 Tumor Grade 1
Cancer Stage 3 Tumor Grade 1
Cancer Stage 1 Tumor Grade 2
Cancer Stage 2 Tumor Grade 2
Cancer Stage 3 Tumor Grade 2
Cancer Stage 1 Tumor Grade 3
Cancer Stage 2 Tumor Grade 3
Cancer Stage 3 Tumor Grade 3
Average
Sample
Frequency
0.0
0.1
0.2
0.3
0.4
0
2
4
6
8
Cell Coordination Number
